# Complete Genomes of Human Papillomavirus Type 16 Viruses Isolated from Cases of Cervical Neoplasia and Squamous Cell Carcinomas Followed in Latvia in 2012–2024

**DOI:** 10.3390/vaccines14060517

**Published:** 2026-06-09

**Authors:** Juris Jansons, Nikita Zrelovs, Arta Spridzane, Marija Nazarenko, Liba Sokolovska, Karina Biserova, Daira Krisane, Austra Breiksa-Vaivode, Daria Avdoshina, Beatrise Orlova, Marta Petrovska, Serhii Kalman, Stefan Petkov, Valery Ilinsky, Anna Ilinskaya, Jurijs Nazarovs, Androniks Mitildzans, Maria Isaguliants

**Affiliations:** 1 Institute of Microbiology and Virology, Riga Stradins University, LV-1067 Riga, Latviamarija.nazarenko@rsu.lv (M.N.); karina.biserova@rsu.lv (K.B.); daryaavdoshina8@gmail.com (D.A.); 2 National Research and Innovation Institute, LV-1067 Riga, Latvia; 3 Scientific Laboratory of Molecular Genetics, Riga Stradins University, LV-1007 Riga, Latvia; 4 Latvian Oncology Center, Riga East Clinical University Hospital, LV-1079 Riga, Latvia; andronmit@gmail.com; 5 Pathology Institute, Pauls Stradins Clinical University Hospital, LV-1002 Riga, Latviaaustra.breiksa@stradini.lv (A.B.-V.);; 6 Centrala Laboratorija Ltd., LV-1019 Riga, Latvia; beatrise.orlova@laboratorija.lv (B.O.);; 7 R.E. Kavetsky Institute of Experimental Pathology, Oncology and Radiobiology, National Academy of Sciences of Ukraine, 03022 Kyiv, Ukraine; kalmansergej12@gmail.com; 8 Department of Microbiology, Tumor and Cell Biology, Karolinska Institutet, 17177 Stockholm, Sweden; 9 Eligens, Human Genetics, Digital Health, Eligens SIA, LV-2167 Marupe, Latviaanna@eligens.io (A.I.); 10 Department of Pathology, Riga Stradins University, LV-1007 Riga, Latvia

**Keywords:** human papillomavirus type 16 (HPV16), cervical squamous cell carcinoma, cervical neoplasia, whole genome sequencing, oncoprotein E6, selection, co-variance analysis, HPV vaccine escape

## Abstract

**Background:** Persistent high-risk human papillomavirus (hrHPV) infection causes over 99% of cervical precancers and cancers worldwide, with HPV genotype 16 (HPV16) responsible for 50% of the cases. Latvia ranks among the top EU countries for cervical cancer incidence and mortality. In the general Latvian population, 4.2% of women are hrHPV-infected, mostly with HPV16. However, information on the circulating HPV16 isolates is missing. **Objectives**: To study the genomic variability of the Latvian HPV16 isolates, compare them with HPV16 in Europe and across the globe, reveal features associated with the severity of cervical disease and uncover eventual sequence changes due to the national HPV vaccination. **Methods:** DNA was extracted from the formalin-fixed paraffin-embedded cervical tissues of women diagnosed with cervical intraepithelial neoplasia (CIN) stages I-III and squamous cell carcinoma (SCC) grades 1–3, collected between 2012 and 2024. Samples positive for HPV16 were subjected to whole genome sequencing (WGS) on the Illumina platform (n = 16) or Sanger sequencing of the E6/E7 coding region (n = 31). A consensus HPV16 sequence was generated, and single nucleotide polymorphisms (SNPs) and eventual amino acid substitutions (AAS) were analysed. **Results:** Complete genomes of 16 HPV16 variants were reconstructed, with 13 related to the European sublineage A1 and 3 to the sublineage A2 references. Sequences showed high conservation; still 93 non-redundant variants were identified. The highest variability was observed for the capsid protein L2, and the lowest, for oncoprotein E7. The prevalence of SNPs and AAS in the Latvian HPV16 variants, specifically in capsid protein L1, did not increase with time, showing no effect of HPV vaccination. Associations between HPV16 sequence features and severity of cervical disease were limited to AAS E6:L90V, which was significantly more common in SCC grade 2/3 than in CINII/III cases (*p* = 0.015). **Conclusions:** Highly conserved HPV16 genomes circulating in Latvia harbour a series of unique as well as common nonsynonymous SNPs with respective AAS, with one, AAS E6:L90V, associating with disease severity. No HPV vaccine escape variants were detected. Deciphering complete genomes of HPV16 from CIN and SCC cases in Latvia informs public authorities performing HPV vaccination and is useful for the management of HPV-associated cervical diseases.

## 1. Introduction

According to the World Health Organization, cervical cancer, with cervical squamous cell carcinoma (SCC) being the most common histological type accounting for roughly 70% to over 80% of the cases, is a major global health issue, with hundreds of thousands of new cases and deaths registered annually worldwide [1]. The majority of the cervical cancer (CC) cases is associated with infection with human papillomaviruses (HPVs) [2]. Based on recent studies and data from the International HPV Reference Center, there are 200 to 230 officially recognized human papillomavirus (HPV) genotypes [3], which differ in their oncogenic potential. Fourteen, namely HPV 16, 18, 31, 33, 35, 39, 45, 51, 52, 56, 58, 59, 66, and 68, cause intraepithelial lesions which may progress to cancer and are classified as the high-risk HPVs (hrHPVs). Infections with these hrHPVs cause nearly 100% (99.7%) of CC cases [4,5]. The highest risk is ascribed to HPV 16 and 18, which together account for nearly 70% of all invasive cervical cancers worldwide [6]. HPV16 alone accounts for approximately 50% of CC cases [7,8].

HPV vaccination has significantly reduced the burden of HPV infections. A systematic review and meta-analysis, including data from 60 million individuals and up to 8 years of post-vaccination follow-up, provided evidence for the substantial impact of HPV vaccination programmes on HPV infections. After 5–8 years of vaccination, the prevalence of HPV 16 and 18 among girls aged 13–19 years decreased by 83%, and among women aged 20–24 years, by 66% [9]. In countries with >90% HPV vaccination coverage, the prevalence of HPV16/18 has reduced to less than 1% (see, for example, [10]). At the same time, in countries with low adherence to HPV vaccination, HPV16 prevalence remains high (up to 20%), often mirroring the pre-vaccination era [11,12]. Furthermore, both countries not performing HPV vaccination and countries with insufficient HPV vaccine coverage are predicted to experience regional increases in HPV16 prevalence [13,14].

In countries with limited access to health care, no HPV vaccine access and/or low vaccination coverage cervical cancer will remain a serious public health concern [15,16], with nearly 95% of SCC cases attributable to hrHPV infection [17,18]. Alarmingly, the most recent data suggests an increasing involvement of HPV16 in the development of other cervical cancer forms, such as adenocarcinoma in situ, adenosquamous carcinomas and various adenocarcinoma subtypes [19,20]. This phenomenon involves not only different cervical cancer histotypes but also other cancer forms, such as anogenital and oropharyngeal cancers, which all reveal an increasing prevalence of HPV16 [21]. Another phenomenon is an increase in the prevalence of HPV16 (genital, anal, oral) in certain age groups, such as women over 36 years old [22,23], men [24], specifically aged [24] and men who have sex with men [25]. These data indicate that despite global HPV vaccination efforts, HPV16 remains and will remain a serious threat to human health and a heavy burden for public health care systems.

Latvia is a country with a high HPV prevalence; about 4.2% of women in the general population harbour HPV16/18 infection [26]. The country has one of the highest cervical cancer incidence and mortality rates in Europe, accompanied by relatively low screening rates and low HPV vaccine acceptance and uptake [27]. Of the invasive CC cases, 77.0% are attributed to HPV16/18 [26]. HPV vaccination started in Latvia for girls in 2010, and universal vaccination of adolescents in 2020. Today, 15 years later, screening and vaccination are not yet sufficiently efficient. HPV vaccine coverage is 43% for the 1st and 42% for the last dose (2023). To compare, in Sweden, coverage by the 1st dose is 87% and by the last, 82%, and only 2.4% of women are estimated to harbour HPV16/18 [12]. Alarmingly, Latvia is also the country with the lowest level of agreement that the HPV vaccine is safe in the EU—only 55.6%, whereas in Portugal, the same rate constitutes 89% [28].

In these settings, hrHPVs, specifically HPV16, will continue to spread and prevail in the Latvian population. The latest cross-sectional study conducted in Latvia from February 2021 to April 2022 demonstrated a prevalence of any hrHPV infection of 66.8% in the colposcopy group and 11.0% in the general population [29] (much higher than stated earlier) [26]. Among 9810 HPV-positive cervical samples from women aged 30 to 70 years, HPV 16 is the most prevalent genotype, representing 15.63% of all detected HPV genotypes and nearly 20% of HPV-positive samples [30]. We found HPV16 to be the most prevalent genotype also in cervical disease; it was detected in >90% of cases of cervical cancer and dysplasia observed in Riga, Latvia, during 2016–2024, with disease severity increasing with increasing HPV16 viral load [31]. This epidemiological landscape, combined with vaccine hesitancy [32], predicts wide circulation of HPV16 in the Latvian population over the next decade.

Despite the acuteness of the epidemiological situation, information on the genomic sequences of HPV16 variants circulating in Latvia is missing. The currently used HPV vaccine Gardasil 9 has the potential to prevent more than 90% of cervical cancers. Still, continuous monitoring of the circulating variants is needed to ensure that vaccine coverage remains effective against the new hrHPV variants, specifically those of HPV16 as the most prevalent [33]. In this study, we aimed to investigate the genomic variability of the Latvian HPV16 isolates, compare their genetic and protein makeup with that of HPV16 isolates from Europe and across the globe, and associate these features with the severity of cervical disease to aid diagnosis and prognosis of cervical pathologies. We also sought to eventually uncover HPV16 variants capable of HPV vaccine escape, to inform public health care systems on whether HPV vaccine coverage remains effective.

## 2. Materials and Methods

### 2.1. Study Group

The study was performed according to the permit of the Ethical Committee of Riga Stradins University (RSU) N2-PĒK-4/415/2022 dated 26 September 2022. Women visiting the gynaecologist (prospective cohort; n = 86) were conditionally healthy or diagnosed with CINI to CINIII, or CC grades 1 to 3 (n = 37). Patients gave written informed consent and entered the study under the study codes, with personal data accessible only to the gynaecologist performing the follow-up. Under the follow-up, series of cervical tissue samples were collected, including biopsies of cervical lesions, electroexision materials and tumours (upon ectopy). We also had access to a number of retrospective samples of the study participants collected before 2021, made available by the patients and/or the gynaecologist performing the follow-up.

An independent set of samples of cervical tissues belonging to women with CIN stages I to III and SCC of grades 1–3 (retrospective cohort; n = 145) was selected from the archive of the Paul Stradins University Clinical Hospital (Riga, Latvia). Data on the patients was limited to age, clinical diagnosis, and pathomorphological characteristics of the tissues. Written informed consents from these individuals were not obtained as this part of the study was retrospective and had a non-interventional character. As such, it did not add any additional risks to the patients, did not interfere with their routine diagnosis and treatment, and did not affect their medical rights. Samples were de-personified by the gynaecologist making the selection, with assignment of study codes.

None of the women in the prospective cohort was HPV vaccinated. In the retrospective cohort, information on HPV vaccination status was not available; however, none of the women could have been HPV vaccinated in the course of the public HPV vaccination campaign, as all were older than 17 years in 2010 when the HPV vaccination program in Latvia was started.

### 2.2. Isolation of DNA, hrHPV Genotyping and Quality Check

All cervical tissues represented formalin-fixed and paraffin-embedded (FFPE) blocks. The FFPE blocks were cut into 5 μm sections. Part of sections was hematoxylin/eosin-stained and subjected to histopathological screening to grade the lesion. The other part was treated to extract DNA (QIAamp DNA FFPE Advanced Kit, Quiagen, Venlo, Netherlands) as described in [34].

DNA was subjected to commercial PCR tests detecting 14 hrHPV genotypes using either the semiquantitative AnyPlex II kit, providing data on virus load as high (+++), medium (++) and low (+) (Seegene, Seoul, Republic of Korea; performed by E. Gulbis Laboratory, Riga, Latvia, https://www.egl.lv/, or the quantitative AllPlex kit, providing virus load as Ct (Seegene; performed by the Central Laboratory, Riga, Latvia; https://www.laboratorija.lv). Both test systems were clinically validated according to the guidelines for HPV test requirements for cervical cancer screening [35,36,37,38].

DNA samples from tissues found to contain high or medium HPV16 loads were selected and subjected to a quality check. DNA quality check was done by the fluorescence-based quantification method and fragment length analysis, as was described earlier [39]. Of the samples with medium to high content of HPV16 (Ct values < 31 according to Oštrbenk Valenčak A. et al. 2018 and 2024 [35,36]), 16 from the prospective cohort were suitable for WGS (Table 1) and 31 from the retrospective cohort were suitable for Sanger sequencing (Table 2).

### 2.3. Whole Genome Sequencing (WGS)

The whole genome sequencing running WGS protocol for the samples PV809647-PV809650, PV809661 and PV809662 (Table 1) was performed in kind by Eligens Ltd. (Marupe, Latvia) on the llumina HiSeq2500 platform (read length: 2 × 100 bp, output: 10 Gb per sample). The whole genome sequencing for the samples PV809651–PV809660 (Table 1) was performed by CeGaT (Tuebingen, Germany) on the Illumina NovaSeq 6000 platform (read length: 2 × 100 bp, output: 52 Gb per sample). On the operator’s recommendation, the PCR amplification step was included in the library preparation, considering the possible partial degradation of the DNA matrix. Data provided by the companies in FASTQ format was subjected to demultiplexing and adapter trimming.

### 2.4. Sanger Sequencing

The E6/E7 coding region of DNAs extracted from the retrospective patient DNA was PCR-amplified using primer pairs: (1) E6E7-53_HPV16 FW 5′-GAAACCGGTTAGTATAAAAGCAGAC-3′ and E6/7.2_603_HPV16 RW 5′-GAGATCAGTTGTCTCGGTTGCAAA-3′, and (2) E6/7.3_531_HPV16 FW 5′-CAAGAACACGTAGAGAAACCCAG-3′ and E6E7-923 HPV16 RW 5′-TTTTTCCACTACAGCCTCTACAT-3′. The outer primer pair was described earlier [40]. Amplified HPV16 DNA fragments were sequenced directly using the ABI Prism BigDye™ Terminator v3.1 Cycle Sequencing Kit (Applied Biosystems, Foster City, CA, USA), using PCR primers as sequencing primers. The electropherograms were obtained on an ABI Prism 377 sequencer (Applied Biosystems, Foster City, CA, USA). The nucleotide sequences were processed using the BioEdit 7.7.1 program [41] (Informer Technologies, Inc., Los Angeles, CA, USA) and deposited into the GenBank BioProject PRJNA1293798, accession numbers PQ215484 to PQ215514 (Table 2; Supplementary Table S1).

### 2.5. Reconstruction of the Consensus Sequence of Latvian HPV16 Isolates

For each of the 16 WGS belonging to the prospective cohort, the corresponding demultiplexed paired-read dataset with the adapters trimmed off was received from the service provider, along with the detailed quality report. No quality trimming was deemed necessary upon inspection of the report. The following steps were performed on a per-sample basis. First, the reads were mapped onto the HPV16-extended human genome reference (hg19, with the HPV16 reference available under the following accession NC_001526.4 added as an extra contig) using the Burrows–Wheeler Aligner (BWA-MEM algorithm under default settings, v.0.7.17-r1188 [42]). The generated binary alignment maps were sorted, indexed, and processed to extract only the parts containing reads that map to the HPV genome using samtools (v.1.14 [43]). These reads were processed by “samclip” (v.0.4.0 [44]) under default operating parameters to filter out reads that had more than five soft-clipped bases in the given alignment. Afterwards, the iVar computational package (v.1.4.2 [45]) was used to perform variant analysis and generate the consensus sequence of HPV16 from a particular sample. For variant calling, the results of the samtools mpileup command (uncapping the depth, not discarding anomalous read pairs, and considering all bases, including those with no mapped reads) were piped into the iVar consensus command (requiring the depth of bases above a Phred quality score > 20 to be no less than three in each given position and choosing the most frequent base for the consensus sequence; insertions were included only when they had a frequency threshold of at least 80% at a given position). The consensus nucleotide sequences (n = 16) were deposited into the GenBank BioProject PRJNA1291997 with accession numbers PV809647 to PV809662 (Supplementary Table S1).

### 2.6. Phylogenetic Analysis of the Latvian HPV16 Isolates

Sequences serving as the references for the sublineages of the major HPV16 lineages (subgenotypes) A, B, C, and D were downloaded from “The Papillomavirus Episteme” isolate genomes on the 19th of February 2025 [46]. The genome representations were reorganized (rotated) to be collinear with the NC_001526.4 HPV16 reference genome using an in-house script. Afterwards, both the representative HPV16 sublineage references and the consensus sequences of the Latvian isolates were subjected to multiple sequence alignment using MAFFT (v.7.525 [47]) in the standard (FFT-NS-i) mode. The resulting multiple-sequence alignment was used to reconstruct a maximum-likelihood tree with the help of IQ-TREE (v. 2.3.5 [48]), enabling automatic best-fit substitution model selection via ModelFinder [49], allowing polytomies, and evaluating branch supports using 1000 ultrafast bootstrap replicates (UFBoot [50]). The resulting tree was midpoint-rooted and visualized using FigTree (v.1.4.4 [51]).

### 2.7. Analysis of the Variations Identified in the Latvian HPV16 Isolates

Reconstructed consensus sequences of the Latvian HPV16 isolates were concatenated into a multiple sequence fasta file together with the reference sequence and other publicly available complete or near-complete HPV16 isolate genomes (n = 4237), making a total of 4254 sequences. Metadata for the sequences of potential interest were retrieved using the NCBI Datasets CLI (v.15.9.1 [52]), filtered to include only the sequences corresponding to “Alphapapillomavirus 9” and containing either “Human papillomavirus type 16” or “Human papillomavirus 16” in the name, with accessions longer than 7115 bp corresponding to ~90% of the HPV16 reference genome coverage. Respective sequences were subsequently downloaded as linear sequence representations, changing the starting position to be collinear with the reference using an in-house script when necessary. The dataset was then processed against the HPV16 reference genome (NC_001526.4) using NUCmer (v.3.1—[53]) and “show-snps” from the same package. Afterwards, the “coronannotator” code from the Giorgi lab [54] was downloaded, and the function “annotator” was run using the generated snp file alongside the reference genome (NC_001526.4) and its annotation in the general feature format (gff3). Downstream analysis (such as counting frequencies of each variant in the Latvian HPV16 isolates and HPV16 isolates from elsewhere) was performed using the “R” programming language functionality and packages. This larger dataset of 16 Latvian and 4238 complete or near-complete HPV16 genomes collected worldwide was also subjected to maximum-likelihood tree reconstruction as described in Section 2.5. The only modifications were that the “fast” (FFT-NS-2) option was selected during the multiple sequence alignment via MAFFT [55]. Further reconstruction used IQ-TREE multicore version 2.5 [56].

### 2.8. Analysis of the Direction of Selection

Analysis of the direction of selection for DNA encoding HPV oncoprotein E6 was run on the multiple sequence alignments using an online calculator, SNAP v2.11 [57], based on the Nei–Gojobori method [58] and Jukes–Cantor Model [59]. The only sequence changes considered were SNPs without accounting for duplications or inversions. The direction of selection was assessed using a sliding value of the ratio of non-synonymous (dN) and synonymous (dS) substitution rates (dN/dS) across the gene.

### 2.9. Analysis of Co-Variation

A total of 294 HPV16 genome fragments encoding the E6 oncoprotein derived from HPV16 isolated in Europe were downloaded from the GenBank NCBI database (Available online at https://www.ncbi.nlm.nih.gov/nuccore/advanced, accessed on 29 December 2023). Samples containing the complete nucleotide sequence of E6 ORF were selected using the MEGA11 program [60]. Nucleotide sequences, first of *E6* derived from WGS (n = 16), then of 16 *E6* derived from WGS and 31 *E6* derived from the Sanger sequencing (n = 47), and finally of the full-length European HPV16 *E6* extracted from Genbank (n = 294), were translated and aligned using msaClustalW.

Multiple sequence alignments of HPV16 E6 were subjected to covariation analysis/correlated mutation analysis. The latter produces three types of metrics that measure the dependence between any two positions of the protein. Analysis of co-variance was done using an R/Bioconductor package for computing correlated mutations based selection pressure using mutual information (CorMut) [61]. The CorMut tool itself does not allow for defining which values are significantly different from the noise. To determine this, a permutation test was implemented, where the original sequences were shuffled and the analysis was run using the shuffled sequences. The scores obtained in this way were then compared with the actual scores to determine if they were higher than the random “noise”. Analysis of each set of sequences employed 300 permutations. Data was further analysed using the Observed minus Expected Squared (OMES) method (matrix), which calculates the squared difference between each observed value and its corresponding expected value in each data point in the dataset. Results were presented as: (i) raw output in Excel format; (ii) filtered outputs of the correlation analysis (includes significant associations, *p* < 0.05); (iii) network plots of the significant associations. Network graphs created using OMES were plotted by filtering the statistically significant position association scores (*p* < 0.05).

### 2.10. Statistics

Comparison of the frequency of SNPs in HPV16 WGS sequences, and resulting AAS, observed for samples collected in 2012–2019 versus those collected in 2020–2024, and in sequences from CINIII/SCC grade 1 cases versus SCC grade 2/3 cases was done for a set of 12 polymorphisms detected in >30% of the Latvian isolates. Multiple comparisons were done using nonparametric tests for data not following normal distribution, or chi-squared tests, both for small sample sizes. Monotonic relations between the variables were analysed using Spearman’s rank correlation test. Data on the prevalence of single SNPs/AAS in the subsets of HPV16 WGSs was analysed pairwise using the Mann–Whitney U-test, Fisher’s exact nonparametric tests, or chi-squared tests with adjustments for small sample sizes without the control of the Family-Wise Error Rate (FWER) using instead repeated experiments to confirm the results on an independent data set (Table 2) [62,63,64].

Statistical analysis was performed using Statistica 13.5.0.17 (TIBCO Software Inc. Palo Alto, CA, USA) and GraphPad Prism version 9.0.2 (GraphPad Software, San Diego, CA, USA). A statistical significance threshold of 0.05 was used throughout the study. All *p*-values were two-sided.

## 3. Results

### 3.1. Reconstruction of 16 Whole Genome Sequences of the Latvian HPV16 Isolates

**Figure 1:**
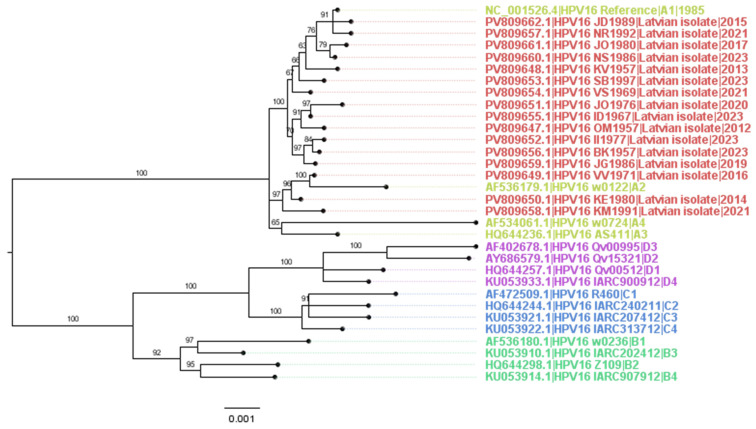
**Midpoint-rooted maximum likelihood tree of the complete HPV16 genomes from Latvia within the context of the recognized HPV16 sublineage reference sequences.** The tree is drawn to scale, and branch lengths correspond to the number of nucleotide substitutions per site. Branches have their ultrafast bootstrap (UFBoot) support percentage (out of 1000 replicates) indicated above them. Tip labels are in the form of “Accession|Isolate designation|Sublineage|Year (if unambiguously known)” and are coloured arbitrarily based on the lineage they represent. North American sequences are coloured in blue, South American in lilac, Asian in green, European in turquoise, African in pistachio, and Latvian sequences in red. The multiple sequence alignment used as input for the generation of the tree was performed using MAFFT in standard (FFT-NS-i) mode and had 32 sequences, 7921 columns, 318 distinct patterns, 192 parsimony-informative sites, 220 singleton sites, and 7509 constant sites. The maximum-likelihood tree was generated using IQ-TREE with K3Pu+F+I chosen as the best-fit model according to BIC after the ModelFinder analysis.

From the WGS data obtained for FFPE samples of women with cervical disease harbouring high loads of HPV16 (Table 1), we could reconstruct 16 high-quality genomes of the local HPV16 variants, hereby and throughout the study referred to as isolates in line with the term used in GenBank submission. All but one (PV809653) of the resulting genomes had an estimated coverage of more than 99.75% and a mean base depth of at least 20×. The wgs_S11819Nr5 could only be assembled to the reference with a coverage of ~93.59% and a mean base depth of 12.4× (Supplementary Table S1). Phylogenetic analysis revealed that sequences of the local HPV16 isolates were non-redundant, showing a degree of variation from each other. However, the evolutionary distances between these Latvian isolates were rather short, with the most distant ones (PV809658 and either PV809657, or PV809662) being only ~0.004 substitutions per site apart. The most similar local isolates were around 0.0005 substitutions per site apart (PV809656 and PV809652; Figure 1).

Within the current framework of the HPV16 (sub)lineage designation, all 16 of the Latvian HPV16 sequences clustered together with the so-called “A” lineage of HPV16. More specifically, 13 isolates were closely related to the “European” sublineage A1, and 3 to the “European” sublineage A2 references (Figure 1).

Analysis within the context of all publicly available HPV16 complete or near-complete genomes revealed that each of the Latvian variants was more similar to some other non-Latvian HPV16 variants than to the other Latvian variants. In a global context, none of the Latvian HPV16 isolates was found to share the most recent common ancestor with another isolate from Latvia (Supplementary Figure S1 and Supplementary Dataset S1), indicating that, in the evolutionary sense, HPV16 viruses circulating in Latvia were not closely related to each other.

### 3.2. Analysis of HPV16 Isolates

A total of 93 non-redundant variants were identified across the Latvian HPV16 whole genome sequences. These included 30 extragenic variants and 63 variants located within the protein-coding regions of the HPV16 genome. Out of these 63 single-nucleotide polymorphisms (SNP), 30 were silent and 33 were missense, leading to single amino acid substitutions (AAS). The missense variants identifiable in the Latvian samples affected mostly the minor capsid protein L2 (9 of 33 SNPs; 27%) and less frequently the early proteins E2 (8 of 33 SNPs; 24%), E1 (5/33; 15%), E6 (4/33; 12%), E5 (3/33; 9%) and E1^E4 (one deletion) (Table 3). The gene encoding the major capsid protein L1 harboured only three such variants (3/33; 9%). Interestingly, the frequency of SNPs leading to AAS in L1 was not higher than that in L2 (on the contrary, it tended to be lower, *p* = 0.0589, chi-squared test), indicating an absence of enhanced immune pressure on L1 compared to L2.

Four amino acid sequence-altering SNPs unique to the Latvian HPV16 isolates were found in the genes encoding early proteins, namely (1) E1: A161C resulting in N54T; (2) E1: G239T resulting in R80I; (3) E1: A350G resulting in K117R substitutions; and (4) E2: A2978C changing the amino acid residue in position 363, resulting in M363L (Table 3). Interestingly, a single in-frame deletion of 24 nucleotides was found in the protein-coding regions of the genome from position 2707, affecting both E2 and E1^E4 proteins. The deletion resulted in the loss of 8 amino acid residues starting from the 273rd and 72nd positions of E2 and E1^E4, respectively (Table 3). The aforementioned deletion was not found in the completely sequenced HPV16 isolates from elsewhere. Major capsid protein L2 was affected by a single unique nonsynonymous SNP G3499T, resulting in L2: D43Y (Table 3).

### 3.3. Genomes of the Latvian HPV16 Isolates Demonstrate an Absence of Co-Variance Between Single Positions

Next, using the Bioconductor package, we attempted to compute correlated SNPs within the Latvian HPV16 genomes using information on the selection pressure (mutual information; CorMut) [61]. Non-zero results were retrieved for the base pair residues (bp) belonging to the E1 ORF, namely 862 and 1101 (R = 0.152510403), 862 and 1700 (R = 0.0098), and 1101 and 1700 (R = 0.045). However, none of the co-variances was statistically significant (*p* > 0.5).

### 3.4. Analysis of SNPs Leading to Amino Acid Substitutions Along Open Reading Frames of the Early HPV16 Proteins

We next analysed whether there were differences in the frequency of occurrence of AAS in HPV16 ORFs between cases of CINI-III and CSCC, indicating their association with disease severity, and also eventual changes of the frequencies over time. As the tests were exploratory, we chose to make no adjustments for multiple testing based on the rationale of non-adjustment as the default starting position in simple multi-arm trials comparing distinct parameters—in this case, distinct sequence features. We were well aware that the danger of interpreting a result as definitive and ‘confirmatory’ if only one parameter is significant among many tested must be minimized, necessitating control of the Family-Wise Error Rate (FWER) through multiple comparison corrections. However, as noted by Howard D.R. et al. [63] and by Parker RA and Weir SJ [62], instead of multiple testing, this problem can be addressed and resolved by replication and repeated experiments confirming the results. Based on this, the decision was made to confirm differences, if observed, in independent patient cohorts/datasets.

*Early protein E1* In the E1 ORF, we observed 9 synonymous and 7 nonsynonymous SNPs in E1, including E1:N54T, E1:E63D, E1:R80I, E1:K117R, E1:R125R, and E1:S220T (Table 3; Supplementary Table S2). The most common was E1:S220T, detected in 5/16 (31%) of the Latvian HPV16 genomes, similar to the 33% seen in the full-length HPV16 genomes collected worldwide (n = 4237; Table 3).

*Early protein E2* In the E2 ORF, we observed a total of 4 synonymous and 8 nonsynonymous SNPs (Table 3, Supplementary Table S2). Interestingly, the majority of the isolates carried one synonymous and up to three nonsynonymous SNPs. The most common was the SNP leading to P219S (10/16; 62.5%). We also detected a combination of I210T, P219S, and T310K (3/16; 18.8%), but never I210T or T310K alone. The combination of E2: I210T/P219S/T310K was seen in three cases, of which two were CINIII, and one was CSCC of grade 2; i.e., in the Latvian cohort, the combination of E2: I210T/P219S/T310K was not associated with a more severe cervical disease. One of these “I210T/P219S/T310K” isolates, namely the isolate from pt BBH71_15 (PV809649), also had a unique deletion of the region coding for 8 aa residues in E2 (Table 1 and Table 3; Supplementary Table S2).

Overall, the prevalence of SNPs detected in E1 and E2 was the same among HPV16 isolates collected in 2012–2019 and in 2020–2024, as well as among isolates from CINIII/G1 and from G2/G cases; i.e., it was not associated with either the time of sampling or the severity of cervical disease (all *p* values > 0.1) (Table 4).

*Early protein* E1^E4 The E1^E4 ORF was characterized by a series of synonymous SNPs along with the deletion of 8 aa residues in the HPV16 isolate from pt BBH71_15 (PV809649) linked to the deletion in E1 (Table 1, Supplementary Table S2).

*Early protein* E5 In the E5 ORF of the Latvian HPV16 isolates, we observed three nonsynonymous and one synonymous SNP (Table 3, Supplementary Table S2). The main polymorphisms leading to AAS were E5: I44L (8/16, 50%) and E5: I65V (9/16; 56%), which were paired in most of the cases (Table 3). Only one HPV16 isolate had a single E5: I65V. The previously described AAS at position 48 was rare (one isolate had E5: L48V together with E5: I44L and E5: I65V). The prevalence of E5: I65V remained stable in the viral population, with no difference between samples collected before and after 2019 (Table 4). AAS I44L and I65V in E5 were not related to disease severity, as the same prevalence was seen in CINIII/G1 and G2/G cases (all *p* values > 0.1; Table 4).

*E6 and E7 oncoproteins* In the E6 and E7 ORFs of the Latvian HPV16 isolates, we observed a total of 12 SNPs at four sites (Table 4; Supplementary Table S2). The E7 gene was highly conserved, with only one synonymous SNP observed. In the E6 gene, SNPs A7173G, G7174C, G7230C, and T7392G encoded amino acid substitutions R17T, R17G, E36Q, and L90V, respectively (Table 3 and Table 4). The most prevalent was the aa substitution L90V, detected in 43.75% of the cases (Table 3). In the Latvian isolates dated before 2020, E6:L90V was present in 28.6%, and from 2020 on, in 55.6% of the sequences (2/7 and 5/9, respectively), but the observed increase in prevalence was not significant. Interestingly, E6: L90V was observed in only one HPV16 isolate from a patient with CINIII (1/6, 16.7%), but in 6 isolates from patients with SCC grades 2 and 3 (6/10, 60%; Table 4).

### 3.5. Analysis of SNPs Leading to Amino Acid Substitutions in ORFs of the Late HPV16 Proteins

*Capsid protein L1* In the L1 ORF of the Latvian isolates, we observed SNPs at 12 sites (3 nonsynonymous and 9 synonymous) (Supplementary Table S2). The main ones were L1 polymorphisms, leading to aa substitution A266T, detected in 10/16 (62.5%) isolates (Table 4). The prevalence of L1:A266T remained stable in the viral population, with no difference between samples collected from 2012 to 2019 and from 2020 to 2024. The distribution of the aa substitution L1: A266T was not related to disease severity, as the same prevalence was seen in CINIII/G1 and in G2/G cases (all *p* values > 0.1) (Table 4).

*Capsid protein L2* In the L2 ORF, we observed SNPs at 14 sites, with 8 being nonsynonymous and 4 synonymous (Supplementary Table S2). The main were SNP at L2 codon 234, detected in 87.5% (14/16) and AAS L2: L330F, detected in 75% (12/16) of the Latvian HPV16 isolates (mostly paired). Other SNPs and AAS within L2 were detected only in single HPV16 isolates. The SNPs at codons 234 and 330 (L2: L330F) were detected in all SCC G2/3 cases, and in all CIN and SCC cases collected after 2019, but statistically, the prevalence of either of them did not change over time, and was not associated with the severity of cervical disease (*p* > 0.1; Table 4).

### 3.6. In-Depth Analysis of the Variability in HPV16 E6 Oncoprotein

E6: L90V was previously associated with persistent HPV16 infection and the transformation of cervical lesions from LSIL to HSIL and further to CSCC (not adenocarcinoma) [65,66,67], irrespective of the geographical origins of the samples/isolates. Analysing WGS of 16 Latvian HPV16 isolates, we observed an insignificant increase in the prevalence of AAS E6: L90V in more severe cervical disease (*p* = 0.1 Fisher test; Table 4). To prove the significance of this observation, we performed replication/repeated experiments recommended by Parker RA and Weir SJ [62] and Howard D.R. et al. [63]. Namely, we performed testing for the prevalence of AAS E6: L90V in CINIII/SCC grade 1 compared to SCC grade 2/3 cases in a retrospective cohort including samples of 31 patients with cervical disease of varying grades observed in Riga, Latvia, during 2016–2024 (n = 31; Table 2).

#### 3.6.1. Amino Acid Substitution L90V in E6 Is Associated with the Severity of Cervical Disease

DNAs extracted from the cervical tissues of 31 women with CINI-III to SCC grades 1–3 were subjected to Sanger sequencing, and the sequences were deposited in GenBank (Table 2; BioProject PRJNA1293798). E6: L90V was detected in none of the CINI-III cases (0/8), in one case of CIN3/cancer in situ characterized by high invasion of crypts and peritumoral lymphocyte infiltration, in none of the SCC grade I cases (0/1) and in 36.4% of the SCC grade 2/3 cases (8/22) (*p* = 0.05 Fisher test; Table 5). The nearly significant association of E6: L90V with the diagnosis prompted us to perform an analysis of the pooled set of HPV16 E6 sequences obtained by WGS (n = 16) and by Sanger sequencing (n = 31), totalling 47 sequences. This analysis proved that AAS E6: L90V observed in the Latvian HPV16 isolates was associated with the severity of cervical lesions, as it was significantly more frequent in SCC grade 2/3 cases than in the less severe cervical disease (Table 5; statistical analysis according to [64,68]).

#### 3.6.2. Analysis of Polymorphisms in HPV16 E6

E6 is a primary oncoprotein that, along with E7, drives the development and progression of cervical cancer and other hrHPV-related malignancies. E6 contains a number of variable/polymorphic aa positions. In the Latvian HPV16 isolates, we infrequently observed R17T, R17G, and E36Q, and in >40% of sequences detected L90V (Table 3 and Table 4). To understand/clarify the association of E6: L90V with the severity of cervical disease, we analysed the data on the role of all polymorphic amino acid residues within oncoprotein E6, including L90V, in the protein structure, biological functions, and recognition by the immune system of the host.

**Figure 2:**
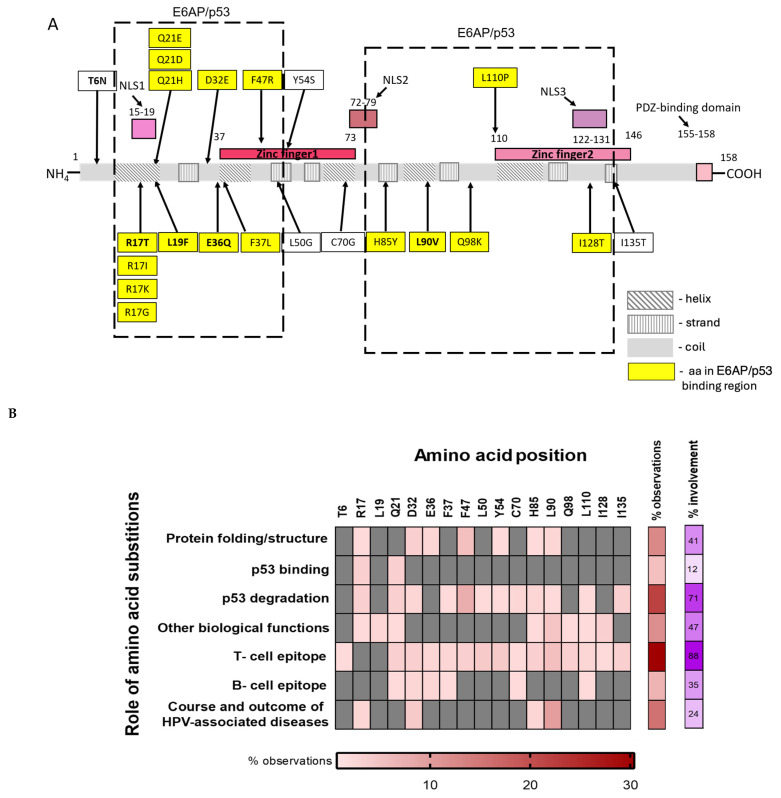

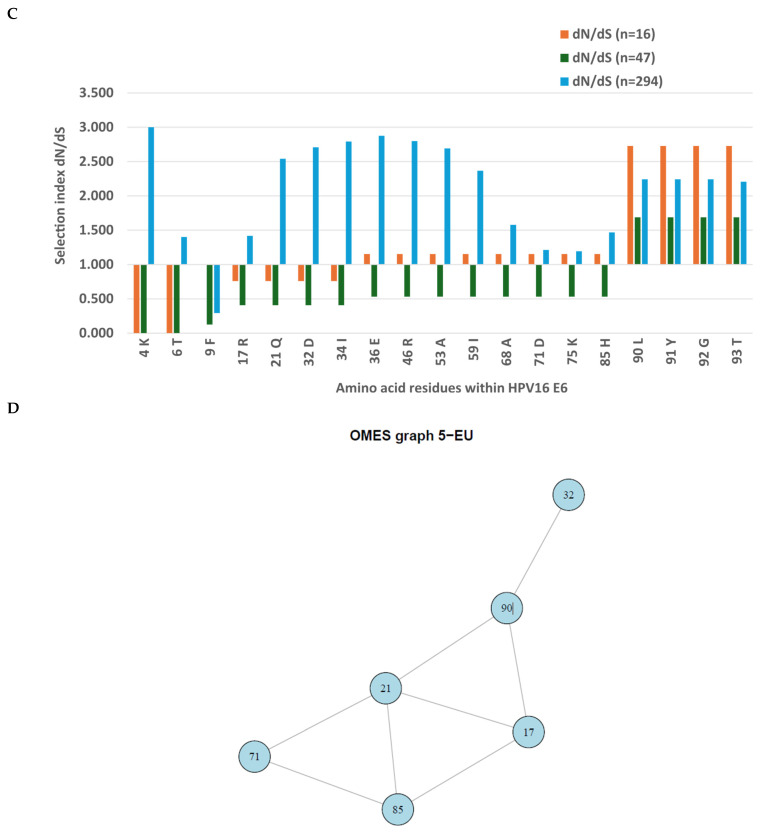
**Structural and functional elements within HPV16 oncoprotein E6 subjected to variation, with the Latvian compared to European HPV16 E6 sequences.** (**A**) Schematic representation of the structural and functional domains including elements of secondary structure such as alpha helixes, strands and coils, as well as functional motifs including nuclear localization signals (NLS) 1–2, a PDZ-binding motif, and two zinc finger domains (adapted from [118]). Boxes with arrows indicate positions of the described variable amino acid residues within E6; AAS identified in this study are highlighted in bold. (**B**) Heatmap summarizing the involvement of AAS in E6 in the protein structure/folding and biological functions, including p53 binding and targeting of p53 for proteasomal degradation, E6 recognition by T and B cells and the effect of given AAS on the course and outcome of HPV16-associated cervical disease (based on studies summarized in Supplementary Table S3); % involvement of polymorphic aa positions in each function (of 17) and % observations of the involvement of an AAS in the given property of the total 143 observations (Supplementary Table S3) are presented as vertical bars on the right side of the panel; (**C**) Direction of selection within the amino acid sequence of HPV16 E6 assessed by SNAP analysis of the aligned amino acid sequences of E6 from WGS of 16 Latvian HPV16 isolates, 16 HPV16 isolates together with E6 sequences of the Latvian HPV16 isolates obtained in a retrospective study (n = 31) (a total of 47), and 294 sequences of the European HPV16 isolates; the X axis indicates positions of amino acid residues, and the Y axis represents the sliding dN/dS ratio (based on the dataset in Supplementary Table S4); positive/diversifying selection is characterized by dN/ds values > 1, and negative selection by dN/dS values < 1. (**D**) Co-variance network for E6 of HPV16 built using OMES-driven analysis of 294 aligned sequences of the European HPV16 isolates.

The localization of polymorphic aa residues within E6 was visualized in relation to the known structural and functional domains of E6 (Figure 2A) basing visualization on the published data summarized in Supplementary Table S3 (in total, 54 references [67,69,70,71,72,73,74,75,76,77,78,79,80,81,82,83,84,85,86,87,88,89,90,91,92,93,94,95,96,97,98,99,100,101,102,103,104,105,106,107,108,109,110,111,112,113,114,115,116,117]). Of the 17 variable amino acid residues in E6, most were found to lie within the domains of E6 that are important for its structure and functions. An interesting observation was that of the 17 polymorphic positions, 11 (64.7%) were shown to be involved in the targeting of p53 to proteasomal degradation (Figure 2B; Supplementary Table S3). Nearly half of the polymorphic positions (7/17, 41.2%) were shown to be involved in other biological functions of E6, specifically in the formation of E6 dimers and nuclear localization (Figure 2B; Supplementary Table S3).

Another important observation was that the majority of the polymorphic positions were localized within the regions of the protein predicted to have an alpha-helical structure harbouring T-cell epitopes (Figure 2B; Supplementary Table S3). Co-localization with T-cell epitopes was shown for 15 of 17 polymorphic positions of E6 (88%; Figure 2B; Supplementary Table S3).

This analysis delineated two forces behind variability in E6, namely a drive to enhance p53 degradation, crucial for the establishment of chronic viral infection and the realization of the oncogenic potential of HPV16, and epitopic drift allowing viral escape from the cellular immune response of the host. If true, their cumulative action would result in positive selection in the evolution of E6. To verify this concept, we assessed the direction of viral evolution in E6.

#### 3.6.3. Analysis of the Variability in HPV16 E6—Direction of Selection

The direction of evolution was assessed as the ratio of nonsynonymous to synonymous nucleotide changes (dN/dS) by running analysis on the multiple sequence alignments. The need to preserve protein structure and/or functions restricts the changes and is reflected by dN/dS values from 0.5 to 1.0, interpreted as negative to neutral, restricting the changes, while dN/dS values > 1 are interpreted as positive, promoting the changes. E6 gene sequences taken into the analysis were not too divergent, and observed substitutions were dispersed in time, providing a sufficient number of changes to draw conclusions [59].

We observed a dN/dS ratio > 1 starting from aa residue 36 of E6 of the Latvian HPV16 sequences (n = 16) and from aa residue 90 of the pooled set of Latvian E6 sequences (n = 47; Figure 2A,B). Analysis of a large set of European HPV16 E6 sequences demonstrated dN/dS ratio > 1 starting from aa residue 17 with a “peak” of positive selection for the region encoding aa residues 21 to 68, another peak after aa 90, and negative to neutral selection for the region encoding aa residues 9 to 17 at the N-terminus of E6 (Figure 2C). All three sets consistently demonstrated strong positive (diversifying) selection for the amino acid residues starting from position 90 (Figure 2C; Supplementary Figure S2, panels A, B, C; Supplementary Table S4), indicating that diversification in the region 21 to 90 of E6, specifically at position 90, confers an evolutionary win to HPV16.

#### 3.6.4. Co-Variance of Amino Acid Residues in Polymorphic Positions of E6

Next, we inquired whether there is any connection between the changes of aa residues in the polymorphic positions of E6, i.e., if these changes occur independently of each other or are associated. For this, we analysed E6 sequences for the presence of co-variance networks. Co-variance networks for the sets of 16 and 47 E6 sequences could not be built due to low sequence variation. However, such networks could be generated for the set of 294 full-length HPV16 E6 sequences from Europe (Figure 2D). Interestingly, the co-variance network generated united the main polymorphic aa positions in E6, namely 17, 21, 32, 71, 85 and 90, which we have shown above to form the dN/dS ratio “break-points” reflecting increasing positive selection (Figure 2C).

The fact that polymorphic positions in E6 are united in a co-variance network indicates an orchestrated evolution of HPV16 E6 towards better realisation of certain biological functions/properties, suggestively the degradation of p53, alongside with an escape from the T-cell immune response. Associations with the persistent HPV16 infection and transformation of cervical lesions from LSIL to HSIL and further to SCC, reported for other polymorphic aa residues in E6, namely 17, 32, and 85 (Figure 2B; Supplementary Table S4), could have the same explanations as those given above for E6: L90V.

## 4. Discussion

Taxonomically, HPV16 is a member of the Alphapapillomavirus 9 species within the Alphapapillomavirus genus of the Papillomaviridae family [119]. HPV16 features a small circular double-stranded DNA genome of around 7.9 kbp in length [120], which can be integrated into the chromosome of the host [121]. The genome of HPV16 encodes several early non-structural proteins involved in viral replication (E1 to E7), as well as two late structural proteins (major capsid protein L1 and minor capsid protein L2), encoded by an 8 kb-long double-stranded DNA and expressed through alternative splicing [122]. The genomic variability of HPV16 has been extensively studied in North America and in Western Europe, including Germany, France, the UK, the Netherlands, Sweden, and Finland; large topical studies were carried out in Latin America, Asia, and South Africa [123]. At the same time, data from Eastern European countries, specifically the Baltic region, including Latvia, is limited. By analysing the genomes of HPV16 isolates from cervical neoplasia and squamous cervical carcinoma cases in Latvian women, we aimed to fill this knowledge gap.

Analysis of the complete genomes of HPV16 circulating in Latvia from 2012 to 2023 demonstrated that all HPV16 isolates belong to the European A HPV16 lineage. We observed a degree of diversity among HPV16 isolates that is considerable for dsDNA viruses known for their slow mutation rates, demonstrating a highly non-uniform genetic background of HPV16 isolates circulating in the Baltic region, particularly in Latvia. Latvian HPV16 isolates demonstrated high pairwise similarity, as well as unique mutations. Although we found several globally rare or even unique protein-altering SNPs, most of the genetic variants identified in the local HPV16 isolates have been previously recorded in the context of HPV16 from other regions of the world.

In a way, Latvian HPV16 isolates were more similar to HPV16 isolates from distant regions of the world than to other Latvian isolates, indicating that the existence of a local “Latvian” sublineage or isolated strain (variants of a strain) is not an issue. This is consistent with historical accounts of the local population(s) not being isolated at any point in recorded history to allow such a lineage to emerge. Close contact with other population groups, broadly expedited in the current era of globalization, undoubtedly results in the exchange of HPV isolates, diversifying the repertoire of locally circulating viral variants. To conclude, our HPV16 data confirmed a high degree of global hrHPV homogenization, with the Latvian HPV16 isolates forming a part of the global pool of HPV16 isolates, reflecting both human mobility and high levels of virus transmission in the general population.

This study gave us an opportunity to identify HPV16 isolates capable of HPV vaccine escape and contributed to a better understanding of the global evolution of HPV16 in the context of longitudinal mass HPV vaccination. HPV vaccines such as Cervarix, Gardasil, and Gardasil 9 use a subunit technology based on the major capsid protein L1 [124]. L1 protein is essential for assembling the viral shell, protecting the genome, and mediating initial infection by binding to the cell surface [125]. Assessment of the diversity of HPV16 L1 sequences allocates it to the antigenic regions (mainly), which is of great importance for the efficacy of prophylactic vaccines, as it allows for the evaluation of the impact of genetic variations in these regions on vaccination-induced immunity. El Aliani A. et al. assessed a total of 377 HPV16 L1 sequences, published in the public domain GenBank database, from the Americas, Africa, Asia, and Europe. A total of 626 mutation events were reported affecting 83 distinct codons of the HPV16 L1 gene encoding five antigenic regions, mainly the DE (27.38%, 23/83) and FG (31%, 26/83) loops. The most frequent were AAS T176N and N181T in the EF, T353P/I/N in the HI, and A266T in the FG loops [126], the latter present in the majority (>70%) of the Latvian HPV16 isolates. L1: A266T was claimed to play an important role in the recognition of the viral capsid by neutralizing antibodies: binding of anti-L1 Mab to the A266T variant of L1 was reduced by almost half in comparison to the wild-type L1 [127]. L1: A266T was also suggested to alter the binding of antibodies generated by vaccines, potentially allowing the virus to evade the immune system’s neutralizing effects. Being one of the most common substitutions in L1 across the globe [128], L1: A266T has shown distinct differences in geographical distribution, attributing its emergence to the immune response [129]. However, later studies revealed that the immune response was not due to HPV vaccination. A comparative study of HPV16 genome variability in HPV vaccinated versus nonvaccinated women by van Eer K. et al. demonstrated that L1: T266A was common and present in similar frequencies among viruses isolated from vaccinated and unvaccinated women, indicating a random effect of this substitution on viral circulation [130]. Furthermore, no links have so far been established between the L1: A266T substitution and the progression rate to cervical cancer, indicating no failure in immune protection. In our study of the Latvian cohort of HPV non-vaccinated women, the representation of L1: T266A was not associated with disease severity and did not change over time, confirming the concept proposed by van Eer K. et al. regarding the random effect of L1: T266A on viral circulation [130]. Altogether, this indicated that L1:T266A in the Latvian HPV16 variants was not a mutation of immune escape from HPV vaccination.

L2 is essential for the viral life cycle, as it facilitates the transport of the viral genome to the nucleus, nuclear import, and capsid assembly [131]. In the L2 gene, we observed 6 SNPs leading to 5 AAS detected in single HPV16 isolates. Additionally, in the majority of the Latvian HPV16 isolates, we observed missense SNPs in codon 234 of *L2*, with AAS L2: L330F being mostly paired. A high prevalence of L2: L330F was noted in Europe, Asia and North America [132]. The biological relevance of this AAS is unclear. The high-frequency variation of L2: L330F (and also L2: S269P) was suggested to be driven by immune pressure [132]. Debernardi A. et al. demonstrated that for HPV16 of lineages A1 and A2 (Europe), L2:L330F was significantly more frequent among women with CIN self-clearing HPV infection compared to those developing chronic disease [133]. Our data do not support these findings. We noted a wide distribution of L2: L330F in our HPV16 lineage A isolates (over 70%) in both cases of more and less severe cervical disease. Of note, we detected L2: L330F in HPV16 isolates from three patients clearing HPV16 infection (NR1992, KM1991, and JG1986, Table 1), although no conclusions on the relation between L2: L330F and HPV16 clearance could be made as our study was not designed to dissect the differences in the occurrence of SNPs and AAS in patients clearing and not clearing HPV16 infection. Overall, according to our data, AAS L2: L330F in HPV16 lineage A was not associated with either viral persistence or severity of cervical disease.

There is an additional angle to the data on the SNPs and AAS in HPV16 L2. L2, which is not included in the HPV vaccine, was affected by eight AAS (nine SNPs), including the widely distributed L2: V243I, L2: S269P, L2: L330F and L2: E338K, while L1, which is included in the HPV vaccine, was affected by only three AAS, including a widely distributed AAS L1: T266A (Table 3; Supplementary Table S3). In this context, the frequency of AAS in the “non-vaccine” L2 tended to be higher than in L1 (although insignificantly), but not the other way round. Furthermore, we have shown that the prevalence of SNPs and resulting AAS in L1 detected after the onset of uniform Gardasil-9 vaccination in 2020 was not higher than in 2012–2019 when first Cervarix and then Gardasil were implemented in girls.

Altogether, these data support the statement above regarding the lack of relationship between AAS L1:T266A and HPV vaccination, and show that HPV16 isolates circulating in Latvia among non-HPV vaccinated women exhibit no signs of increased variability in L1, which would point at immune escape or attempts at immune escape in response to mass HPV vaccination. 

This study was also planned to uncover the molecular characteristics of HPV16 isolates circulating in Latvia, to associate HPV16 variants and particular SNPs and resulting AAS with the clinical manifestations of HPV-associated cervical disease, similarly to earlier studies done for hr HPVs, and for HPV16 in particular (see, for example, [46,134]). As we identified none such in the genes encoding capsid proteins L1 and L2, the analysis focused on the genes encoding early HPV16 proteins.

Several common and unique SNPs and AAS were detected in the genes encoding early HPV16 proteins E1, E2, E1^E4, E5, E6 and E7. The E1 gene encodes a DNA helicase that initiates and drives viral genome replication by unwinding the DNA working in a ternary complex with E2 at the viral origin of replication [135]. As an ATPase, E1 forms double hexamers, recruits cellular DNA polymerase complexes, and is critical for the amplification stage of the HPV life cycle [135,136]. Sequence variations in E1 enable the classification of HPV16 into four major phylogenetic branches [137]. A number of SNPs and single AAS are increasingly recognized for their potential role in, and correlation with, cervical cancer progression, although associations differ depending on the geographical origins of the samples, i.e., the ethnicity of the patients. An extensive sequence analysis of the HPV16 E1 gene revealed a multitude of sequence variations in the N-terminal, DNA-binding and C-terminal domains of E1 typical to the cervical cancer cases in the Chinese population [138], whereas in Greek patients these changes were associated with HSIL [139]. E1 variants with aa substitutions in the C-terminal domain were detected only in the European HPV16 isolates and only in association with HSIL cases [137]. For the Latvian HPV16 isolates, the most common substitution was aa E1: S220T, which in the Latvian patients was not associated with the severity of cervical disease.

HPV16 E2 protein is likewise essential for the replication of viral DNA. E2 binds to DNA sequences within the viral long control region (LCR) and acts as a chaperone, recruiting E1 helicase to initiate viral replication [135]. E2 also facilitates genome partitioning during mitosis [140]. AAS E2: P219S in the transactivation domain at the N-terminus of E2 and E2:T310K at the DNA-Binding Domain at the C-terminus have been previously associated with a higher risk of developing CC, suggestively because they may hamper or eliminate the function of E2 protein as the transcription suppressor for the E6 and E7 genes, driving malignant transformation [139]. However, Giannoudis A. et al., studying 22 HPV16-positive LSIL and 43 HPV16-positive HSIL cases, could not confirm that either E2: P219S or E2: T310K correlated with high-grade lesions [141]. Also, a large-scale study of SNPs in HPV16 associated with oropharyngeal cancer prognosis did not reveal any disease-related SNPs in E2 (only in E1, L1, L2 and in the upstream regulatory region/URR; [142]). For the Latvian HPV16 isolates as well, possession of E2: P219S alone or in combination with E2: I210T/P219S/T310K was not associated with disease severity.

Interestingly, one of the isolates with AAS in E2, namely PV809649, carrying E2: 210T/P219S/T310K, had a disruption of the E2 sequence. Such disruptions are intriguing in the light of the hypothesis that they may play a role in the induction of chromosome abnormalities in the host characteristic of squamous cervical lesions [143]. The connection is not direct, but is mediated through the disruption-induced loss of E2 control over the expression of E6 and E7, which in turn induces genomic instability [144]. In our case, corroborating the above observations, the disruption was observed in an HPV16 isolate derived from a patient with SCC grade 2. One should, however, keep in mind that genomic disruptions, also in the viral genome, are significantly more common in invasive carcinomas than in less severe lesions [145], being a consequence rather than the cause of chronic hrHPV infection. This does not compromise the potential predictive value of HPV (and host) genomic disruptions, although we could not check this in our cohort.

The HPV16 E1^E4 protein is a highly expressed late protein that acts during the productive phase of the HPV infection cycle to facilitate virus release, mediate cellular damage, and maintain a favourable environment for viral replication [146]. Latvian HPV16 isolates have five SNPs in *E1*^*E4*, none resulting in AAS and a deletion of 8 aa residues after position 78 (E1^E4:A79-T86) in one HPV16 isolate. It was previously shown that a C-terminal deletion mutant of E1^E4 (16E1^E4Δ87-92) cannot form multimeric structures and act as a keratin cross-linker, facilitating the tethering of E1^E4 binding proteins in the cytoplasm [147]. The consequences of the deletion of aa residues 79–86 preceding this region on the structure and functions of E1^E4 are yet unknown. Overall, SNPs/AAS in E1^E4 of the Latvian HPV16 isolates were rare, and were not associated with disease severity.

The HPV16 E5 protein is a transmembrane protein acting primarily in the early stages of viral infection to promote a favourable environment for viral replication. E5 is also important for the productive stage of HR HPV infection [148]. The aspects of transmembrane protein–protein interactions, cellular signal transduction, and cell biology promoted by E5 make it a prominent viral oncoprotein [149]. For E5, AAS in position 65 in different HPV16 subgenotypes/lineages have been associated with viral persistence and increased viral aggressiveness [139]. Reverse genetics demonstrated that the introduction of AAS E5: I44L and E5: I65V into the HPV-16 E5 reference backbone results in an increased ability of E5 to induce mitogenesis of immortal murine fibroblasts through a reduction of the level of p21 expression [150]. The effect of E5: I44L/I65V on E5 activity in the fibroblasts as well as positive selection at codon 65 in *E5* [151] indicate the involvement of this AAS in viral replication and potential input into chronicity [151]. We could not confirm this hypothesis based on data from the prospective Latvian cohort, possibly due to the small sample size.

In HPV16-driven oncogenesis, the key role is played by oncoprotein E6 [139]. We observed three AAS in E6 affecting positions 17, 36 and 90. The crucial one was AAS E6: L90. Most researchers agree on the critical role of E6: L90V in pathogenesis, namely, its association with HPV16 persistence culminating in cervical cancer all over the world. In Saudi Arabian population, AAS E6: L83V (L90V) was associated with the highest increased risk of cervical cancer (OR: 10.1; 95% confidence interval (CI): 1.1–89.4) [152]. In India, E6: L83V (L90V) substitution was observed in 76.2% of HPV16 sequences derived from cervical cancer cases [153]. In the Japanese population, the E6: L83V (L90V) variant showed a higher risk for SCC, though not reaching the level of significance (RR = 3.0, 95% CI = 0.9–10.5, *p* = 0.08) [66]. In Argentina, E6: L90V was associated with progression to HSIL/CC with an OR of 19.41 (4.95–76.10) [154]. In a Swedish study, the HPV16 E6 variant L83V (L90V) was present in 40% of CIN lesions, 81% of the invasive squamous carcinomas, and 54% of the invasive adenocarcinomas [109]. Likewise, in Latvia, we found AAS E6: L90V to be significantly more frequent in squamous cell carcinomas grade 2/3 than in less severe disease cases.

To understand the mechanism behind this association, we analysed available information on the role of the amino acid residue in polymorphic position 90 in the structure and functions of E6. A major role in viral evolution is played by the anti-viral immune response of the host [155], driving immune escape. Interestingly, E6: L90V was shown to change the binding affinity of the substitution-carrying peptides to a number of MHC class I alleles [85,114] (see Supplementary Table S3 and references therein). As a result, women harbouring alleles HLA-B*44, HLA-B*51, or HLA-B*57 that do not recognize the V90 variant exhibited an increased risk of developing cancer compared with the controls [114]. Matsumoto K. et al. investigated the association between E6 variants and human leukocyte antigen (HLA) polymorphisms within the Japanese population [73]. Fifty-seven women with HPV16-positive cervical cancer were analysed for E6 sequence variation and its relationship to HLA class II alleles. Compared with local controls and published controls, DRB1*1501 and DQB1*0602 frequencies were significantly increased among patients with the HPV16 E6 prototype, i.e., patients with these HLAII alleles were unable to mount a clearing immune response against E6. DRB1*1502 was positively associated with a particular E6 variant designated D25E (DRB1*1502 could not recognize the E6: D25E variant). No significant association was, however, found between HLA class II alleles and L90V variants, indicating that L90V is not associated with immune escape from CD4+ T-cell or B-cell immune response, at least in the Japanese population [73]. Li T. et al., focusing on a series of aa substitutions in E6, namely H31Y, D32N, D32E, I34M, L35V, E36Q, L45P, N65S and K75T, which have been shown to affect recognition of B-cell epitopes, confirmed an absence of positive selection in E6 by the humoral immune response [67]. The latter indicated that host-related genetic cofactors are HLA I allele(s) determining the inability of CD8+ T cells to recognize the E6: L90V variant, a specific match (or rather, mismatch) between E6 variants and HLA types contributing to immune escape leading to persistent infection and development of cervical cancer in certain populations. Overall, this aligns with the key role of CD8+ T cells in the control of HPV16-associated carcinogenesis [156,157].

At the same time, the “universally” negative role of E6: L90V across the globe argues against the decisive role of the anti-E6 CTL response, as the pressure of CTLs differs among population groups depending on the prevalent MHC class I alleles, which results in differential recognition of E6 epitopes in different geographic regions [158]. The universal nature of E6: L90V indicates the involvement of factors unrelated to immune response/immune escape. Analysing the literature, we found that the AAS E6: L90V of HPV16 (L83V in specific nomenclatures) enhances the capacity of E6 to degrade p53 compared to the prototype (reference) E6 sequence [116,159] (Supplementary Table S3), a process unrelated to the HLA background of the population. The high global prevalence of E6: L90V and its association with progression to cervical cancer, despite the differential recognition of E6: L90V in different population groups, points to the crucial role of the capacity of mutated E6 (E6-L90V) for p53 degradation in the global establishment of this AAS. This capacity can eventually complement E6-L90V-mediated escape from the T-cell immune response. Each of these factors alone, and their combination, can act as drivers of positive selection in the evolution of E6.

We further analysed E6 for signs of positive selection that would support this concept. An earlier study registered positive selection for codons 17, 21, 34 and 90 within the E6 oncogene [158], which was later confirmed for E6 codons 17, 21 and 90 [151]. However, Li T. et al. detected no positive selection at these or any other sites of E6 [67]. Our study of the Latvian HPV16 isolates confirms positive selection for nonsynonymous substitutions in *E6* for codon 90, but not for codons 17, 21, or 34. Interestingly, however, positive selection targeting region aa 21 to 68 and at aa 90 of HPV16 E6 was revealed for a broader set of 294 European full-length HPV16 subgenotype A sequences, possibly reflecting immune pressure on the virus of broader specificity than that exhibited in the Latvian population.

Viral evolution is realized through positive selection and co-variance of amino acid residues within antigenic regions. These mechanisms shape the disease severity by allowing to enhance viral replication rate and permitting immune evasion. They also mediate a balance between transmissibility and virulence across host populations, as has been explicitly shown for SARS-CoV-2 [160]. In this study, we have shown that amino acid residues at positions 17, 21, 32, 71, 85 and 90 of HPV16 E6 are not just variable, but form a statistically significant co-variance network, i.e., co-vary concordantly. In the context of viral evolution, the interconnectedness of the variable amino acid residues within HPV16 E6 on the background of positive selection, points to coordinated evolutionary changes benefiting viral replication/persistence. At least some of the aforementioned aa positions are involved in enhanced p53 binding and degradation (Figure 2B, Supplementary Table S3), releasing the brakes on viral replication and promoting the survival of HPV-infected premalignant and malignant cells. Thus, the observed co-variance may reflect a coordinated way to compensate for unfavourable losses in p53 binding/p53 degradation due to immune escape, which optimizes the capacity of the virus to establish chronic infection and, consequently, increase the severity of the associated disease.

## 5. Conclusions

The genetic background of the Latvian HPV16 isolates associated with cervical cancer is non-homogeneous, despite the fact that all HPV16 isolates in the study originate from the “European” A lineage. Rare and unique mutations found in association with the local isolates warrant further investigation into the diversity of HPV16 circulating in Latvia. Of a series of common and unique AAS identified in the Latvian HPV16 isolates, only one, namely E6: L90V, was found to be associated with the severity of cervical disease. We found E6: L90V to be under positive evolutionary selection, co-varying with amino acid residues in other polymorphic positions of E6. Analysis of the role of the polymorphisms in E6 in terms of structure, biological functions and immune recognition pointed to the main driving forces behind this evolution: immune escape from cytotoxic T-cell response alongside with a strive for a more efficient degradation of p53. This study was small-scale. Intensification of HPV16 surveillance with wider coverage of different population groups is necessary to allow better comprehension of the HPV16 genotype-phenotype-disease associations, as well as the direction of HPV16 evolution.

Knowing the genomic sequence of HPV16 as the prevalent circulating hrHPV is crucial to support informed monitoring of the effects of HPV vaccination, including identification of breakthrough cases of vaccine ineffectiveness, and subsequent optimization/”updating” of the currently used HPV vaccines to efficiently combat cervical, as well as oropharyngeal, and other hrHPV-related cancers. Based on this pilot study, we can state that for the Latvian HPV16 isolates, AAS identified in the major capsid protein L1, a viral protein constituting the HPV vaccine, were not associated with vaccine escape. In proof of this, the frequency of AAS in L1 in-going and L2 not in-going into the HPV vaccine did not differ (the latter tended to be even higher), altogether showing that HPV16 isolates circulating in Latvia bear no signs of variability resulting from the immune pressure enforced by HPV vaccination. This is an important finding that informs public health care systems in Latvia about the continued effectiveness of HPV vaccine protection.

## 6. Limitations

The main limitation of the study is the low number of observations and potential sampling biases, as both the main study cohort (Table 1) and the replicating cohort (Table 2) were assembled through hospital-based recruitment in one city, Riga. More sequences from patients with less severe disease, different population groups, as well as those belonging to different regions of Latvia, would have made the study more representative of a broader Latvian population. Such broader study would allow for definitive conclusions on the association of individual SNPs, leading or not leading to AAS, with the clinical course and outcome of cervical disease.

Another limitation is the performance of multiple statistical analyses on one and the same set of 16 WGS sequences of HPV16. The authors first showed an absence of co-variation between nucleotides in any positions of the full genomic sequences of the European HPV16 lineage A. Based on this, statistical analysis of the frequency of occurrence of single SNPs, leading or not leading to AAS, was performed for individual ORFs under the assumption of their independence from each other, as demonstrated by the absence of co-variances, which is justified but formally incorrect. The fact that each ORF (E1, E2, E5, E6, L1, L2) was subjected to analysis of the frequency of occurrence of one single SNP per ORF removed the necessity to introduce multiple corrections. Although the assumption of ORFs as independent units was motivated by the absence of co-variances, these ORFs still belonged to one and the same genome, requesting the analysis with multiple comparisons across the whole genome sequences with necessary corrections.

Multiple comparisons of sequence features/parameters on the sequences of 16 HPV16 genomes may lead to an increased probability of at least one false-positive error across all hypotheses of interest, known as the family-wise type-I error rate (FWER). FWER is defined as the probability of making at least one false-positive conclusion among all the multiple hypotheses being tested [63]. With this, we faced a risk of false interpreting a result, specifically the prevalence of certain SNPs in different forms of cervical disease, as definitive and ‘confirmatory’ for any single parameter found to differ significantly among many tested. In this study, it relied to finding the prevalence of E6: L90V being increased in SCC compared to CINII/III cases (although insignificantly). Based on the recommendations for multiple testing adjustment in multi-arm trials by Howard D.R. et al. [63] and the rationale and justification for non-adjustment for multiple testing in multi-arm exploratory trials by Parker R.A. and Weir S.J. [62], we addressed this problem through replication and repeated experiments aimed at confirming the result. A replication and repeated experiments on an independent dataset were performed, confirming that E6: L90V was indeed more frequent in SCC grade 2/3 than in the less severe cervical disease.

An unfortunate limitation of the study was the absence of access to the clinical data of the patients in the retrospective cohort, which did not allow us to draw any conclusions on the prognostic value of SNPs and AAS in HPV16 E6. It would be undeniably interesting to extend the present study to a wider patient cohort, which would allow us to see whether the unique mutations identified might be more than merely “random” host-specific one-off events.

The presence of one and the same AAS in isolates not sharing a recent common ancestor, specifically AAS associated with the course and outcome of cervical disease, suggests specific selective pressures of currently unknown origin resulting in the convergence of HPV16, as observed in the Latvian HPV16 variants. In this study, we proposed two selective pressures acting on E6, namely the drive for more efficient p53 degradation and escape from the cytotoxic T-cell response. Large-scale studies are needed to gather proof of the realization of these evolutionary pressures. Their identification would reveal novel HPV16 genotype-phenotype-disease associations, assisting in the prognosis of the course and outcome of HPV infection.

## Figures and Tables

**Table 1:** Characteristics of the cervical tissue samples of the prospectively followed patients (n = 16) from which DNA was extracted and found suitable for whole genome sequencing (WGS). Pathomorphological classifications: squamous cell carcinomas (SCC) grades 1 to 3 (G1 to G3; n = 10), SCC in situ (n = 1), cervical intraepithelial lesions of stage III (CINIII; n = 5). WGS data for each of the patients has been entered into GenBank with respective GenBank accession codes.

Patient Code	Age at Sampling/Diagnosis (Full Years)	Year of Sampling	Pathomorpho-Logical Classification	HR HPV Genotype, Virus Load *	Sample Code	GenBank Accession No.
OM1957	54	2012	SCC G2	HPV16 (Ct 19)	OMA1957-B10	PV809647
KV1957	56	2013	SCC G1	HPV16 (Ct 20)	KBA1957-14	PV809648
VV1971	44	2016	SCC G2	HPV16 (Ct 18)	BBH1971-B15	PV809649
KE1980	33	2014	CINIII/HSIL carcinoma in situ	HPV16 (Ct 18)	KEA1980-B32	PV809650
JO1976	44	2020	SCC G3	HPV16 high (Ct < 31) **, HPV33 low, HPV39 medium	JO1976-S11819Nr2	PV809651
II1977	46	2023	SCC G2	HPV16 high (Ct < 31) **, HPV33 low, HPV39 medium	II1977/S11819Nr4	PV809652
SB1997	26	2023	SCC G2	HPV16 high (Ct < 31) **	SB1997-S11819Nr5	PV809653
VS1969	52	2021	SCC G2	HPV16 high (Ct < 31) **, HPV33 medium, HPV39 medium, HPV56 medium	VS1969-S11819Nr6	PV809654
ID1967	56	2023	SCC G2	HPV16 high (Ct < 31) **	ID1967-S11819Nr7	PV809655
BK1957	66	2023	SCC G2	HPV16 (Ct 26)	BK1957-3-S11819Nr8	PV809656
NR1992	29	2021	CINIII	HPV16 (Ct 26), now PCR neg	NR1992-1-S11819Nr9	PV809657
KM1991	30	2019	CINIII	HPV16 (Ct 27); now PCR neg	KM1991-10-S11819Nr10	PV809658
JG1986	33	2019	CINIII	In 2019 HPV16 (Ct 27) + HPV39 (Ct 34); now PCR neg	JG1986-2-S11819Nr11	PV809659
NS1986	27	2024	CINIII	HPV16 (Ct 23)	NS1986-S11819Nr12	PV809660
JO1980	37	2017	SCC G2	HPV16 (Ct 19)	YuOB1980-B4	PV809661
JD1989	39	2015	SCC G2	HPV16 (Ct 18)	JD1989-NNN	PV809662

*—Quantitative loads of hrHPVs according to AllPlex PCR (Seegene, Seoul, Republic of Korea). **—Semiquantitative hrHPV load according to AnuPlex II PCR (Seegene; see Section 2.2 for details); Ct < 31 according to Oštrbenk Valenčak A. et al. 2018 and 2024 [35,36].

**Table 2:** Characteristics of patients whose cervical tissue samples were selected from the archive of Riga Stradins University Clinical Hospital (Riga, Latvia) for having CIN stages I to III (CINI-III) or squamous cell carcinomas of grades 1 to 3 (SCC G1-G3) (n = 31), and DNA was extracted and successfully sequenced by the Sanger method in the region encoding oncoproteins E6/E7. Nucleotide sequences encoding oncoprotein E6/E7 were entered into the GenBank with the given GenBank accession codes.

No.	Patient Code			Pt Age at Sampling	Year of Sample Collection	Clinical Diagnosis	Material	HR HPV Summary (Seegene AnyPlex II) *	GenBank Accession No.
1	JA1985	xxxx-	12012021	36	2021	SCC G2	Biopsy	16(HIGH)	PQ215484
2	TR1949	xxxx-	11022020	71	2020	SCC G2	Biopsy	16(HIGH)	PQ215485
3	VN1933	xxxx-	20072020	87	2020	SCC G2	Biopsy	16(HIGH)	PQ215486
4	LU1956	xxxx-	26082020	64	2020	SCC G2	Biopsy	16(HIGH)	PQ215487
5	JR1981	xxxx-	13102020	39	2020	CIN2	Electroexcision	16(HIGH)	PQ215488
6	EH1995	xxxx-	23102020	25	2020	CIN1	Biopsy	16(HIGH)	PQ215489
7	AL1965	xxxx-	23012019	54	2019	SCC G3	Biopsy	16(HIGH)	PQ215490
8	IV1965	xxxx-	29012019	54	2019	SCC G3	Biopsy	16(HIGH)	PQ215491
9	RŠZ1957	xxxx-	11032019	62	2019	SCC G3	Biopsy	16(HIGH)	PQ215492
10	AG1956	xxxx-	27032019	63	2019	SCC G2	Biopsy	16(HIGH)	PQ215493
11	ZPV1981	xxxx-	04042019	38	2019	CIN2	Electroexcision	16(HIGH)	PQ215494
12	MK1986	xxxx-	13052019	33	2019	CIN3	Electroexcision	16(HIGH)	PQ215495
13	SS1967	xxxx-	23052019	52	2019	SCC G3	Biopsy	16(HIGH)	PQ215496
14	ET1982	xxxx-	12062019	37	2019	SCC G2	Biopsy	16(HIGH)	PQ215497
15	LR1961	xxxx-	17072019	57	2019	SCC G2	Biopsy	16(HIGH)	PQ215498
16	DL1989	xxxx-	12022018	29	2018	CIN3	Complete ectomy	16(HIGH)	PQ215499
17	NK1937	xxxx-	16032018	81	2018	SCC G2	Biopsy	16(HIGH)	PQ215500
18	SC1981	xxxx-	21052018	37	2018	SCC G3	Biopsy	16(HIGH)	PQ215501
19	LP1974	xxxx-	15062018	43	2018	CIN3	Electroexcision	16(HIGH)	PQ215502
20	GS1948	xxxx-	21082018	69	2018	SCC G3	Biopsy	16(HIGH)	PQ215503
21	AS1954	xxxx-	28082018	64	2018	SCC G3	Biopsy	16(HIGH)	PQ215504
22	SJ1973	xxxx-	25092018	45	2018	SCC G3	Biopsy	16(HIGH)	PQ215505
23	SE1953	xxxx-	20012017	63	2017	SCC G2	Biopsy	16(HIGH)	PQ215506
24	JG1972	xxxx-	22052017	44	2017	SCC G2	Biopsy	16(HIGH)	PQ215507
25	DLN1992	xxxx-	24072017	25	2017	CIN3	Electroexcision	16(HIGH)	PQ215508
26	LR1968	xxxx-	20062016	48	2016	CIN3	Biopsy	16(MEDIUM)	PQ215509
27	VR1957	xxxx-	07092016	59	2016	SCC G2	Biopsy	16(MEDIUM)	PQ215510
28	VI1937	xxxx-	21122016	79	2016	SCC G2	Biopsy	16(HIGH)	PQ215511
29	SG1979	xxxx-	13032022	43	2022	SCC G2	Biopsy	16(HIGH), 33(LOW)	PQ215512
30	LG1952	xxxx-	09052022	70	2022	SCC G2	Biopsy	16(HIGH), 33(LOW), 45(MEDIUM)	PQ215513
31	JL1995	xxxx-	29042020	25	2020	CIN3	Electroexcision	16(HIGH), 51(LOW)	PQ215514

*—hrHPV summary presents the semiquantitative hrHPV viral loads according to Anyplex™ II HPV HR Detection assay (Seegene) (see Section 2.2).

**Table 3:** Protein amino acid sequence-altering variants, including single nucleotide polymorphisms (SNPs) and deletions, detected in the Latvian HPV16 isolates. The table compares the frequency of variants resulting in amino acid substitutions for the Latvian HPV16 genomes (n = 16) and completely sequenced HPV16 genomes from elsewhere (n = 4237). Annotation of the affected regions is based on the HPV16 reference NC_001526.4.

Affected Position	Nucleotide Substitution	Variant Type	Affected Protein	Annotation	Frequency in the Latvian Isolates (n, % of n = 16)	Frequency in Other Isolates (n, % of n = 4237)
161	A161C	SNP	E1	E1:N54T	1 (6.25%)	0 (0%) *
189	A189C	SNP	E1	E1:E63D	3 (18.75%)	266 (6.28%)
239	G239T	SNP	E1	E1:R80I	1 (6.25%)	0 (0%)
350	A350G	SNP	E1	E1:K117R	1 (6.25%)	0 (0%)
658	T658A	SNP	E1	E1:S220T	5 (31.25%)	1400 (33.03%)
2501	G2501A	SNP	E2	E2:E204K	1 (6.25%)	35 (0.83%)
2520	T2520C	SNP	E2	E2:I210T	3 (18.75%)	753 (17.77%)
2546	C2546T	SNP	E2	E2:P219S	10 (62.5%)	2812 (66.35%)
2585	G2585A	SNP	E2	E2:E232K	1 (6.25%)	830 (19.58%)
2707	-	deletion	E2; E1^E4	E2:S273-N2780; E1^E4:A79-T86	1 (6.25%)	0 (0%)
2711	T2711G	SNP	E2	E2:S274A	1 (6.25%)	486 (11.47%)
2820	C2820A	SNP	E2	E2:T310K	3 (18.75%)	1412 (33.32%)
2978	A2978C	SNP	E2	E2:M363L	1 (6.25%)	0 (0%)
3115	A3115C	SNP	E5	E5:I44L	9 (56.25%)	1592 (37.56%)
3127	C3127G	SNP	E5	E5:L48V	1 (6.25%)	227 (5.36%)
3178	A3178G	SNP	E5	E5:I65V	10 (62.5%)	2583 (60.95%)
3499	G3499T	SNP	L2	L2:D43Y	1 (6.25%)	0 (0%)
4099	G4099A	SNP	L2	L2:V243I	3 (18.75%)	584 (13.78%)
4177	T4177C	SNP	L2	L2:S269P	3 (18.75%)	1310 (30.91%)
4186	G4186A	SNP	L2	L2:D272N	1 (6.25%)	49 (1.16%)
4362	A4362T	SNP	L2	L2:L330F	10 (62.5%)	1450 (34.21%)
4362	A4362C	SNP	L2	L2:L330F	4 (25%)	1701 (40.14%)
4384	G4384A	SNP	L2	L2:E338K	1 (6.25%)	3 (0.07%)
4417	T4417C	SNP	L2	L2:Y349H	1 (6.25%)	93 (2.19%)
4696	G4696A	SNP	L2	L2:D442N	1 (6.25%)	15 (0.35%)
5354	G5354A	SNP	L1	L1:V194I	1 (6.25%)	39 (0.92%)
5570	A5570G	SNP	L1	L1:T266A	12 (75%)	2892 (68.24%)
6196	G6196C	SNP	L1	L1:L474F	1 (6.25%)	8 (0.19%)
7173	A7173G	SNP	E6	E6:R17G	1 (6.25%)	109 (2.57%)
7174	G7174C	SNP	E6	E6:R17T	1 (6.25%)	31 (0.73%)
7230	G7230C	SNP	E6	E6:E36Q	1 (6.25%)	19 (0.45%)
7392	T7392G	SNP	E6	E6:L90V	7 (43.75%)	1818 (42.9%)

*—colored grey are SNPs and AAS unique to the Latvian HPV16 isolates.

**Table 4:** Synonymous and nonsynonymous SNPs and related AAS observed in >30% of the Latvian HPV16 isolates (Table 1). No difference in the prevalence of AAS in the groups was detected using Fisher’s test.

			SNPs		Sampling Time		Disease Severity	
Genomic Region	ORF	Nucleotide Substitution	AA Substitution	Total No. (in 16)	2012–2019 (n = 7)	2020–2024 (n = 9)	CINIII/G1 (n = 6)	CSCC G2/G3 (n = 10)
Early genes								
	E1	T658A	S220T	5	2 (28.6%)	3 (33.3%)	2 (33.3%)	3 (30%)
	E2	C2546T	P219S	10	4 (57.1%)	6 (66.7%)	3 (30%)	7 (70%)
	E5	A3115C	I44L	9	4 (57.1%)	5 (56.6%)	3 (50%)	6 (60%)
		A3178G	I65V	10	4 (57.1%)	6 (66.7%)	3 (50%)	7 (70%)
	E6	T7392G	L90V	7	2 (28.6%)	5 (56.6%)	1 (16.7%)	6 (60%)
								*p* = 0.1
Late genes								
	L1	A5570G	T266A	10	3 (42.9%)	7 (77.8%)	3 (50%)	7 (70%)
								
	L2	A4362C	L330F	14	5 (71.4%)	9 (100%)	5 (83.3%)	9 (90%)
								

**Table 5:** Prevalence of amino acid substitution E6: L90V in the Latvian HPV16 isolates retrieved from the cervical tissues of women diagnosed with cervical neoplasia and squamous cell carcinomas in 2012–2024.

Patients	n	Diagnosis	No	No with		*p* Value (Chi-Squared Test)
				L90V	L90V, %	
Whole genome sequencing cohort *	16	CINIII/G1	6	1	16.7	
		CSCC G2/G3	10	6	60	*p* = 0.1 *
						
Retrospective study of cervical cancer **	31	CINII-III	8	0	0	
		CINIII/cancer in situ	1	1	100	
		CSCC G2/G3	22	8	36.4	*p* = 0.05 **
						
Pooled cohort	46	CINII/III	13	0	0	
		CSCC G2/G3	32	14	43.8	*p* = 0.0176 ***

* CINIII/G1 versus CSCC G2/G3 (Table 1); ** CINII/III versus CSCC G2/G3 (Table 2); *** CIN II/III versus SCC G2/G3 compared using the “N-1” Chi-squared test as recommended by Campbell (2007) [64].

## Data Availability

The consensus sequences of 16 whole genome Latvian HPV16 isolates were published in GenBank under the accession numbers PV809647 to PV809662 (Table 1) and sequences of 31 E6/E7 genes of the Latvian HPV16 isolates under accession numbers PQ215484 to PQ215514 (Table 2) united in BioProject PRJNA1291997. Accession numbers of other sequences used in this study are listed in the article and its Supplementary Materials.

## Supplementary Materials

The following supporting information can be downloaded at https://www.mdpi.com/article/10.3390/vaccines14060517/s1: Supplementary Table S1. Statistics of the consensus sequences of HPV16 isolates acquired from DNA extracted from the cervical tissue samples of women (n = 16) undergoing a prospective study and found suitable for whole genome sequencing (WGS). Pathomorphological classifications: squamous cell carcinomas (SCC) grades 1 to 3 (G1 to G3; n = 10), SCC in situ (n = 1), cervical intraepithelial lesions of stage III (CINIII; n = 5). The consensus sequences of HPV16 isolates were assembled on the reference genome of HPV16 NC_001526.4. Supplementary Table S2. List of SNPs detected in the individual open reading frames (ORFs) of the Latvian HPV16 isolates. Asynonymous SNPs with respective amino acid substitutions are coloured yellow, and synonymous SNPs with the affected codons are coloured blue. Supplementary Table S3. Involvement of the amino acid residues in the main polymorphic positions of oncoprotein E6 of HPV16 (n = 17) in the protein structure/folding, biological functions including effects on the viral replication and functioning of the host cell, specifically p53 binding and targeting of p53 for proteasomal degradation, recognition by the immune system of the host reflected by the localization of the B- and T-cell epitopes, and effects on the course and outcome of cervical disease, as reported in clinical studies. The table includes a total of 143 observations of the effects of AAS in the polymorphic positions of oncoprotein E6 extracted from 54 publications [67,69,70,71,72,73,74,75,76,77,78,79,80,81,82,83,84,85,86,87,88,89,90,91,92,93,94,95,96,97,98,99,100,101,102,103,104,105,106,107,108,109,110,111,112,113,114,115,116,117]. N/F—not found. Supplementary Table S4. Analysis of the direction of evolution in HPV16 E6 using the SNAP algorithm [57]. Indexes dN and dS reflect the cumulative behaviour of the average synonymous and non-synonymous substitutions. Supplementary Figure S1. Fragment of the midpoint-rooted maximum likelihood tree of the complete HPV16 genomes from LV within the context of complete or near-complete HPV16 isolate genome sequences from elsewhere. Supplementary Figure S2. The cumulative behaviour of the average synonymous and nonsynonymous substitutions (dN/dS graphs) within the E6 gene of 16 Latvian HPV16 isolates analysed by WGS (A), 47 Latvian HPV16 E6 sequences analysed by WGS and Sanger sequencing (B); and 294 E6 sequences of European HPV16 strains (C) presented by XYPLOT. XYPLOT is built using SNAP [57]. dN/dS is a sliding value, and stays constant after position 93 up to the C-terminus of the protein at aa 158. Supplementary Dataset S1. Full midpoint-rooted maximum likelihood tree of the complete HPV16 genomes publicly available in the Newick format. The tree is drawn to scale, and branch lengths correspond to the number of nucleotide substitutions per site. Branches are coloured based on their ultrafast bootstrap (UFBoot) support percentages (out of 1000 replicates) according to the legend. Tip labels are in the form of “Accession|Region (if indicated)|Country (if indicated)|Collection date (YYYY-MM-DD, if indicated)” and are coloured arbitrarily based on the region they represent; Latvian sequences are additionally highlighted with blue rectangles. Multiple sequence alignment used as input for the generation of the tree was performed using MAFFT in FFT-NS-2 (Fast but rough) mode and had 4254 sequences, 14,370 columns, 7520 distinct patterns, 1831 parsimony-informative, 1905 singleton sites, and 10634 constant sites. Maximum-likelihood tree was generated using IQ-TREE with the TVM+F+I+G4 model chosen as the best-fit model according to BIC after the ModelFinder analysis. To be able to see and print separate areas of the tree, choose an area of interest in the tree, then in Acrobat, choose an option to print the file; further, in the choice of pages to be printed, select the option “more alternatives,” and inside this option, choose to print “actual view” at the desired % of enlargement.

## Abbreviations

The following abbreviations are used in this manuscript:

HPV	Human papillomavirus
hrHPV	High-risk human papillomaviruses
HPV16	Human papillomavirus type 16
CC	Cervical cancer
SCC	Squamous cell carcinoma
CIN	Cervical intraepithelial lesion
WGS	Whole-genome sequencing
ORF	Open reading frame
SNP	Single-nucleotide polymorphism
AAS	Amino acid substitution
