# Supplementary material for: Complete Genomes of Human Papillomavirus Type 16 Viruses Isolated from Cases of Cervical Neoplasia and Squamous Cell Carcinomas Followed in Latvia in 2012–2024
Source: Vaccines (Basel). 2026 Jun 9;14(6):517. doi: 10.3390/vaccines14060517 (PMC13307757; doi:10.3390/vaccines14060517)
Supplement: Supplementary file 1 [file vaccines-14-00517-s001.zip › Supplementary Figure S1.pdf]

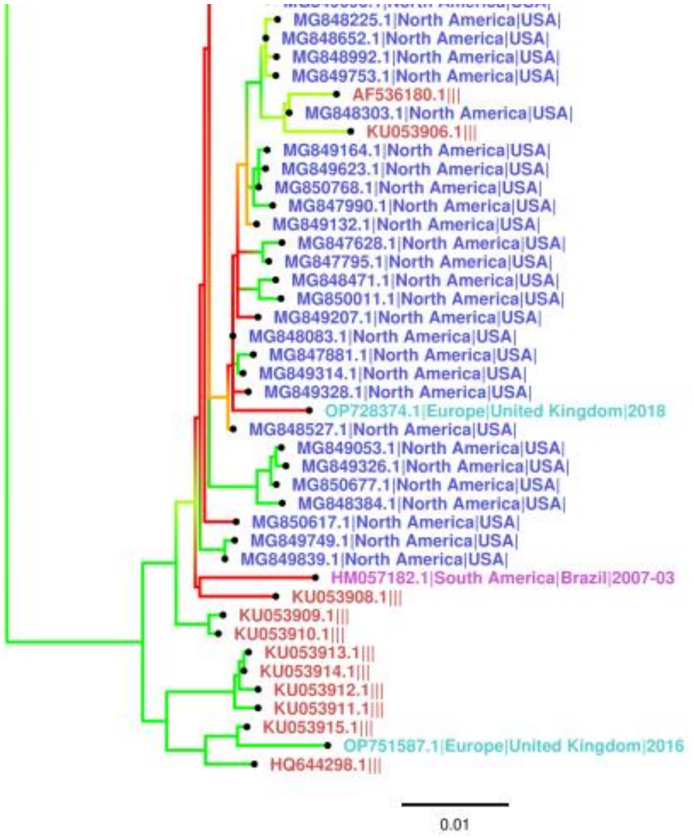

Supplementary Figure S1. Fragment of the midpoint-rooted maximum likelihood tree of the complete HPV16 genomes from LV within the context of complete or near-complete HPV16 isolate genome sequences from elsewhere.

The tree is drawn to scale, and branch lengths correspond to the number of nucleotide substitutions per site. Branches are coloured based on their ultrafast bootstrap (UFBoot) support percentages (out of 1000 replicates) according to the legend. Tip labels are in the form of "Accession|Region (if indicated)|Country (if indicated)|Collection date (YYYY-MM-DD, if indicated)" and are coloured arbitrarily based on the region they represent; Latvian sequences are additionally highlighted by blue rectangles. Multiple sequence alignment used as input for the generation of the tree was performed using MAFFT in FFT-NS-2 (Fast but rough) mode and had 4254 sequences, 14370 columns, 7520 distinct patterns, 1831 parsimony-informative, 1905 singleton sites, and 10634 constant sites. Maximum-likelihood tree was generated using IQ-TREE with the TVM+F+I+G4 chosen as the best-fit model according to BIC after the ModelFinder analysis. The full-tree is presented in the Supplementary Dataset S1.
