# Supplementary material for: Complete Genomes of Human Papillomavirus Type 16 Viruses Isolated from Cases of Cervical Neoplasia and Squamous Cell Carcinomas Followed in Latvia in 2012–2024
Source: Vaccines (Basel). 2026 Jun 9;14(6):517. doi: 10.3390/vaccines14060517 (PMC13307757; doi:10.3390/vaccines14060517)
Supplement: Supplementary file 1 [file vaccines-14-00517-s001.zip › Supplementary Figure S2.pdf]

A

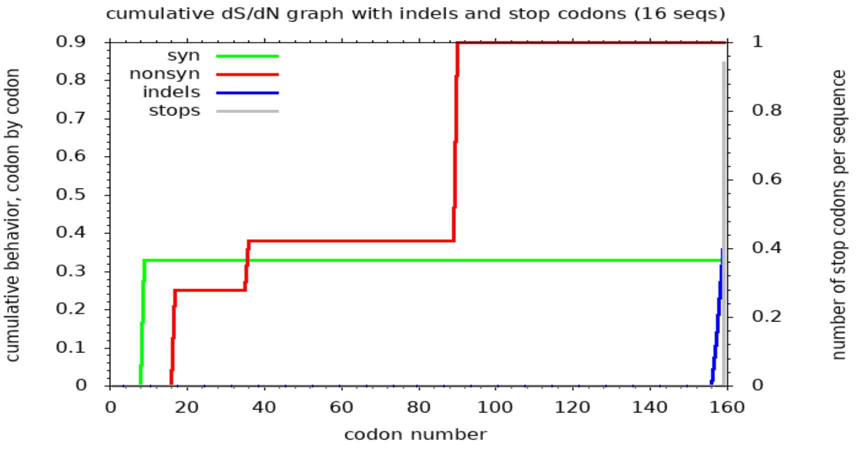

cumulative dS/dN graph with indels and stop codons (16 seqs)
syn
nonsyn
indels
stops
cumulative behavior, codon by codon
number of stop codons per sequence
codon number
0
0.1
0.2
0.3
0.4
0.5
0.6
0.7
0.8
0.9
1
0 20 40 60 80 100 120 140 160

B

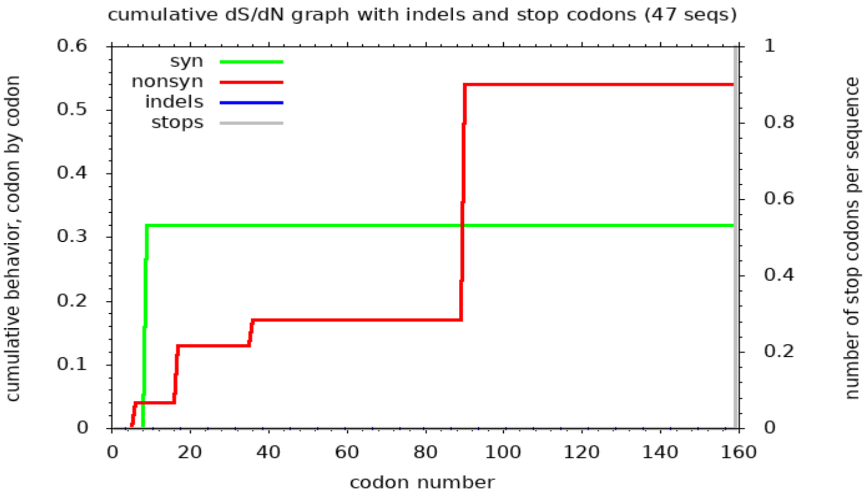

cumulative dS/dN graph with indels and stop codons (47 seqs)
syn
nonsyn
indels
stops
cumulative behavior, codon by codon
number of stop codons per sequence
codon number
0
0.1
0.2
0.3
0.4
0.5
0.6
0.8
1
0 20 40 60 80 100 120 140 160

C

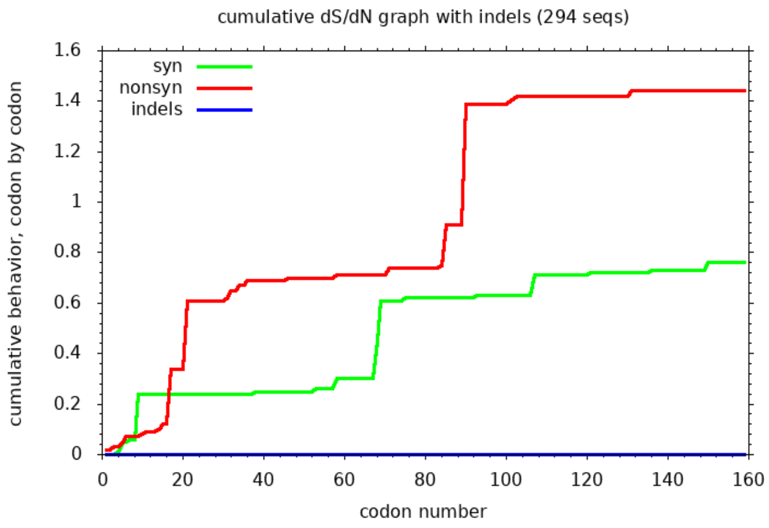

cumulative dS/dN graph with indels (294 seqs)
syn
nonsyn
indels
cumulative behavior, codon by codon
codon number
0
0.2
0.4
0.6
0.8
1
1.2
1.4
1.6
0 20 40 60 80 100 120 140 160

Supplementary Figure S2. The cumulative behaviour of the average synonymous and nonsynonymous substitutions (dN/dS graphs) within E6 gene of 16 Latvian HPV16 isolates analysed by WGS (A), 47 Latvian HPV16 E6 sequences analysed by WGS and Sanger sequencing (B); and 294 E6 sequences of European HPV16 strains (C) presented by XYPLOT. XYPLOT is built using SNAP [57], [59]. dN/dS is a sliding value, and stays constant after position 93 up to the C-terminus of the protein at aa 158.
