# Supplementary material for: Complete Genomes of Human Papillomavirus Type 16 Viruses Isolated from Cases of Cervical Neoplasia and Squamous Cell Carcinomas Followed in Latvia in 2012–2024
Source: Vaccines (Basel). 2026 Jun 9;14(6):517. doi: 10.3390/vaccines14060517 (PMC13307757; doi:10.3390/vaccines14060517)
Supplement: Supplementary file 1 [file Supplementary Dataset S1.pdf › Supplementary Dataset S1.pdf]

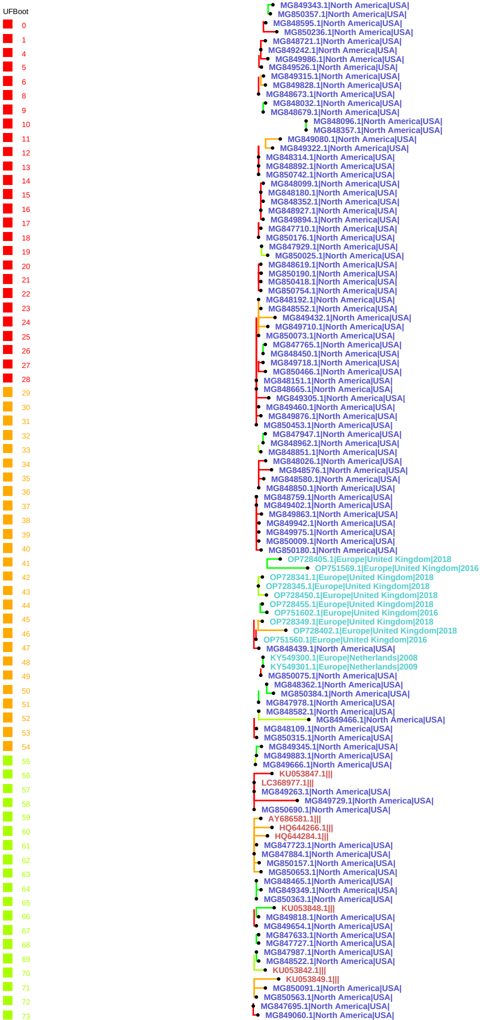
UFBoot
0
1
4
5
6
8
9
10
11
12
13
14
15
16
17
18
19
20
21
22
23
24
25
26
27
28
29
30
31
32
33
34
35
36
37
38
39
40
41
42
43
44
45
46
47
48
49
50
51
52
53
54
55
56
57
58
59
60
61
62
63
64
65
66
67
68
69
70
71
72
73
MG849343.1|North America|USA|
MG850357.1|North America|USA|
MG848595.1|North America|USA|
MG850236.1|North America|USA|
MG848721.1|North America|USA|
MG849242.1|North America|USA|
MG849986.1|North America|USA|
MG849526.1|North America|USA|
MG849315.1|North America|USA|
MG849828.1|North America|USA|
MG848673.1|North America|USA|
MG848032.1|North America|USA|
MG848679.1|North America|USA|
MG848096.1|North America|USA|
MG848357.1|North America|USA|
MG849080.1|North America|USA|
MG849322.1|North America|USA|
MG848314.1|North America|USA|
MG848892.1|North America|USA|
MG850742.1|North America|USA|
MG848099.1|North America|USA|
MG848180.1|North America|USA|
MG848352.1|North America|USA|
MG848927.1|North America|USA|
MG849894.1|North America|USA|
MG847710.1|North America|USA|
MG850176.1|North America|USA|
MG847929.1|North America|USA|
MG850025.1|North America|USA|
MG848619.1|North America|USA|
MG850190.1|North America|USA|
MG850418.1|North America|USA|
MG850754.1|North America|USA|
MG848192.1|North America|USA|
MG848552.1|North America|USA|
MG849432.1|North America|USA|
MG849710.1|North America|USA|
MG850073.1|North America|USA|
MG847765.1|North America|USA|
MG848450.1|North America|USA|
MG849718.1|North America|USA|
MG850466.1|North America|USA|
MG848151.1|North America|USA|
MG848665.1|North America|USA|
MG849305.1|North America|USA|
MG849460.1|North America|USA|
MG849876.1|North America|USA|
MG850453.1|North America|USA|
MG847947.1|North America|USA|
MG848962.1|North America|USA|
MG848851.1|North America|USA|
MG848026.1|North America|USA|
MG848576.1|North America|USA|
MG848580.1|North America|USA|
MG848850.1|North America|USA|
MG848759.1|North America|USA|
MG849402.1|North America|USA|
MG849863.1|North America|USA|
MG849942.1|North America|USA|
MG849975.1|North America|USA|
MG850009.1|North America|USA|
MG850180.1|North America|USA|
OP728405.1|Europe|United Kingdom|2018
OP751569.1|Europe|United Kingdom|2016
OP728341.1|Europe|United Kingdom|2018
OP728345.1|Europe|United Kingdom|2018
OP728450.1|Europe|United Kingdom|2018
OP728455.1|Europe|United Kingdom|2018
OP751602.1|Europe|United Kingdom|2016
OP728349.1|Europe|United Kingdom|2018
OP728402.1|Europe|United Kingdom|2018
OP751560.1|Europe|United Kingdom|2016
MG848439.1|North America|USA|
KY549300.1|Europe|Netherlands|2008
KY549301.1|Europe|Netherlands|2009
MG850075.1|North America|USA|
MG848362.1|North America|USA|
MG850384.1|North America|USA|
MG847978.1|North America|USA|
MG848582.1|North America|USA|
MG849466.1|North America|USA|
MG848109.1|North America|USA|
MG850315.1|North America|USA|
MG849345.1|North America|USA|
MG849883.1|North America|USA|
MG849666.1|North America|USA|
KU053847.1|||
LC368977.1|||
MG849263.1|North America|USA|
MG849729.1|North America|USA|
MG850690.1|North America|USA|
AY686581.1|||
HQ644266.1|||
HQ644284.1|||
MG847723.1|North America|USA|
MG847884.1|North America|USA|
MG850157.1|North America|USA|
MG850653.1|North America|USA|
MG848465.1|North America|USA|
MG849349.1|North America|USA|
MG850363.1|North America|USA|
KU053848.1|||
MG849818.1|North America|USA|
MG849654.1|North America|USA|
MG847633.1|North America|USA|
MG847727.1|North America|USA|
MG847987.1|North America|USA|
MG848522.1|North America|USA|
KU053842.1|||
KU053849.1|||
MG850091.1|North America|USA|
MG850563.1|North America|USA|
MG847695.1|North America|USA|
MG849060.1|North America|USA|

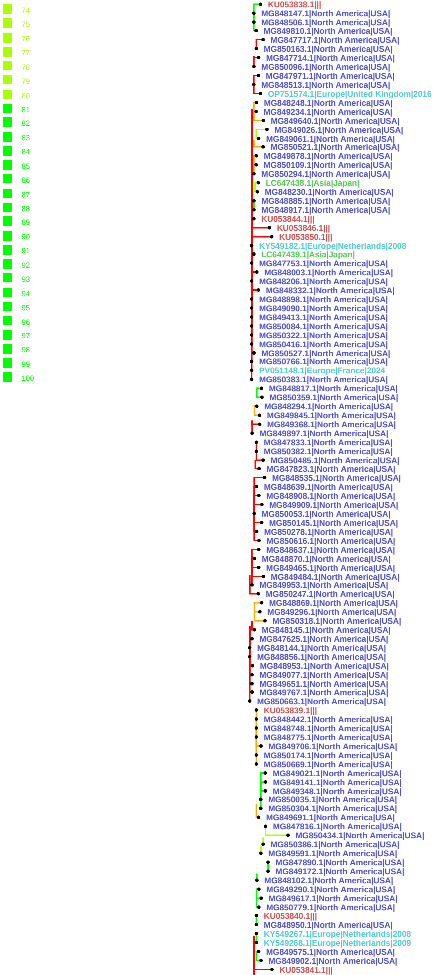
74
75
76
77
78
79
80
81
82
83
84
85
86
87
88
89
90
91
92
93
94
95
96
97
98
99
100
KU053838.1|||
MG848147.1|North America|USA|
MG848506.1|North America|USA|
MG849810.1|North America|USA|
MG847717.1|North America|USA|
MG850163.1|North America|USA|
MG847714.1|North America|USA|
MG850096.1|North America|USA|
MG847971.1|North America|USA|
MG848513.1|North America|USA|
OP751574.1|Europe|United Kingdom|2016
MG848248.1|North America|USA|
MG849234.1|North America|USA|
MG849640.1|North America|USA|
MG849026.1|North America|USA|
MG849061.1|North America|USA|
MG850521.1|North America|USA|
MG849878.1|North America|USA|
MG850109.1|North America|USA|
MG850294.1|North America|USA|
LC647438.1|Asia|Japan|
MG848230.1|North America|USA|
MG848885.1|North America|USA|
MG848917.1|North America|USA|
KU053844.1|||
KU053846.1|||
KU053850.1|||
KY549182.1|Europe|Netherlands|2008
LC647439.1|Asia|Japan|
MG847753.1|North America|USA|
MG848003.1|North America|USA|
MG848206.1|North America|USA|
MG848332.1|North America|USA|
MG848898.1|North America|USA|
MG849090.1|North America|USA|
MG849413.1|North America|USA|
MG850084.1|North America|USA|
MG850322.1|North America|USA|
MG850416.1|North America|USA|
MG850527.1|North America|USA|
MG850766.1|North America|USA|
PV051148.1|Europe|France|2024
MG850383.1|North America|USA|
MG848817.1|North America|USA|
MG850359.1|North America|USA|
MG848294.1|North America|USA|
MG849845.1|North America|USA|
MG849368.1|North America|USA|
MG849897.1|North America|USA|
MG847833.1|North America|USA|
MG850382.1|North America|USA|
MG850485.1|North America|USA|
MG847823.1|North America|USA|
MG848535.1|North America|USA|
MG848639.1|North America|USA|
MG848908.1|North America|USA|
MG849909.1|North America|USA|
MG850053.1|North America|USA|
MG850145.1|North America|USA|
MG850278.1|North America|USA|
MG850616.1|North America|USA|
MG848637.1|North America|USA|
MG848870.1|North America|USA|
MG849465.1|North America|USA|
MG849484.1|North America|USA|
MG849953.1|North America|USA|
MG850247.1|North America|USA|
MG848869.1|North America|USA|
MG849296.1|North America|USA|
MG850318.1|North America|USA|
MG848145.1|North America|USA|
MG847625.1|North America|USA|
MG848144.1|North America|USA|
MG848856.1|North America|USA|
MG848953.1|North America|USA|
MG849077.1|North America|USA|
MG849651.1|North America|USA|
MG849767.1|North America|USA|
MG850663.1|North America|USA|
KU053839.1|||
MG848442.1|North America|USA|
MG848748.1|North America|USA|
MG848775.1|North America|USA|
MG849706.1|North America|USA|
MG850174.1|North America|USA|
MG850669.1|North America|USA|
MG849021.1|North America|USA|
MG849141.1|North America|USA|
MG849348.1|North America|USA|
MG850035.1|North America|USA|
MG850304.1|North America|USA|
MG849691.1|North America|USA|
MG847816.1|North America|USA|
MG850434.1|North America|USA|
MG850386.1|North America|USA|
MG849591.1|North America|USA|
MG847890.1|North America|USA|
MG849172.1|North America|USA|
MG848102.1|North America|USA|
MG849290.1|North America|USA|
MG849617.1|North America|USA|
MG850779.1|North America|USA|
KU053840.1|||
MG848950.1|North America|USA|
KY549267.1|Europe|Netherlands|2008
KY549268.1|Europe|Netherlands|2009
MG849575.1|North America|USA|
MG849902.1|North America|USA|
KU053841.1|||

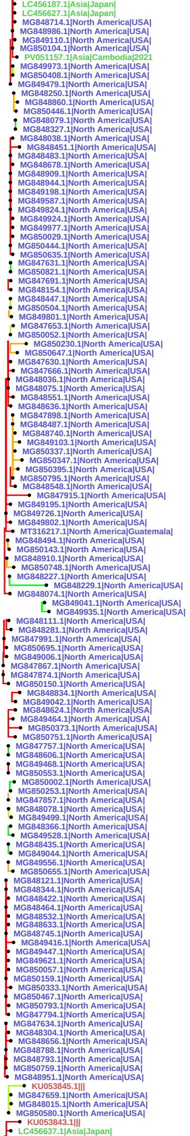

LC456187.1|Asia|Japan|
LC456627.1|Asia|Japan|
MG848714.1|North America|USA|
MG848986.1|North America|USA|
MG849110.1|North America|USA|
MG850104.1|North America|USA|
PV051157.1|Asia|Cambodia|2021
MG849973.1|North America|USA|
MG850408.1|North America|USA|
MG849479.1|North America|USA|
MG848250.1|North America|USA|
MG848860.1|North America|USA|
MG850446.1|North America|USA|
MG848079.1|North America|USA|
MG848327.1|North America|USA|
MG848038.1|North America|USA|
MG848451.1|North America|USA|
MG848483.1|North America|USA|
MG848678.1|North America|USA|
MG848909.1|North America|USA|
MG848944.1|North America|USA|
MG849198.1|North America|USA|
MG849587.1|North America|USA|
MG849824.1|North America|USA|
MG849924.1|North America|USA|
MG849977.1|North America|USA|
MG850029.1|North America|USA|
MG850444.1|North America|USA|
MG850635.1|North America|USA|
MG847631.1|North America|USA|
MG850821.1|North America|USA|
MG847691.1|North America|USA|
MG848154.1|North America|USA|
MG848447.1|North America|USA|
MG850504.1|North America|USA|
MG849801.1|North America|USA|
MG847653.1|North America|USA|
MG850052.1|North America|USA|
MG850230.1|North America|USA|
MG850647.1|North America|USA|
MG847630.1|North America|USA|
MG847666.1|North America|USA|
MG848036.1|North America|USA|
MG848075.1|North America|USA|
MG848551.1|North America|USA|
MG848636.1|North America|USA|
MG847898.1|North America|USA|
MG848487.1|North America|USA|
MG848740.1|North America|USA|
MG849103.1|North America|USA|
MG850337.1|North America|USA|
MG850347.1|North America|USA|
MG850395.1|North America|USA|
MG850795.1|North America|USA|
MG848548.1|North America|USA|
MG847915.1|North America|USA|
MG849195.1|North America|USA|
MG849726.1|North America|USA|
MG849802.1|North America|USA|
MT316217.1|North America|Guatemala|
MG848494.1|North America|USA|
MG850143.1|North America|USA|
MG848910.1|North America|USA|
MG850748.1|North America|USA|
MG848227.1|North America|USA|
MG848229.1|North America|USA|
MG848074.1|North America|USA|
MG849041.1|North America|USA|
MG849935.1|North America|USA|
MG848111.1|North America|USA|
MG848281.1|North America|USA|
MG847991.1|North America|USA|
MG850695.1|North America|USA|
MG849006.1|North America|USA|
MG847867.1|North America|USA|
MG847874.1|North America|USA|
MG850150.1|North America|USA|
MG848834.1|North America|USA|
MG849042.1|North America|USA|
MG848624.1|North America|USA|
MG849464.1|North America|USA|
MG850373.1|North America|USA|
MG850751.1|North America|USA|
MG847757.1|North America|USA|
MG848606.1|North America|USA|
MG849468.1|North America|USA|
MG850553.1|North America|USA|
MG850002.1|North America|USA|
MG850253.1|North America|USA|
MG847857.1|North America|USA|
MG848078.1|North America|USA|
MG849499.1|North America|USA|
MG848366.1|North America|USA|
MG849528.1|North America|USA|
MG848435.1|North America|USA|
MG849044.1|North America|USA|
MG849556.1|North America|USA|
MG850655.1|North America|USA|
MG848121.1|North America|USA|
MG848344.1|North America|USA|
MG848422.1|North America|USA|
MG848464.1|North America|USA|
MG848532.1|North America|USA|
MG848633.1|North America|USA|
MG848745.1|North America|USA|
MG849416.1|North America|USA|
MG849447.1|North America|USA|
MG849621.1|North America|USA|
MG850057.1|North America|USA|
MG850159.1|North America|USA|
MG850333.1|North America|USA|
MG850467.1|North America|USA|
MG850793.1|North America|USA|
MG847794.1|North America|USA|
MG847634.1|North America|USA|
MG848304.1|North America|USA|
MG848656.1|North America|USA|
MG848788.1|North America|USA|
MG848793.1|North America|USA|
MG850759.1|North America|USA|
MG848951.1|North America|USA|
KU053845.1|||
MG847659.1|North America|USA|
MG848015.1|North America|USA|
MG850580.1|North America|USA|
KU053843.1|||
LC456637.1|Asia|Japan|

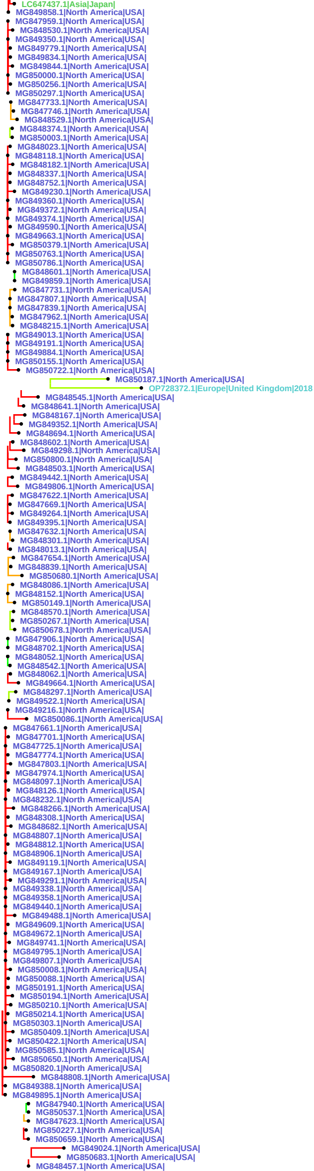
LC647437.1|Asia|Japan|
MG849858.1|North America|USA|
MG847959.1|North America|USA|
MG848530.1|North America|USA|
MG849350.1|North America|USA|
MG849779.1|North America|USA|
MG849834.1|North America|USA|
MG849844.1|North America|USA|
MG850000.1|North America|USA|
MG850256.1|North America|USA|
MG850297.1|North America|USA|
MG847733.1|North America|USA|
MG847746.1|North America|USA|
MG848529.1|North America|USA|
MG848374.1|North America|USA|
MG850003.1|North America|USA|
MG848023.1|North America|USA|
MG848118.1|North America|USA|
MG848182.1|North America|USA|
MG848337.1|North America|USA|
MG848752.1|North America|USA|
MG849230.1|North America|USA|
MG849360.1|North America|USA|
MG849372.1|North America|USA|
MG849374.1|North America|USA|
MG849590.1|North America|USA|
MG849663.1|North America|USA|
MG850379.1|North America|USA|
MG850763.1|North America|USA|
MG850786.1|North America|USA|
MG848601.1|North America|USA|
MG849859.1|North America|USA|
MG847731.1|North America|USA|
MG847807.1|North America|USA|
MG847839.1|North America|USA|
MG847962.1|North America|USA|
MG848215.1|North America|USA|
MG849013.1|North America|USA|
MG849191.1|North America|USA|
MG849884.1|North America|USA|
MG850155.1|North America|USA|
MG850722.1|North America|USA|
MG850187.1|North America|USA|
OP728372.1|Europe|United Kingdom|2018
MG848545.1|North America|USA|
MG848641.1|North America|USA|
MG848167.1|North America|USA|
MG849352.1|North America|USA|
MG848694.1|North America|USA|
MG848602.1|North America|USA|
MG849298.1|North America|USA|
MG850800.1|North America|USA|
MG848503.1|North America|USA|
MG849442.1|North America|USA|
MG849806.1|North America|USA|
MG847622.1|North America|USA|
MG847669.1|North America|USA|
MG849264.1|North America|USA|
MG849395.1|North America|USA|
MG847632.1|North America|USA|
MG848301.1|North America|USA|
MG848013.1|North America|USA|
MG847654.1|North America|USA|
MG848839.1|North America|USA|
MG850680.1|North America|USA|
MG848086.1|North America|USA|
MG848152.1|North America|USA|
MG850149.1|North America|USA|
MG848570.1|North America|USA|
MG850267.1|North America|USA|
MG850678.1|North America|USA|
MG847906.1|North America|USA|
MG848702.1|North America|USA|
MG848052.1|North America|USA|
MG848542.1|North America|USA|
MG848062.1|North America|USA|
MG849664.1|North America|USA|
MG848297.1|North America|USA|
MG849522.1|North America|USA|
MG849216.1|North America|USA|
MG850086.1|North America|USA|
MG847661.1|North America|USA|
MG847701.1|North America|USA|
MG847725.1|North America|USA|
MG847774.1|North America|USA|
MG847803.1|North America|USA|
MG847974.1|North America|USA|
MG848097.1|North America|USA|
MG848126.1|North America|USA|
MG848232.1|North America|USA|
MG848266.1|North America|USA|
MG848308.1|North America|USA|
MG848682.1|North America|USA|
MG848807.1|North America|USA|
MG848812.1|North America|USA|
MG848906.1|North America|USA|
MG849119.1|North America|USA|
MG849167.1|North America|USA|
MG849291.1|North America|USA|
MG849338.1|North America|USA|
MG849358.1|North America|USA|
MG849440.1|North America|USA|
MG849488.1|North America|USA|
MG849609.1|North America|USA|
MG849672.1|North America|USA|
MG849741.1|North America|USA|
MG849795.1|North America|USA|
MG849807.1|North America|USA|
MG850008.1|North America|USA|
MG850088.1|North America|USA|
MG850191.1|North America|USA|
MG850194.1|North America|USA|
MG850210.1|North America|USA|
MG850214.1|North America|USA|
MG850303.1|North America|USA|
MG850409.1|North America|USA|
MG850422.1|North America|USA|
MG850585.1|North America|USA|
MG850650.1|North America|USA|
MG850820.1|North America|USA|
MG848808.1|North America|USA|
MG849388.1|North America|USA|
MG849895.1|North America|USA|
MG847940.1|North America|USA|
MG850537.1|North America|USA|
MG847623.1|North America|USA|
MG850227.1|North America|USA|
MG850659.1|North America|USA|
MG849024.1|North America|USA|
MG850683.1|North America|USA|
MG848457.1|North America|USA|

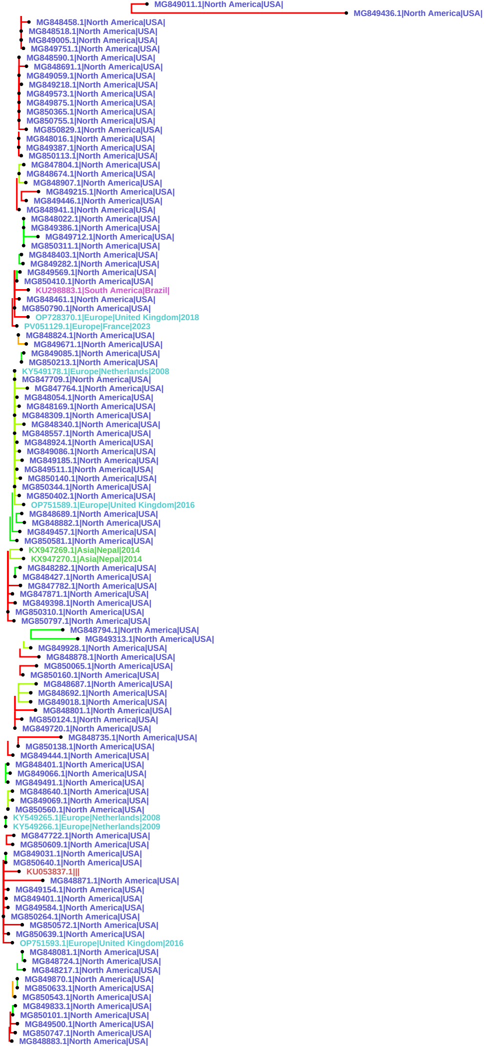

MG849011.1|North America|USA|
MG849436.1|North America|USA|
MG848458.1|North America|USA|
MG848518.1|North America|USA|
MG849005.1|North America|USA|
MG849751.1|North America|USA|
MG848590.1|North America|USA|
MG848691.1|North America|USA|
MG849059.1|North America|USA|
MG849218.1|North America|USA|
MG849573.1|North America|USA|
MG849875.1|North America|USA|
MG850365.1|North America|USA|
MG850755.1|North America|USA|
MG850829.1|North America|USA|
MG848016.1|North America|USA|
MG849387.1|North America|USA|
MG850113.1|North America|USA|
MG847804.1|North America|USA|
MG848674.1|North America|USA|
MG848907.1|North America|USA|
MG849215.1|North America|USA|
MG849446.1|North America|USA|
MG848941.1|North America|USA|
MG848022.1|North America|USA|
MG849386.1|North America|USA|
MG849712.1|North America|USA|
MG850311.1|North America|USA|
MG848403.1|North America|USA|
MG849282.1|North America|USA|
MG849569.1|North America|USA|
MG850410.1|North America|USA|
KU298883.1|South America|Brazil|
MG848461.1|North America|USA|
MG850790.1|North America|USA|
OP728370.1|Europe|United Kingdom|2018
PV051129.1|Europe|France|2023
MG848824.1|North America|USA|
MG849671.1|North America|USA|
MG849085.1|North America|USA|
MG850213.1|North America|USA|
KY549178.1|Europe|Netherlands|2008
MG847709.1|North America|USA|
MG847764.1|North America|USA|
MG848054.1|North America|USA|
MG848169.1|North America|USA|
MG848309.1|North America|USA|
MG848340.1|North America|USA|
MG848557.1|North America|USA|
MG848924.1|North America|USA|
MG849086.1|North America|USA|
MG849185.1|North America|USA|
MG849511.1|North America|USA|
MG850140.1|North America|USA|
MG850344.1|North America|USA|
MG850402.1|North America|USA|
OP751589.1|Europe|United Kingdom|2016
MG848689.1|North America|USA|
MG848882.1|North America|USA|
MG849457.1|North America|USA|
MG850581.1|North America|USA|
KX947269.1|Asia|Nepal|2014
KX947270.1|Asia|Nepal|2014
MG848282.1|North America|USA|
MG848427.1|North America|USA|
MG847782.1|North America|USA|
MG847871.1|North America|USA|
MG849398.1|North America|USA|
MG850310.1|North America|USA|
MG850797.1|North America|USA|
MG848794.1|North America|USA|
MG849313.1|North America|USA|
MG849928.1|North America|USA|
MG848878.1|North America|USA|
MG850065.1|North America|USA|
MG850160.1|North America|USA|
MG848687.1|North America|USA|
MG848692.1|North America|USA|
MG849018.1|North America|USA|
MG848801.1|North America|USA|
MG850124.1|North America|USA|
MG849720.1|North America|USA|
MG848735.1|North America|USA|
MG850138.1|North America|USA|
MG849444.1|North America|USA|
MG848401.1|North America|USA|
MG849066.1|North America|USA|
MG849491.1|North America|USA|
MG848640.1|North America|USA|
MG849069.1|North America|USA|
MG850560.1|North America|USA|
KY549265.1|Europe|Netherlands|2008
KY549266.1|Europe|Netherlands|2009
MG847722.1|North America|USA|
MG850609.1|North America|USA|
MG849031.1|North America|USA|
MG850640.1|North America|USA|
KU053837.1|||
MG848871.1|North America|USA|
MG849154.1|North America|USA|
MG849401.1|North America|USA|
MG849584.1|North America|USA|
MG850264.1|North America|USA|
MG850572.1|North America|USA|
MG850639.1|North America|USA|
OP751593.1|Europe|United Kingdom|2016
MG848081.1|North America|USA|
MG848724.1|North America|USA|
MG848217.1|North America|USA|
MG849870.1|North America|USA|
MG850633.1|North America|USA|
MG850543.1|North America|USA|
MG849833.1|North America|USA|
MG850101.1|North America|USA|
MG849500.1|North America|USA|
MG850747.1|North America|USA|
MG848883.1|North America|USA|

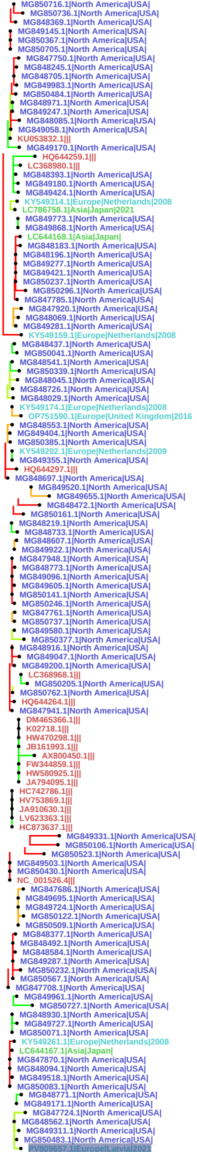
MG850716.1|North America|USA|
MG850736.1|North America|USA|
MG848369.1|North America|USA|
MG849145.1|North America|USA|
MG850367.1|North America|USA|
MG850705.1|North America|USA|
MG847750.1|North America|USA|
MG848245.1|North America|USA|
MG848705.1|North America|USA|
MG849983.1|North America|USA|
MG850484.1|North America|USA|
MG848971.1|North America|USA|
MG849247.1|North America|USA|
MG848085.1|North America|USA|
MG849058.1|North America|USA|
KU053832.1|||
MG849170.1|North America|USA|
HQ644259.1|||
LC368980.1|||
MG848393.1|North America|USA|
MG849180.1|North America|USA|
MG849424.1|North America|USA|
KY549314.1|Europe|Netherlands|2008
LC786758.1|Asia|Japan|2021
MG849773.1|North America|USA|
MG849868.1|North America|USA|
LC644168.1|Asia|Japan|
MG848183.1|North America|USA|
MG848196.1|North America|USA|
MG849277.1|North America|USA|
MG849421.1|North America|USA|
MG850237.1|North America|USA|
MG850296.1|North America|USA|
MG847785.1|North America|USA|
MG847920.1|North America|USA|
MG848069.1|North America|USA|
MG849281.1|North America|USA|
KY549159.1|Europe|Netherlands|2008
MG848437.1|North America|USA|
MG850041.1|North America|USA|
MG848541.1|North America|USA|
MG850339.1|North America|USA|
MG848045.1|North America|USA|
MG848726.1|North America|USA|
MG848029.1|North America|USA|
KY549174.1|Europe|Netherlands|2008
OP751590.1|Europe|United Kingdom|2016
MG848553.1|North America|USA|
MG849404.1|North America|USA|
MG850385.1|North America|USA|
KY549202.1|Europe|Netherlands|2009
MG849355.1|North America|USA|
HQ644297.1|||
MG848697.1|North America|USA|
MG849520.1|North America|USA|
MG849655.1|North America|USA|
MG848472.1|North America|USA|
MG850161.1|North America|USA|
MG848219.1|North America|USA|
MG848733.1|North America|USA|
MG848607.1|North America|USA|
MG849922.1|North America|USA|
MG847948.1|North America|USA|
MG848773.1|North America|USA|
MG849096.1|North America|USA|
MG849605.1|North America|USA|
MG850141.1|North America|USA|
MG850246.1|North America|USA|
MG847761.1|North America|USA|
MG850737.1|North America|USA|
MG849580.1|North America|USA|
MG850377.1|North America|USA|
MG848916.1|North America|USA|
MG849047.1|North America|USA|
MG849200.1|North America|USA|
LC368968.1|||
MG850205.1|North America|USA|
MG850762.1|North America|USA|
HQ644264.1|||
MG847941.1|North America|USA|
DM465366.1|||
K02718.1|||
HW470298.1|||
JB161993.1|||
AX800450.1|||
FW344859.1|||
HW580925.1|||
JA794095.1|||
HC742786.1|||
HV753869.1|||
JA910630.1|||
LV623363.1|||
HC873637.1|||
MG849331.1|North America|USA|
MG850106.1|North America|USA|
MG850523.1|North America|USA|
MG849503.1|North America|USA|
MG850430.1|North America|USA|
NC_001526.4|||
MG847686.1|North America|USA|
MG849695.1|North America|USA|
MG849724.1|North America|USA|
MG850122.1|North America|USA|
MG850509.1|North America|USA|
MG848377.1|North America|USA|
MG848492.1|North America|USA|
MG848584.1|North America|USA|
MG849287.1|North America|USA|
MG850232.1|North America|USA|
MG850567.1|North America|USA|
MG847708.1|North America|USA|
MG849961.1|North America|USA|
MG850727.1|North America|USA|
MG848930.1|North America|USA|
MG849727.1|North America|USA|
MG850071.1|North America|USA|
KY549261.1|Europe|Netherlands|2008
LC644167.1|Asia|Japan|
MG847870.1|North America|USA|
MG848094.1|North America|USA|
MG849518.1|North America|USA|
MG850083.1|North America|USA|
MG848771.1|North America|USA|
MG849171.1|North America|USA|
MG847724.1|North America|USA|
MG848562.1|North America|USA|
MG849311.1|North America|USA|
MG850483.1|North America|USA|
PV809657.1|Europe|Latvia|2021

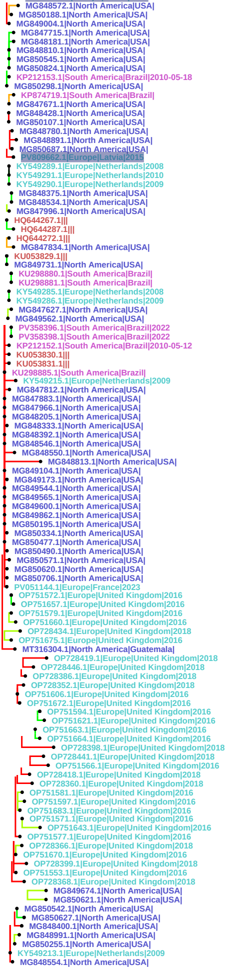
MG848572.1|North America|USA|
MG850188.1|North America|USA|
MG849004.1|North America|USA|
MG847715.1|North America|USA|
MG848181.1|North America|USA|
MG848810.1|North America|USA|
MG850545.1|North America|USA|
MG850824.1|North America|USA|
KP212153.1|South America|Brazil|2010-05-18
MG850298.1|North America|USA|
KP874719.1|South America|Brazil|
MG847671.1|North America|USA|
MG848428.1|North America|USA|
MG850107.1|North America|USA|
MG848780.1|North America|USA|
MG848891.1|North America|USA|
MG850687.1|North America|USA|
PV809662.1|Europe|Latvia|2015
KY549289.1|Europe|Netherlands|2008
KY549291.1|Europe|Netherlands|2010
KY549290.1|Europe|Netherlands|2009
MG848375.1|North America|USA|
MG848534.1|North America|USA|
MG847996.1|North America|USA|
HQ644267.1|||
HQ644287.1|||
HQ644272.1|||
MG847834.1|North America|USA|
KU053829.1|||
MG849731.1|North America|USA|
KU298880.1|South America|Brazil|
KU298881.1|South America|Brazil|
KY549285.1|Europe|Netherlands|2008
KY549286.1|Europe|Netherlands|2009
MG847627.1|North America|USA|
MG849562.1|North America|USA|
PV358396.1|South America|Brazil|2022
PV358398.1|South America|Brazil|2022
KP212152.1|South America|Brazil|2010-05-12
KU053830.1|||
KU053831.1|||
KU298885.1|South America|Brazil|
KY549215.1|Europe|Netherlands|2009
MG847812.1|North America|USA|
MG847883.1|North America|USA|
MG847966.1|North America|USA|
MG848205.1|North America|USA|
MG848333.1|North America|USA|
MG848392.1|North America|USA|
MG848546.1|North America|USA|
MG848550.1|North America|USA|
MG848813.1|North America|USA|
MG849104.1|North America|USA|
MG849173.1|North America|USA|
MG849544.1|North America|USA|
MG849565.1|North America|USA|
MG849600.1|North America|USA|
MG849862.1|North America|USA|
MG850195.1|North America|USA|
MG850334.1|North America|USA|
MG850477.1|North America|USA|
MG850490.1|North America|USA|
MG850571.1|North America|USA|
MG850620.1|North America|USA|
MG850706.1|North America|USA|
PV051144.1|Europe|France|2023
OP751572.1|Europe|United Kingdom|2016
OP751657.1|Europe|United Kingdom|2016
OP751579.1|Europe|United Kingdom|2016
OP751660.1|Europe|United Kingdom|2016
OP728434.1|Europe|United Kingdom|2018
OP751675.1|Europe|United Kingdom|2016
MT316304.1|North America|Guatemala|
OP728419.1|Europe|United Kingdom|2018
OP728446.1|Europe|United Kingdom|2018
OP728386.1|Europe|United Kingdom|2018
OP728352.1|Europe|United Kingdom|2018
OP751606.1|Europe|United Kingdom|2016
OP751672.1|Europe|United Kingdom|2016
OP751594.1|Europe|United Kingdom|2016
OP751621.1|Europe|United Kingdom|2016
OP751663.1|Europe|United Kingdom|2016
OP751664.1|Europe|United Kingdom|2016
OP728398.1|Europe|United Kingdom|2018
OP728441.1|Europe|United Kingdom|2018
OP751566.1|Europe|United Kingdom|2016
OP728418.1|Europe|United Kingdom|2018
OP728360.1|Europe|United Kingdom|2018
OP751581.1|Europe|United Kingdom|2016
OP751597.1|Europe|United Kingdom|2016
OP751683.1|Europe|United Kingdom|2016
OP751571.1|Europe|United Kingdom|2016
OP751643.1|Europe|United Kingdom|2016
OP751577.1|Europe|United Kingdom|2016
OP728366.1|Europe|United Kingdom|2018
OP751670.1|Europe|United Kingdom|2016
OP728399.1|Europe|United Kingdom|2018
OP751553.1|Europe|United Kingdom|2016
OP728368.1|Europe|United Kingdom|2018
MG849674.1|North America|USA|
MG850621.1|North America|USA|
MG850542.1|North America|USA|
MG850627.1|North America|USA|
MG848400.1|North America|USA|
MG848991.1|North America|USA|
MG850255.1|North America|USA|
KY549213.1|Europe|Netherlands|2009
MG848554.1|North America|USA|

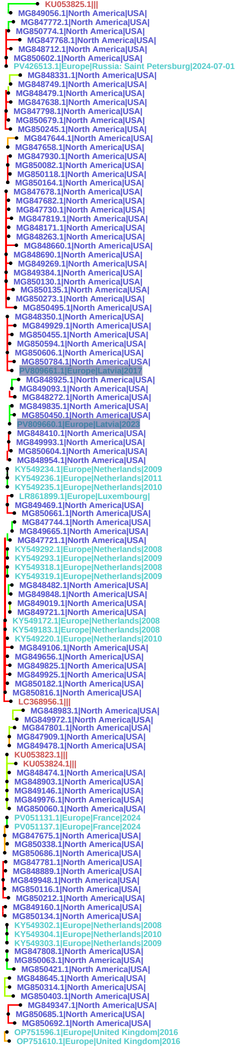
KU053825.1|||
MG849056.1|North America|USA|
MG847772.1|North America|USA|
MG850774.1|North America|USA|
MG847768.1|North America|USA|
MG848712.1|North America|USA|
MG850602.1|North America|USA|
PV426513.1|Europe|Russia: Saint Petersburg|2024-07-01
MG848331.1|North America|USA|
MG848749.1|North America|USA|
MG848479.1|North America|USA|
MG847638.1|North America|USA|
MG847798.1|North America|USA|
MG850679.1|North America|USA|
MG850245.1|North America|USA|
MG847644.1|North America|USA|
MG847658.1|North America|USA|
MG847930.1|North America|USA|
MG850082.1|North America|USA|
MG850118.1|North America|USA|
MG850164.1|North America|USA|
MG847678.1|North America|USA|
MG847682.1|North America|USA|
MG847730.1|North America|USA|
MG847819.1|North America|USA|
MG848171.1|North America|USA|
MG848263.1|North America|USA|
MG848660.1|North America|USA|
MG848690.1|North America|USA|
MG849269.1|North America|USA|
MG849384.1|North America|USA|
MG850130.1|North America|USA|
MG850135.1|North America|USA|
MG850273.1|North America|USA|
MG850495.1|North America|USA|
MG848350.1|North America|USA|
MG849929.1|North America|USA|
MG850455.1|North America|USA|
MG850594.1|North America|USA|
MG850606.1|North America|USA|
MG850784.1|North America|USA|
PV809661.1|Europe|Latvia|2017
MG848925.1|North America|USA|
MG849093.1|North America|USA|
MG848272.1|North America|USA|
MG849835.1|North America|USA|
MG850450.1|North America|USA|
PV809660.1|Europe|Latvia|2023
MG848410.1|North America|USA|
MG849993.1|North America|USA|
MG850604.1|North America|USA|
MG848954.1|North America|USA|
KY549234.1|Europe|Netherlands|2009
KY549236.1|Europe|Netherlands|2011
KY549235.1|Europe|Netherlands|2010
LR861899.1|Europe|Luxembourg|
MG849469.1|North America|USA|
MG850661.1|North America|USA|
MG847744.1|North America|USA|
MG849665.1|North America|USA|
MG847721.1|North America|USA|
KY549292.1|Europe|Netherlands|2008
KY549293.1|Europe|Netherlands|2009
KY549318.1|Europe|Netherlands|2008
KY549319.1|Europe|Netherlands|2009
MG848482.1|North America|USA|
MG849848.1|North America|USA|
MG849019.1|North America|USA|
MG849721.1|North America|USA|
KY549172.1|Europe|Netherlands|2008
KY549183.1|Europe|Netherlands|2008
KY549220.1|Europe|Netherlands|2010
MG849106.1|North America|USA|
MG849656.1|North America|USA|
MG849825.1|North America|USA|
MG849925.1|North America|USA|
MG850182.1|North America|USA|
MG850816.1|North America|USA|
LC368956.1|||
MG848983.1|North America|USA|
MG849972.1|North America|USA|
MG847801.1|North America|USA|
MG847909.1|North America|USA|
MG849478.1|North America|USA|
KU053823.1|||
KU053824.1|||
MG848474.1|North America|USA|
MG848903.1|North America|USA|
MG849146.1|North America|USA|
MG849976.1|North America|USA|
MG850060.1|North America|USA|
PV051131.1|Europe|France|2024
PV051137.1|Europe|France|2024
MG847675.1|North America|USA|
MG850338.1|North America|USA|
MG850686.1|North America|USA|
MG847781.1|North America|USA|
MG848889.1|North America|USA|
MG849948.1|North America|USA|
MG850116.1|North America|USA|
MG850212.1|North America|USA|
MG849160.1|North America|USA|
MG850134.1|North America|USA|
KY549302.1|Europe|Netherlands|2008
KY549304.1|Europe|Netherlands|2010
KY549303.1|Europe|Netherlands|2009
MG847808.1|North America|USA|
MG850063.1|North America|USA|
MG850421.1|North America|USA|
MG848645.1|North America|USA|
MG850314.1|North America|USA|
MG850403.1|North America|USA|
MG849347.1|North America|USA|
MG850685.1|North America|USA|
MG850692.1|North America|USA|
OP751596.1|Europe|United Kingdom|2016
OP751610.1|Europe|United Kingdom|2016

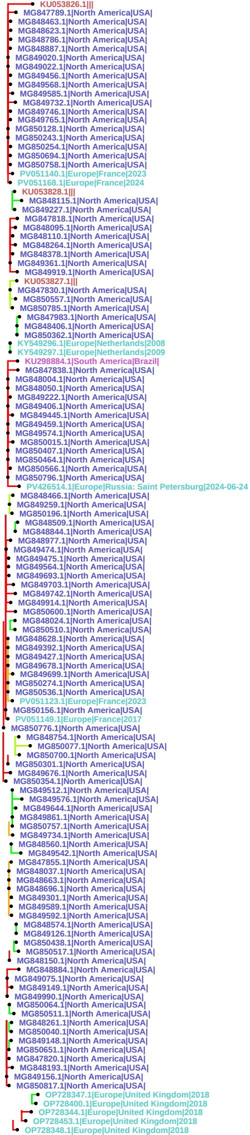
KU053826.1|||
MG847789.1|North America|USA|
MG848463.1|North America|USA|
MG848623.1|North America|USA|
MG848786.1|North America|USA|
MG848887.1|North America|USA|
MG849020.1|North America|USA|
MG849022.1|North America|USA|
MG849456.1|North America|USA|
MG849568.1|North America|USA|
MG849585.1|North America|USA|
MG849732.1|North America|USA|
MG849746.1|North America|USA|
MG849765.1|North America|USA|
MG850128.1|North America|USA|
MG850243.1|North America|USA|
MG850254.1|North America|USA|
MG850694.1|North America|USA|
MG850758.1|North America|USA|
PV051140.1|Europe|France|2023
PV051168.1|Europe|France|2024
KU053828.1|||
MG848115.1|North America|USA|
MG849227.1|North America|USA|
MG847818.1|North America|USA|
MG848095.1|North America|USA|
MG848110.1|North America|USA|
MG848264.1|North America|USA|
MG848378.1|North America|USA|
MG849361.1|North America|USA|
MG849919.1|North America|USA|
KU053827.1|||
MG847830.1|North America|USA|
MG850557.1|North America|USA|
MG850785.1|North America|USA|
MG847983.1|North America|USA|
MG848406.1|North America|USA|
MG850362.1|North America|USA|
KY549296.1|Europe|Netherlands|2008
KY549297.1|Europe|Netherlands|2009
KU298884.1|South America|Brazil|
MG847838.1|North America|USA|
MG848004.1|North America|USA|
MG848050.1|North America|USA|
MG849222.1|North America|USA|
MG849406.1|North America|USA|
MG849445.1|North America|USA|
MG849459.1|North America|USA|
MG849574.1|North America|USA|
MG850015.1|North America|USA|
MG850407.1|North America|USA|
MG850464.1|North America|USA|
MG850566.1|North America|USA|
MG850796.1|North America|USA|
PV426514.1|Europe|Russia: Saint Petersburg|2024-06-24
MG848466.1|North America|USA|
MG849259.1|North America|USA|
MG850196.1|North America|USA|
MG848509.1|North America|USA|
MG848844.1|North America|USA|
MG848977.1|North America|USA|
MG849474.1|North America|USA|
MG849475.1|North America|USA|
MG849564.1|North America|USA|
MG849693.1|North America|USA|
MG849703.1|North America|USA|
MG849742.1|North America|USA|
MG849914.1|North America|USA|
MG850600.1|North America|USA|
MG848024.1|North America|USA|
MG850510.1|North America|USA|
MG848628.1|North America|USA|
MG849392.1|North America|USA|
MG849427.1|North America|USA|
MG849678.1|North America|USA|
MG849699.1|North America|USA|
MG850274.1|North America|USA|
MG850536.1|North America|USA|
PV051123.1|Europe|France|2023
MG850156.1|North America|USA|
PV051149.1|Europe|France|2017
MG850776.1|North America|USA|
MG848754.1|North America|USA|
MG850077.1|North America|USA|
MG850700.1|North America|USA|
MG850301.1|North America|USA|
MG849676.1|North America|USA|
MG850354.1|North America|USA|
MG849512.1|North America|USA|
MG849576.1|North America|USA|
MG849644.1|North America|USA|
MG849861.1|North America|USA|
MG850757.1|North America|USA|
MG849734.1|North America|USA|
MG848560.1|North America|USA|
MG849542.1|North America|USA|
MG847855.1|North America|USA|
MG848037.1|North America|USA|
MG848663.1|North America|USA|
MG848696.1|North America|USA|
MG849301.1|North America|USA|
MG849589.1|North America|USA|
MG849592.1|North America|USA|
MG848574.1|North America|USA|
MG849126.1|North America|USA|
MG850438.1|North America|USA|
MG850517.1|North America|USA|
MG848150.1|North America|USA|
MG848884.1|North America|USA|
MG849075.1|North America|USA|
MG849149.1|North America|USA|
MG849990.1|North America|USA|
MG850064.1|North America|USA|
MG850511.1|North America|USA|
MG848261.1|North America|USA|
MG850040.1|North America|USA|
MG849148.1|North America|USA|
MG850651.1|North America|USA|
MG847820.1|North America|USA|
MG848193.1|North America|USA|
MG849156.1|North America|USA|
MG850817.1|North America|USA|
OP728347.1|Europe|United Kingdom|2018
OP728400.1|Europe|United Kingdom|2018
OP728344.1|Europe|United Kingdom|2018
OP728453.1|Europe|United Kingdom|2018
OP728348.1|Europe|United Kingdom|2018

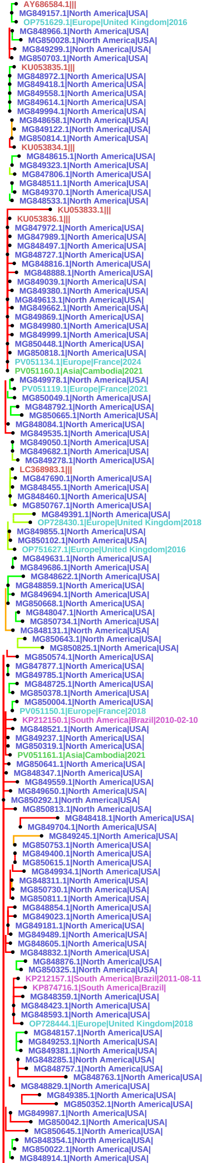

AY686584.1|||
MG849157.1|North America|USA|
OP751629.1|Europe|United Kingdom|2016
MG848966.1|North America|USA|
MG850028.1|North America|USA|
MG849299.1|North America|USA|
MG850703.1|North America|USA|
KU053835.1|||
MG848972.1|North America|USA|
MG849418.1|North America|USA|
MG849558.1|North America|USA|
MG849614.1|North America|USA|
MG849994.1|North America|USA|
MG848658.1|North America|USA|
MG849122.1|North America|USA|
MG850814.1|North America|USA|
KU053834.1|||
MG848615.1|North America|USA|
MG849323.1|North America|USA|
MG847806.1|North America|USA|
MG848511.1|North America|USA|
MG849370.1|North America|USA|
MG848533.1|North America|USA|
KU053833.1|||
KU053836.1|||
MG847972.1|North America|USA|
MG847989.1|North America|USA|
MG848497.1|North America|USA|
MG848727.1|North America|USA|
MG848816.1|North America|USA|
MG848888.1|North America|USA|
MG849039.1|North America|USA|
MG849380.1|North America|USA|
MG849613.1|North America|USA|
MG849662.1|North America|USA|
MG849869.1|North America|USA|
MG849980.1|North America|USA|
MG849999.1|North America|USA|
MG850448.1|North America|USA|
MG850818.1|North America|USA|
PV051134.1|Europe|France|2024
PV051160.1|Asia|Cambodia|2021
MG849978.1|North America|USA|
PV051119.1|Europe|France|2021
MG850049.1|North America|USA|
MG848792.1|North America|USA|
MG850665.1|North America|USA|
MG848084.1|North America|USA|
MG849535.1|North America|USA|
MG849050.1|North America|USA|
MG849682.1|North America|USA|
MG849278.1|North America|USA|
LC368983.1|||
MG847690.1|North America|USA|
MG848455.1|North America|USA|
MG848460.1|North America|USA|
MG850767.1|North America|USA|
MG849391.1|North America|USA|
OP728430.1|Europe|United Kingdom|2018
MG849855.1|North America|USA|
MG850102.1|North America|USA|
OP751627.1|Europe|United Kingdom|2016
MG849631.1|North America|USA|
MG849686.1|North America|USA|
MG848622.1|North America|USA|
MG848859.1|North America|USA|
MG849694.1|North America|USA|
MG850668.1|North America|USA|
MG848047.1|North America|USA|
MG850734.1|North America|USA|
MG848131.1|North America|USA|
MG850643.1|North America|USA|
MG850825.1|North America|USA|
MG850574.1|North America|USA|
MG847877.1|North America|USA|
MG849785.1|North America|USA|
MG848725.1|North America|USA|
MG850378.1|North America|USA|
MG850004.1|North America|USA|
PV051150.1|Europe|France|2018
KP212150.1|South America|Brazil|2010-02-10
MG848521.1|North America|USA|
MG849237.1|North America|USA|
MG850319.1|North America|USA|
PV051161.1|Asia|Cambodia|2021
MG850641.1|North America|USA|
MG848347.1|North America|USA|
MG849559.1|North America|USA|
MG849650.1|North America|USA|
MG850292.1|North America|USA|
MG850813.1|North America|USA|
MG848418.1|North America|USA|
MG849704.1|North America|USA|
MG849245.1|North America|USA|
MG850753.1|North America|USA|
MG849400.1|North America|USA|
MG850615.1|North America|USA|
MG849934.1|North America|USA|
MG848311.1|North America|USA|
MG850730.1|North America|USA|
MG850811.1|North America|USA|
MG848854.1|North America|USA|
MG849023.1|North America|USA|
MG849181.1|North America|USA|
MG849489.1|North America|USA|
MG848605.1|North America|USA|
MG848832.1|North America|USA|
MG848876.1|North America|USA|
MG850325.1|North America|USA|
KP212157.1|South America|Brazil|2011-08-11
KP874716.1|South America|Brazil|
MG848359.1|North America|USA|
MG848423.1|North America|USA|
MG848593.1|North America|USA|
OP728444.1|Europe|United Kingdom|2018
MG848157.1|North America|USA|
MG849253.1|North America|USA|
MG849381.1|North America|USA|
MG848285.1|North America|USA|
MG848757.1|North America|USA|
MG848763.1|North America|USA|
MG848829.1|North America|USA|
MG849385.1|North America|USA|
MG850352.1|North America|USA|
MG849987.1|North America|USA|
MG850042.1|North America|USA|
MG850645.1|North America|USA|
MG848354.1|North America|USA|
MG850022.1|North America|USA|
MG848914.1|North America|USA|

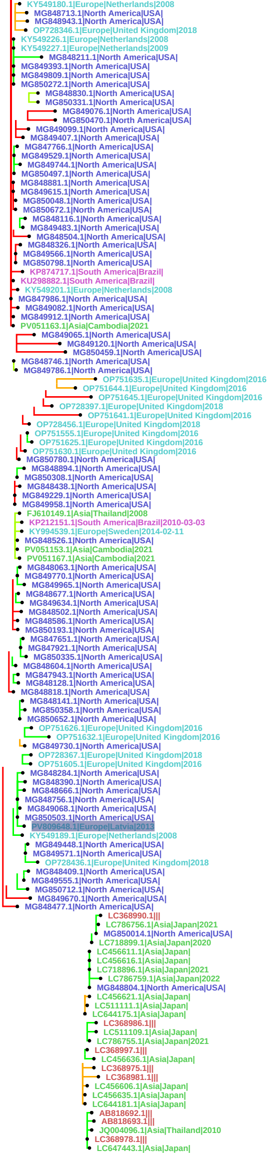

KY549180.1|Europe|Netherlands|2008
MG848713.1|North America|USA|
MG848943.1|North America|USA|
OP728346.1|Europe|United Kingdom|2018
KY549226.1|Europe|Netherlands|2008
KY549227.1|Europe|Netherlands|2009
MG848211.1|North America|USA|
MG849393.1|North America|USA|
MG849809.1|North America|USA|
MG850272.1|North America|USA|
MG848830.1|North America|USA|
MG850331.1|North America|USA|
MG849076.1|North America|USA|
MG850470.1|North America|USA|
MG849099.1|North America|USA|
MG849407.1|North America|USA|
MG847766.1|North America|USA|
MG849529.1|North America|USA|
MG849744.1|North America|USA|
MG850497.1|North America|USA|
MG848881.1|North America|USA|
MG849615.1|North America|USA|
MG850048.1|North America|USA|
MG850672.1|North America|USA|
MG848116.1|North America|USA|
MG849483.1|North America|USA|
MG848504.1|North America|USA|
MG848326.1|North America|USA|
MG849566.1|North America|USA|
MG850798.1|North America|USA|
KP874717.1|South America|Brazil|
KU298882.1|South America|Brazil|
KY549201.1|Europe|Netherlands|2008
MG847986.1|North America|USA|
MG849082.1|North America|USA|
MG849912.1|North America|USA|
PV051163.1|Asia|Cambodia|2021
MG849065.1|North America|USA|
MG849120.1|North America|USA|
MG850459.1|North America|USA|
MG848746.1|North America|USA|
MG849786.1|North America|USA|
OP751635.1|Europe|United Kingdom|2016
OP751644.1|Europe|United Kingdom|2016
OP751645.1|Europe|United Kingdom|2016
OP728397.1|Europe|United Kingdom|2018
OP751641.1|Europe|United Kingdom|2016
OP728456.1|Europe|United Kingdom|2018
OP751555.1|Europe|United Kingdom|2016
OP751625.1|Europe|United Kingdom|2016
OP751630.1|Europe|United Kingdom|2016
MG850780.1|North America|USA|
MG848894.1|North America|USA|
MG850308.1|North America|USA|
MG848438.1|North America|USA|
MG849229.1|North America|USA|
MG849958.1|North America|USA|
FJ610149.1|Asia|Thailand|2008
KP212151.1|South America|Brazil|2010-03-03
KY994539.1|Europe|Sweden|2014-02-11
MG848526.1|North America|USA|
PV051153.1|Asia|Cambodia|2021
PV051167.1|Asia|Cambodia|2021
MG848063.1|North America|USA|
MG849770.1|North America|USA|
MG849965.1|North America|USA|
MG848677.1|North America|USA|
MG849634.1|North America|USA|
MG848502.1|North America|USA|
MG848586.1|North America|USA|
MG850193.1|North America|USA|
MG847651.1|North America|USA|
MG847921.1|North America|USA|
MG850335.1|North America|USA|
MG848604.1|North America|USA|
MG847943.1|North America|USA|
MG848128.1|North America|USA|
MG848818.1|North America|USA|
MG848141.1|North America|USA|
MG850358.1|North America|USA|
MG850652.1|North America|USA|
OP751626.1|Europe|United Kingdom|2016
OP751632.1|Europe|United Kingdom|2016
MG849730.1|North America|USA|
OP728367.1|Europe|United Kingdom|2018
OP751605.1|Europe|United Kingdom|2016
MG848284.1|North America|USA|
MG848390.1|North America|USA|
MG848666.1|North America|USA|
MG848756.1|North America|USA|
MG849068.1|North America|USA|
MG850503.1|North America|USA|
PV809648.1|Europe|Latvia|2013
KY549189.1|Europe|Netherlands|2008
MG849448.1|North America|USA|
MG849571.1|North America|USA|
OP728436.1|Europe|United Kingdom|2018
MG848409.1|North America|USA|
MG849555.1|North America|USA|
MG850712.1|North America|USA|
MG849670.1|North America|USA|
MG848477.1|North America|USA|
LC368990.1|||
LC786756.1|Asia|Japan|2021
MG850014.1|North America|USA|
LC718899.1|Asia|Japan|2020
LC456611.1|Asia|Japan|
LC456616.1|Asia|Japan|
LC718896.1|Asia|Japan|2021
LC786759.1|Asia|Japan|2022
MG848804.1|North America|USA|
LC456621.1|Asia|Japan|
LC511111.1|Asia|Japan|
LC644175.1|Asia|Japan|
LC368986.1|||
LC511109.1|Asia|Japan|
LC786755.1|Asia|Japan|2021
LC368997.1|||
LC456636.1|Asia|Japan|
LC368975.1|||
LC368981.1|||
LC456606.1|Asia|Japan|
LC456635.1|Asia|Japan|
LC644181.1|Asia|Japan|
AB818692.1|||
AB818693.1|||
JQ004096.1|Asia|Thailand|2010
LC368978.1|||
LC647443.1|Asia|Japan|

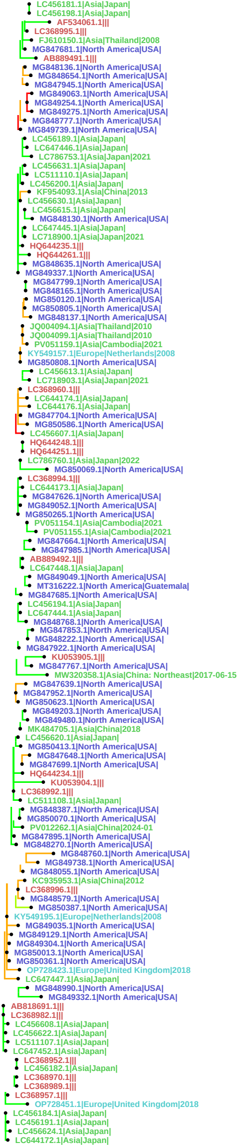

LC456181.1|Asia|Japan|
LC456198.1|Asia|Japan|
AF534061.1|||
LC368995.1|||
FJ610150.1|Asia|Thailand|2008
MG847681.1|North America|USA|
AB889491.1|||
MG848136.1|North America|USA|
MG848654.1|North America|USA|
MG847945.1|North America|USA|
MG849063.1|North America|USA|
MG849254.1|North America|USA|
MG849275.1|North America|USA|
MG848777.1|North America|USA|
MG849739.1|North America|USA|
LC456189.1|Asia|Japan|
LC647446.1|Asia|Japan|
LC786753.1|Asia|Japan|2021
LC456631.1|Asia|Japan|
LC511110.1|Asia|Japan|
LC456200.1|Asia|Japan|
KF954093.1|Asia|China|2013
LC456630.1|Asia|Japan|
LC456615.1|Asia|Japan|
MG848130.1|North America|USA|
LC647445.1|Asia|Japan|
LC718900.1|Asia|Japan|2021
HQ644235.1|||
HQ644261.1|||
MG848635.1|North America|USA|
MG849337.1|North America|USA|
MG847799.1|North America|USA|
MG848165.1|North America|USA|
MG850120.1|North America|USA|
MG850805.1|North America|USA|
MG848137.1|North America|USA|
JQ004094.1|Asia|Thailand|2010
JQ004099.1|Asia|Thailand|2010
PV051159.1|Asia|Cambodia|2021
KY549157.1|Europe|Netherlands|2008
MG850808.1|North America|USA|
LC456613.1|Asia|Japan|
LC718903.1|Asia|Japan|2021
LC368960.1|||
LC644174.1|Asia|Japan|
LC644176.1|Asia|Japan|
MG847704.1|North America|USA|
MG850586.1|North America|USA|
LC456607.1|Asia|Japan|
HQ644248.1|||
HQ644251.1|||
LC786760.1|Asia|Japan|2022
MG850069.1|North America|USA|
LC368994.1|||
LC644173.1|Asia|Japan|
MG847626.1|North America|USA|
MG849052.1|North America|USA|
MG850265.1|North America|USA|
PV051154.1|Asia|Cambodia|2021
PV051155.1|Asia|Cambodia|2021
MG847664.1|North America|USA|
MG847985.1|North America|USA|
AB889492.1|||
LC647448.1|Asia|Japan|
MG849049.1|North America|USA|
MT316222.1|North America|Guatemala|
MG847685.1|North America|USA|
LC456194.1|Asia|Japan|
LC647444.1|Asia|Japan|
MG848768.1|North America|USA|
MG847853.1|North America|USA|
MG848222.1|North America|USA|
MG847922.1|North America|USA|
KU053905.1|||
MG847767.1|North America|USA|
MW320358.1|Asia|China: Northeast|2017-06-15
MG847639.1|North America|USA|
MG847952.1|North America|USA|
MG850623.1|North America|USA|
MG849203.1|North America|USA|
MG849480.1|North America|USA|
MK484705.1|Asia|China|2018
LC456620.1|Asia|Japan|
MG850413.1|North America|USA|
MG847648.1|North America|USA|
MG847699.1|North America|USA|
HQ644234.1|||
KU053904.1|||
LC368992.1|||
LC511108.1|Asia|Japan|
MG848387.1|North America|USA|
MG850070.1|North America|USA|
PV012262.1|Asia|China|2024-01
MG847895.1|North America|USA|
MG848270.1|North America|USA|
MG848760.1|North America|USA|
MG849738.1|North America|USA|
MG848055.1|North America|USA|
KC935953.1|Asia|China|2012
LC368996.1|||
MG848579.1|North America|USA|
MG850387.1|North America|USA|
KY549195.1|Europe|Netherlands|2008
MG849035.1|North America|USA|
MG849129.1|North America|USA|
MG849304.1|North America|USA|
MG850013.1|North America|USA|
MG850361.1|North America|USA|
OP728423.1|Europe|United Kingdom|2018
LC647447.1|Asia|Japan|
MG848990.1|North America|USA|
MG849332.1|North America|USA|
AB818691.1|||
LC368982.1|||
LC456608.1|Asia|Japan|
LC456622.1|Asia|Japan|
LC511107.1|Asia|Japan|
LC647452.1|Asia|Japan|
LC368952.1|||
LC456182.1|Asia|Japan|
LC368970.1|||
LC368989.1|||
LC368957.1|||
OP728451.1|Europe|United Kingdom|2018
LC456184.1|Asia|Japan|
LC456191.1|Asia|Japan|
LC456624.1|Asia|Japan|
LC644172.1|Asia|Japan|

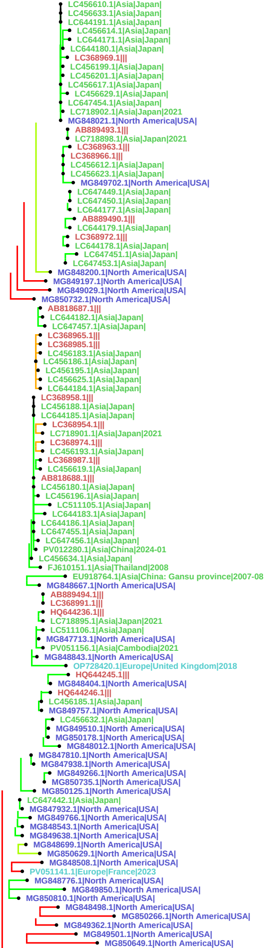
LC456610.1|Asia|Japan|
LC456633.1|Asia|Japan|
LC644191.1|Asia|Japan|
LC456614.1|Asia|Japan|
LC644171.1|Asia|Japan|
LC644180.1|Asia|Japan|
LC368969.1|||
LC456199.1|Asia|Japan|
LC456201.1|Asia|Japan|
LC456617.1|Asia|Japan|
LC456629.1|Asia|Japan|
LC647454.1|Asia|Japan|
LC718902.1|Asia|Japan|2021
MG848021.1|North America|USA|
AB889493.1|||
LC718898.1|Asia|Japan|2021
LC368963.1|||
LC368966.1|||
LC456612.1|Asia|Japan|
LC456623.1|Asia|Japan|
MG849702.1|North America|USA|
LC647449.1|Asia|Japan|
LC647450.1|Asia|Japan|
LC644177.1|Asia|Japan|
AB889490.1|||
LC644179.1|Asia|Japan|
LC368972.1|||
LC644178.1|Asia|Japan|
LC647451.1|Asia|Japan|
LC647453.1|Asia|Japan|
MG848200.1|North America|USA|
MG849197.1|North America|USA|
MG849029.1|North America|USA|
MG850732.1|North America|USA|
AB818687.1|||
LC644182.1|Asia|Japan|
LC647457.1|Asia|Japan|
LC368965.1|||
LC368985.1|||
LC456183.1|Asia|Japan|
LC456186.1|Asia|Japan|
LC456195.1|Asia|Japan|
LC456625.1|Asia|Japan|
LC644184.1|Asia|Japan|
LC368958.1|||
LC456188.1|Asia|Japan|
LC644185.1|Asia|Japan|
LC368954.1|||
LC718901.1|Asia|Japan|2021
LC368974.1|||
LC456193.1|Asia|Japan|
LC368987.1|||
LC456619.1|Asia|Japan|
AB818688.1|||
LC456180.1|Asia|Japan|
LC456196.1|Asia|Japan|
LC511105.1|Asia|Japan|
LC644183.1|Asia|Japan|
LC644186.1|Asia|Japan|
LC647455.1|Asia|Japan|
LC647456.1|Asia|Japan|
PV012280.1|Asia|China|2024-01
LC456634.1|Asia|Japan|
FJ610151.1|Asia|Thailand|2008
EU918764.1|Asia|China: Gansu province|2007-08
MG848667.1|North America|USA|
AB889494.1|||
LC368991.1|||
HQ644236.1|||
LC718895.1|Asia|Japan|2021
LC511106.1|Asia|Japan|
MG847713.1|North America|USA|
PV051156.1|Asia|Cambodia|2021
MG848843.1|North America|USA|
OP728420.1|Europe|United Kingdom|2018
HQ644245.1|||
MG848404.1|North America|USA|
HQ644246.1|||
LC456185.1|Asia|Japan|
MG849757.1|North America|USA|
LC456632.1|Asia|Japan|
MG849510.1|North America|USA|
MG850178.1|North America|USA|
MG848012.1|North America|USA|
MG847810.1|North America|USA|
MG847938.1|North America|USA|
MG849266.1|North America|USA|
MG850735.1|North America|USA|
MG850125.1|North America|USA|
LC647442.1|Asia|Japan|
MG847932.1|North America|USA|
MG849766.1|North America|USA|
MG848543.1|North America|USA|
MG849638.1|North America|USA|
MG848699.1|North America|USA|
MG850629.1|North America|USA|
MG848508.1|North America|USA|
PV051141.1|Europe|France|2023
MG848776.1|North America|USA|
MG849850.1|North America|USA|
MG850810.1|North America|USA|
MG848498.1|North America|USA|
MG850266.1|North America|USA|
MG849362.1|North America|USA|
MG849501.1|North America|USA|
MG850649.1|North America|USA|

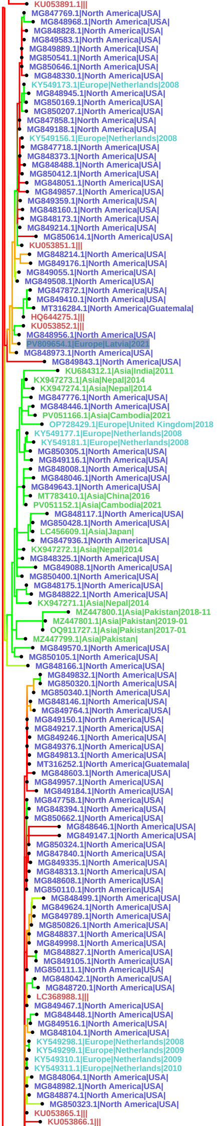
KU053891.1|||
MG847769.1|North America|USA|
MG848968.1|North America|USA|
MG848828.1|North America|USA|
MG849583.1|North America|USA|
MG849889.1|North America|USA|
MG850541.1|North America|USA|
MG850646.1|North America|USA|
MG848330.1|North America|USA|
KY549173.1|Europe|Netherlands|2008
MG848945.1|North America|USA|
MG850169.1|North America|USA|
MG850207.1|North America|USA|
MG847858.1|North America|USA|
MG849188.1|North America|USA|
KY549156.1|Europe|Netherlands|2008
MG847718.1|North America|USA|
MG848373.1|North America|USA|
MG848488.1|North America|USA|
MG850412.1|North America|USA|
MG848051.1|North America|USA|
MG849857.1|North America|USA|
MG849359.1|North America|USA|
MG848160.1|North America|USA|
MG848173.1|North America|USA|
MG849214.1|North America|USA|
MG850614.1|North America|USA|
KU053851.1|||
MG848214.1|North America|USA|
MG849176.1|North America|USA|
MG849055.1|North America|USA|
MG849508.1|North America|USA|
MG847872.1|North America|USA|
MG849410.1|North America|USA|
MT316284.1|North America|Guatemala|
HQ644275.1|||
KU053852.1|||
MG848956.1|North America|USA|
PV809654.1|Europe|Latvia|2021
MG848973.1|North America|USA|
MG849843.1|North America|USA|
KU684312.1|Asia|India|2011
KX947273.1|Asia|Nepal|2014
KX947274.1|Asia|Nepal|2014
MG847776.1|North America|USA|
MG848446.1|North America|USA|
PV051166.1|Asia|Cambodia|2021
OP728429.1|Europe|United Kingdom|2018
KY549177.1|Europe|Netherlands|2008
KY549181.1|Europe|Netherlands|2008
MG850305.1|North America|USA|
MG849116.1|North America|USA|
MG848008.1|North America|USA|
MG848046.1|North America|USA|
MG849643.1|North America|USA|
MT783410.1|Asia|China|2016
PV051152.1|Asia|Cambodia|2021
MG848117.1|North America|USA|
MG850428.1|North America|USA|
LC456609.1|Asia|Japan|
MG847936.1|North America|USA|
KX947272.1|Asia|Nepal|2014
MG848325.1|North America|USA|
MG849088.1|North America|USA|
MG850400.1|North America|USA|
MG848175.1|North America|USA|
MG848822.1|North America|USA|
KX947271.1|Asia|Nepal|2014
MZ447800.1|Asia|Pakistan|2018-11
MZ447801.1|Asia|Pakistan|2019-01
OQ911727.1|Asia|Pakistan|2017-01
MZ447799.1|Asia|Pakistan|
MG849570.1|North America|USA|
MG850105.1|North America|USA|
MG848166.1|North America|USA|
MG849832.1|North America|USA|
MG850320.1|North America|USA|
MG850340.1|North America|USA|
MG848146.1|North America|USA|
MG849764.1|North America|USA|
MG849150.1|North America|USA|
MG849217.1|North America|USA|
MG849246.1|North America|USA|
MG849376.1|North America|USA|
MG849813.1|North America|USA|
MT316252.1|North America|Guatemala|
MG848603.1|North America|USA|
MG849957.1|North America|USA|
MG849184.1|North America|USA|
MG847758.1|North America|USA|
MG848394.1|North America|USA|
MG850662.1|North America|USA|
MG848646.1|North America|USA|
MG849147.1|North America|USA|
MG850324.1|North America|USA|
MG847840.1|North America|USA|
MG849335.1|North America|USA|
MG848313.1|North America|USA|
MG848608.1|North America|USA|
MG850110.1|North America|USA|
MG848499.1|North America|USA|
MG849624.1|North America|USA|
MG849789.1|North America|USA|
MG850826.1|North America|USA|
MG848837.1|North America|USA|
MG849998.1|North America|USA|
MG848827.1|North America|USA|
MG849105.1|North America|USA|
MG850111.1|North America|USA|
MG848042.1|North America|USA|
MG848720.1|North America|USA|
LC368988.1|||
MG849467.1|North America|USA|
MG848448.1|North America|USA|
MG849516.1|North America|USA|
MG848104.1|North America|USA|
KY549298.1|Europe|Netherlands|2008
KY549299.1|Europe|Netherlands|2009
KY549310.1|Europe|Netherlands|2009
KY549311.1|Europe|Netherlands|2010
MG848064.1|North America|USA|
MG848982.1|North America|USA|
MG848874.1|North America|USA|
MG850323.1|North America|USA|
KU053865.1|||
KU053866.1|||

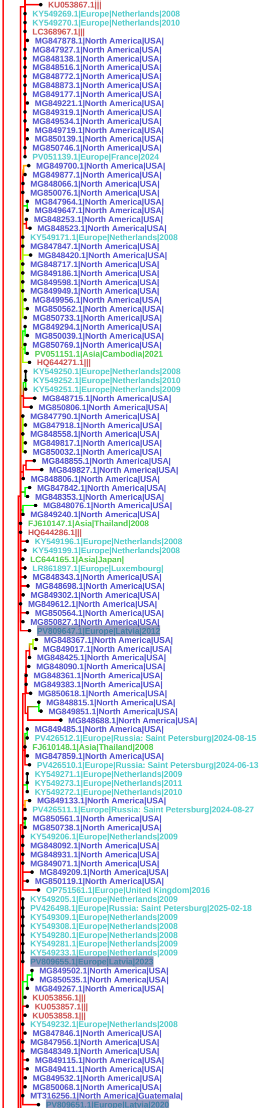
KU053867.1|||
KY549269.1|Europe|Netherlands|2008
KY549270.1|Europe|Netherlands|2010
LC368967.1|||
MG847878.1|North America|USA|
MG847927.1|North America|USA|
MG848138.1|North America|USA|
MG848516.1|North America|USA|
MG848772.1|North America|USA|
MG848873.1|North America|USA|
MG849177.1|North America|USA|
MG849221.1|North America|USA|
MG849319.1|North America|USA|
MG849534.1|North America|USA|
MG849719.1|North America|USA|
MG850139.1|North America|USA|
MG850746.1|North America|USA|
PV051139.1|Europe|France|2024
MG849700.1|North America|USA|
MG849877.1|North America|USA|
MG848066.1|North America|USA|
MG850076.1|North America|USA|
MG847964.1|North America|USA|
MG849647.1|North America|USA|
MG848253.1|North America|USA|
MG848523.1|North America|USA|
KY549171.1|Europe|Netherlands|2008
MG847847.1|North America|USA|
MG848420.1|North America|USA|
MG848717.1|North America|USA|
MG849186.1|North America|USA|
MG849598.1|North America|USA|
MG849949.1|North America|USA|
MG849956.1|North America|USA|
MG850562.1|North America|USA|
MG850733.1|North America|USA|
MG849294.1|North America|USA|
MG850039.1|North America|USA|
MG850769.1|North America|USA|
PV051151.1|Asia|Cambodia|2021
HQ644271.1|||
KY549250.1|Europe|Netherlands|2008
KY549252.1|Europe|Netherlands|2010
KY549251.1|Europe|Netherlands|2009
MG848715.1|North America|USA|
MG850806.1|North America|USA|
MG847790.1|North America|USA|
MG847918.1|North America|USA|
MG848558.1|North America|USA|
MG849817.1|North America|USA|
MG850032.1|North America|USA|
MG848855.1|North America|USA|
MG849827.1|North America|USA|
MG848806.1|North America|USA|
MG847842.1|North America|USA|
MG848353.1|North America|USA|
MG848076.1|North America|USA|
MG849240.1|North America|USA|
FJ610147.1|Asia|Thailand|2008
HQ644286.1|||
KY549196.1|Europe|Netherlands|2008
KY549199.1|Europe|Netherlands|2008
LC644165.1|Asia|Japan|
LR861897.1|Europe|Luxembourg|
MG848343.1|North America|USA|
MG848698.1|North America|USA|
MG849302.1|North America|USA|
MG849612.1|North America|USA|
MG850564.1|North America|USA|
MG850827.1|North America|USA|
PV809647.1|Europe|Latvia|2012
MG848367.1|North America|USA|
MG849017.1|North America|USA|
MG848425.1|North America|USA|
MG848090.1|North America|USA|
MG848361.1|North America|USA|
MG849383.1|North America|USA|
MG850618.1|North America|USA|
MG848815.1|North America|USA|
MG849851.1|North America|USA|
MG848688.1|North America|USA|
MG849485.1|North America|USA|
PV426512.1|Europe|Russia: Saint Petersburg|2024-08-15
FJ610148.1|Asia|Thailand|2008
MG847859.1|North America|USA|
PV426510.1|Europe|Russia: Saint Petersburg|2024-06-13
KY549271.1|Europe|Netherlands|2009
KY549273.1|Europe|Netherlands|2011
KY549272.1|Europe|Netherlands|2010
MG849133.1|North America|USA|
PV426511.1|Europe|Russia: Saint Petersburg|2024-08-27
MG850561.1|North America|USA|
MG850738.1|North America|USA|
KY549206.1|Europe|Netherlands|2009
MG848092.1|North America|USA|
MG848931.1|North America|USA|
MG849071.1|North America|USA|
MG849209.1|North America|USA|
MG850119.1|North America|USA|
OP751561.1|Europe|United Kingdom|2016
KY549205.1|Europe|Netherlands|2009
PV426498.1|Europe|Russia: Saint Petersburg|2025-02-18
KY549309.1|Europe|Netherlands|2009
KY549308.1|Europe|Netherlands|2008
KY549280.1|Europe|Netherlands|2008
KY549281.1|Europe|Netherlands|2009
KY549233.1|Europe|Netherlands|2009
PV809655.1|Europe|Latvia|2023
MG849502.1|North America|USA|
MG850535.1|North America|USA|
MG849267.1|North America|USA|
KU053856.1|||
KU053857.1|||
KU053858.1|||
KY549232.1|Europe|Netherlands|2008
MG847846.1|North America|USA|
MG847956.1|North America|USA|
MG848349.1|North America|USA|
MG849115.1|North America|USA|
MG849411.1|North America|USA|
MG849532.1|North America|USA|
MG850068.1|North America|USA|
MT316256.1|North America|Guatemala|
PV809651.1|Europe|Latvia|2020

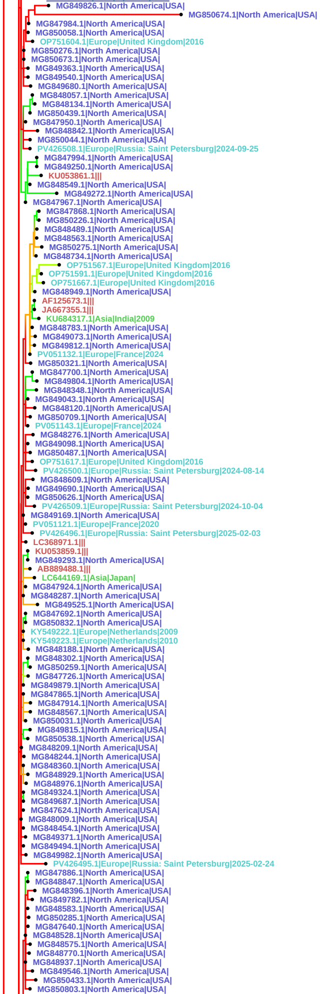
MG849826.1|North America|USA|
MG850674.1|North America|USA|
MG847984.1|North America|USA|
MG850058.1|North America|USA|
OP751604.1|Europe|United Kingdom|2016
MG850276.1|North America|USA|
MG850673.1|North America|USA|
MG849363.1|North America|USA|
MG849540.1|North America|USA|
MG849680.1|North America|USA|
MG848057.1|North America|USA|
MG848134.1|North America|USA|
MG850439.1|North America|USA|
MG847950.1|North America|USA|
MG848842.1|North America|USA|
MG850044.1|North America|USA|
PV426508.1|Europe|Russia: Saint Petersburg|2024-09-25
MG847994.1|North America|USA|
MG849250.1|North America|USA|
KU053861.1|||
MG848549.1|North America|USA|
MG849272.1|North America|USA|
MG847967.1|North America|USA|
MG847868.1|North America|USA|
MG850226.1|North America|USA|
MG848489.1|North America|USA|
MG848563.1|North America|USA|
MG850275.1|North America|USA|
MG848734.1|North America|USA|
OP751567.1|Europe|United Kingdom|2016
OP751591.1|Europe|United Kingdom|2016
OP751667.1|Europe|United Kingdom|2016
MG848949.1|North America|USA|
AF125673.1|||
JA667355.1|||
KU684317.1|Asia|India|2009
MG848783.1|North America|USA|
MG849073.1|North America|USA|
MG849812.1|North America|USA|
PV051132.1|Europe|France|2024
MG850321.1|North America|USA|
MG847700.1|North America|USA|
MG849804.1|North America|USA|
MG848348.1|North America|USA|
MG849043.1|North America|USA|
MG848120.1|North America|USA|
MG850709.1|North America|USA|
PV051143.1|Europe|France|2024
MG848276.1|North America|USA|
MG849098.1|North America|USA|
MG850487.1|North America|USA|
OP751617.1|Europe|United Kingdom|2016
PV426500.1|Europe|Russia: Saint Petersburg|2024-08-14
MG848609.1|North America|USA|
MG849690.1|North America|USA|
MG850626.1|North America|USA|
PV426509.1|Europe|Russia: Saint Petersburg|2024-10-04
MG849169.1|North America|USA|
PV051121.1|Europe|France|2020
PV426496.1|Europe|Russia: Saint Petersburg|2025-02-03
LC368971.1|||
KU053859.1|||
MG849293.1|North America|USA|
AB889488.1|||
LC644169.1|Asia|Japan|
MG847924.1|North America|USA|
MG848287.1|North America|USA|
MG849525.1|North America|USA|
MG847692.1|North America|USA|
MG850832.1|North America|USA|
KY549222.1|Europe|Netherlands|2009
KY549223.1|Europe|Netherlands|2010
MG848188.1|North America|USA|
MG848302.1|North America|USA|
MG850259.1|North America|USA|
MG847726.1|North America|USA|
MG849879.1|North America|USA|
MG847865.1|North America|USA|
MG847914.1|North America|USA|
MG848567.1|North America|USA|
MG850031.1|North America|USA|
MG849815.1|North America|USA|
MG850538.1|North America|USA|
MG848209.1|North America|USA|
MG848244.1|North America|USA|
MG848360.1|North America|USA|
MG848929.1|North America|USA|
MG848976.1|North America|USA|
MG849324.1|North America|USA|
MG849687.1|North America|USA|
MG847624.1|North America|USA|
MG848009.1|North America|USA|
MG848454.1|North America|USA|
MG849371.1|North America|USA|
MG849494.1|North America|USA|
MG849982.1|North America|USA|
PV426495.1|Europe|Russia: Saint Petersburg|2025-02-24
MG847886.1|North America|USA|
MG848847.1|North America|USA|
MG848396.1|North America|USA|
MG849782.1|North America|USA|
MG848583.1|North America|USA|
MG850285.1|North America|USA|
MG847640.1|North America|USA|
MG848528.1|North America|USA|
MG848575.1|North America|USA|
MG848770.1|North America|USA|
MG848937.1|North America|USA|
MG849546.1|North America|USA|
MG850433.1|North America|USA|
MG850803.1|North America|USA|

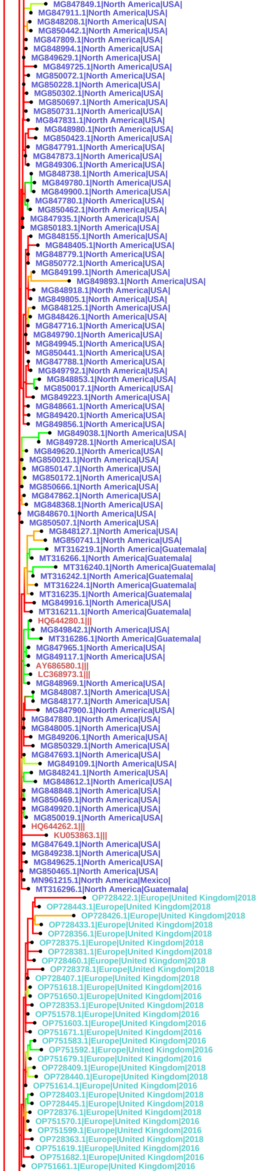
MG847849.1|North America|USA|
MG847911.1|North America|USA|
MG848208.1|North America|USA|
MG850442.1|North America|USA|
MG847809.1|North America|USA|
MG848994.1|North America|USA|
MG849629.1|North America|USA|
MG849725.1|North America|USA|
MG850072.1|North America|USA|
MG850228.1|North America|USA|
MG850302.1|North America|USA|
MG850697.1|North America|USA|
MG850731.1|North America|USA|
MG847831.1|North America|USA|
MG848980.1|North America|USA|
MG850423.1|North America|USA|
MG847791.1|North America|USA|
MG847873.1|North America|USA|
MG849306.1|North America|USA|
MG848738.1|North America|USA|
MG849780.1|North America|USA|
MG849900.1|North America|USA|
MG847780.1|North America|USA|
MG850462.1|North America|USA|
MG847935.1|North America|USA|
MG850183.1|North America|USA|
MG848155.1|North America|USA|
MG848405.1|North America|USA|
MG848779.1|North America|USA|
MG850772.1|North America|USA|
MG849199.1|North America|USA|
MG849893.1|North America|USA|
MG848918.1|North America|USA|
MG849805.1|North America|USA|
MG848125.1|North America|USA|
MG848426.1|North America|USA|
MG847716.1|North America|USA|
MG849790.1|North America|USA|
MG849945.1|North America|USA|
MG850441.1|North America|USA|
MG847788.1|North America|USA|
MG849792.1|North America|USA|
MG848853.1|North America|USA|
MG850017.1|North America|USA|
MG849223.1|North America|USA|
MG848661.1|North America|USA|
MG849420.1|North America|USA|
MG849856.1|North America|USA|
MG849038.1|North America|USA|
MG849728.1|North America|USA|
MG849620.1|North America|USA|
MG850021.1|North America|USA|
MG850147.1|North America|USA|
MG850172.1|North America|USA|
MG850666.1|North America|USA|
MG847862.1|North America|USA|
MG848368.1|North America|USA|
MG848670.1|North America|USA|
MG850507.1|North America|USA|
MG848127.1|North America|USA|
MG850741.1|North America|USA|
MT316219.1|North America|Guatemala|
MT316266.1|North America|Guatemala|
MT316240.1|North America|Guatemala|
MT316242.1|North America|Guatemala|
MT316224.1|North America|Guatemala|
MT316235.1|North America|Guatemala|
MG849916.1|North America|USA|
MT316211.1|North America|Guatemala|
HQ644280.1|||
MG849842.1|North America|USA|
MT316286.1|North America|Guatemala|
MG847965.1|North America|USA|
MG849117.1|North America|USA|
AY686580.1|||
LC368973.1|||
MG848969.1|North America|USA|
MG848087.1|North America|USA|
MG848177.1|North America|USA|
MG847900.1|North America|USA|
MG847880.1|North America|USA|
MG848005.1|North America|USA|
MG849206.1|North America|USA|
MG850329.1|North America|USA|
MG847693.1|North America|USA|
MG849109.1|North America|USA|
MG848241.1|North America|USA|
MG848612.1|North America|USA|
MG848848.1|North America|USA|
MG850469.1|North America|USA|
MG849920.1|North America|USA|
MG850019.1|North America|USA|
HQ644262.1|||
KU053863.1|||
MG847649.1|North America|USA|
MG849238.1|North America|USA|
MG849625.1|North America|USA|
MG850465.1|North America|USA|
MN961215.1|North America|Mexico|
MT316296.1|North America|Guatemala|
OP728422.1|Europe|United Kingdom|2018
OP728443.1|Europe|United Kingdom|2018
OP728426.1|Europe|United Kingdom|2018
OP728433.1|Europe|United Kingdom|2018
OP728356.1|Europe|United Kingdom|2018
OP728375.1|Europe|United Kingdom|2018
OP728381.1|Europe|United Kingdom|2018
OP728460.1|Europe|United Kingdom|2018
OP728378.1|Europe|United Kingdom|2018
OP728407.1|Europe|United Kingdom|2018
OP751618.1|Europe|United Kingdom|2016
OP751650.1|Europe|United Kingdom|2016
OP728353.1|Europe|United Kingdom|2018
OP751578.1|Europe|United Kingdom|2016
OP751603.1|Europe|United Kingdom|2016
OP751671.1|Europe|United Kingdom|2016
OP751583.1|Europe|United Kingdom|2016
OP751592.1|Europe|United Kingdom|2016
OP751679.1|Europe|United Kingdom|2016
OP728409.1|Europe|United Kingdom|2018
OP728440.1|Europe|United Kingdom|2018
OP751614.1|Europe|United Kingdom|2016
OP728403.1|Europe|United Kingdom|2018
OP728445.1|Europe|United Kingdom|2018
OP728376.1|Europe|United Kingdom|2018
OP751570.1|Europe|United Kingdom|2016
OP751599.1|Europe|United Kingdom|2016
OP728363.1|Europe|United Kingdom|2018
OP751619.1|Europe|United Kingdom|2016
OP751682.1|Europe|United Kingdom|2016
OP751661.1|Europe|United Kingdom|2016

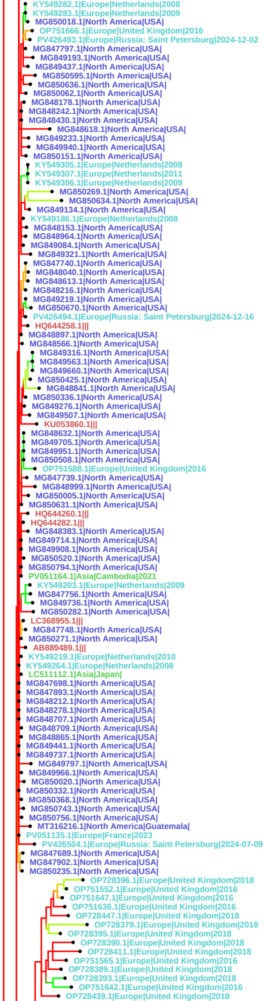
KY549282.1|Europe|Netherlands|2008
KY549283.1|Europe|Netherlands|2009
MG850018.1|North America|USA|
OP751666.1|Europe|United Kingdom|2016
PV426493.1|Europe|Russia: Saint Petersburg|2024-12-02
MG847797.1|North America|USA|
MG849193.1|North America|USA|
MG849437.1|North America|USA|
MG850595.1|North America|USA|
MG850636.1|North America|USA|
MG850062.1|North America|USA|
MG848178.1|North America|USA|
MG848242.1|North America|USA|
MG848430.1|North America|USA|
MG848618.1|North America|USA|
MG849233.1|North America|USA|
MG849940.1|North America|USA|
MG850151.1|North America|USA|
KY549305.1|Europe|Netherlands|2008
KY549307.1|Europe|Netherlands|2011
KY549306.1|Europe|Netherlands|2009
MG850269.1|North America|USA|
MG850634.1|North America|USA|
MG849134.1|North America|USA|
KY549186.1|Europe|Netherlands|2008
MG848153.1|North America|USA|
MG848964.1|North America|USA|
MG849084.1|North America|USA|
MG849321.1|North America|USA|
MG847740.1|North America|USA|
MG848040.1|North America|USA|
MG848613.1|North America|USA|
MG848216.1|North America|USA|
MG849219.1|North America|USA|
MG850670.1|North America|USA|
PV426494.1|Europe|Russia: Saint Petersburg|2024-12-16
HQ644258.1|||
MG848897.1|North America|USA|
MG848566.1|North America|USA|
MG849316.1|North America|USA|
MG849563.1|North America|USA|
MG849660.1|North America|USA|
MG850425.1|North America|USA|
MG848841.1|North America|USA|
MG850336.1|North America|USA|
MG849276.1|North America|USA|
MG849507.1|North America|USA|
KU053860.1|||
MG848632.1|North America|USA|
MG849705.1|North America|USA|
MG849951.1|North America|USA|
MG850508.1|North America|USA|
OP751588.1|Europe|United Kingdom|2016
MG847739.1|North America|USA|
MG848999.1|North America|USA|
MG850005.1|North America|USA|
MG850631.1|North America|USA|
HQ644260.1|||
HQ644282.1|||
MG848383.1|North America|USA|
MG849714.1|North America|USA|
MG849908.1|North America|USA|
MG850520.1|North America|USA|
MG850794.1|North America|USA|
PV051164.1|Asia|Cambodia|2021
KY549203.1|Europe|Netherlands|2009
MG847756.1|North America|USA|
MG849736.1|North America|USA|
MG850282.1|North America|USA|
LC368955.1|||
MG847748.1|North America|USA|
MG850271.1|North America|USA|
AB889489.1|||
KY549219.1|Europe|Netherlands|2010
KY549264.1|Europe|Netherlands|2008
LC511112.1|Asia|Japan|
MG847698.1|North America|USA|
MG847893.1|North America|USA|
MG848212.1|North America|USA|
MG848278.1|North America|USA|
MG848707.1|North America|USA|
MG848709.1|North America|USA|
MG848865.1|North America|USA|
MG849441.1|North America|USA|
MG849737.1|North America|USA|
MG849797.1|North America|USA|
MG849966.1|North America|USA|
MG850020.1|North America|USA|
MG850332.1|North America|USA|
MG850368.1|North America|USA|
MG850743.1|North America|USA|
MG850756.1|North America|USA|
MT316216.1|North America|Guatemala|
PV051135.1|Europe|France|2023
PV426504.1|Europe|Russia: Saint Petersburg|2024-07-09
MG847689.1|North America|USA|
MG847902.1|North America|USA|
MG850235.1|North America|USA|
OP728396.1|Europe|United Kingdom|2018
OP751552.1|Europe|United Kingdom|2016
OP751647.1|Europe|United Kingdom|2016
OP751636.1|Europe|United Kingdom|2016
OP728447.1|Europe|United Kingdom|2018
OP728379.1|Europe|United Kingdom|2018
OP728395.1|Europe|United Kingdom|2018
OP728390.1|Europe|United Kingdom|2018
OP728411.1|Europe|United Kingdom|2018
OP751565.1|Europe|United Kingdom|2016
OP728389.1|Europe|United Kingdom|2018
OP728393.1|Europe|United Kingdom|2018
OP751642.1|Europe|United Kingdom|2016
OP728439.1|Europe|United Kingdom|2018

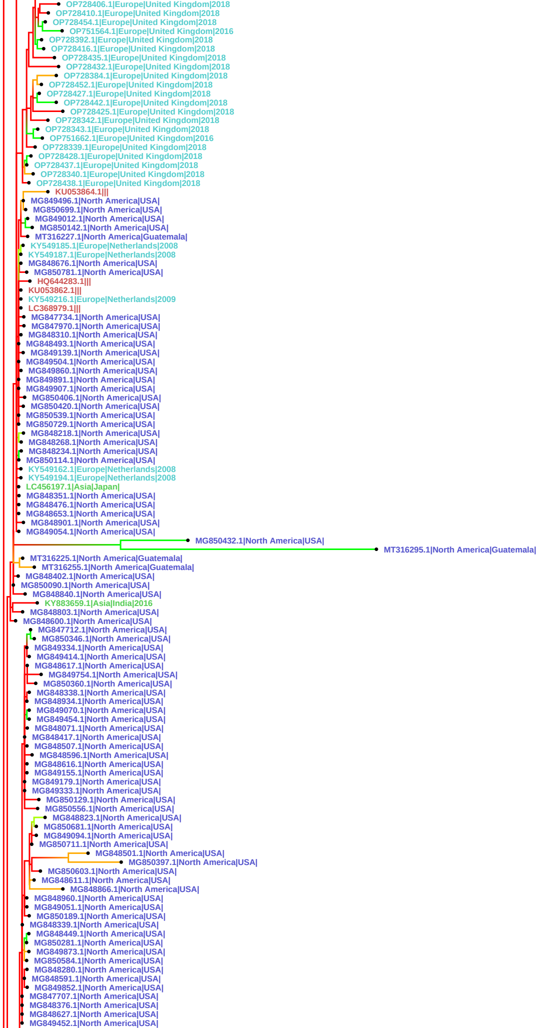
OP728406.1|Europe|United Kingdom|2018
OP728410.1|Europe|United Kingdom|2018
OP728454.1|Europe|United Kingdom|2018
OP751564.1|Europe|United Kingdom|2016
OP728392.1|Europe|United Kingdom|2018
OP728416.1|Europe|United Kingdom|2018
OP728435.1|Europe|United Kingdom|2018
OP728432.1|Europe|United Kingdom|2018
OP728384.1|Europe|United Kingdom|2018
OP728452.1|Europe|United Kingdom|2018
OP728427.1|Europe|United Kingdom|2018
OP728442.1|Europe|United Kingdom|2018
OP728425.1|Europe|United Kingdom|2018
OP728342.1|Europe|United Kingdom|2018
OP728343.1|Europe|United Kingdom|2018
OP751662.1|Europe|United Kingdom|2016
OP728339.1|Europe|United Kingdom|2018
OP728428.1|Europe|United Kingdom|2018
OP728437.1|Europe|United Kingdom|2018
OP728340.1|Europe|United Kingdom|2018
OP728438.1|Europe|United Kingdom|2018
KU053864.1|||
MG849496.1|North America|USA|
MG850699.1|North America|USA|
MG849012.1|North America|USA|
MG850142.1|North America|USA|
MT316227.1|North America|Guatemala|
KY549185.1|Europe|Netherlands|2008
KY549187.1|Europe|Netherlands|2008
MG848676.1|North America|USA|
MG850781.1|North America|USA|
HQ644283.1|||
KU053862.1|||
KY549216.1|Europe|Netherlands|2009
LC368979.1|||
MG847734.1|North America|USA|
MG847970.1|North America|USA|
MG848310.1|North America|USA|
MG848493.1|North America|USA|
MG849139.1|North America|USA|
MG849504.1|North America|USA|
MG849860.1|North America|USA|
MG849891.1|North America|USA|
MG849907.1|North America|USA|
MG850406.1|North America|USA|
MG850420.1|North America|USA|
MG850539.1|North America|USA|
MG850729.1|North America|USA|
MG848218.1|North America|USA|
MG848268.1|North America|USA|
MG848234.1|North America|USA|
MG850114.1|North America|USA|
KY549162.1|Europe|Netherlands|2008
KY549194.1|Europe|Netherlands|2008
LC456197.1|Asia|Japan|
MG848351.1|North America|USA|
MG848476.1|North America|USA|
MG848653.1|North America|USA|
MG848901.1|North America|USA|
MG849054.1|North America|USA|
MG850432.1|North America|USA|
MT316295.1|North America|Guatemala|
MT316225.1|North America|Guatemala|
MT316255.1|North America|Guatemala|
MG848402.1|North America|USA|
MG850090.1|North America|USA|
MG848840.1|North America|USA|
KY883659.1|Asia|India|2016
MG848803.1|North America|USA|
MG848600.1|North America|USA|
MG847712.1|North America|USA|
MG850346.1|North America|USA|
MG849334.1|North America|USA|
MG849414.1|North America|USA|
MG848617.1|North America|USA|
MG849754.1|North America|USA|
MG850360.1|North America|USA|
MG848338.1|North America|USA|
MG848934.1|North America|USA|
MG849070.1|North America|USA|
MG849454.1|North America|USA|
MG848071.1|North America|USA|
MG848417.1|North America|USA|
MG848507.1|North America|USA|
MG848596.1|North America|USA|
MG848616.1|North America|USA|
MG849155.1|North America|USA|
MG849179.1|North America|USA|
MG849333.1|North America|USA|
MG850129.1|North America|USA|
MG850556.1|North America|USA|
MG848823.1|North America|USA|
MG850681.1|North America|USA|
MG849094.1|North America|USA|
MG850711.1|North America|USA|
MG848501.1|North America|USA|
MG850397.1|North America|USA|
MG850603.1|North America|USA|
MG848611.1|North America|USA|
MG848866.1|North America|USA|
MG848960.1|North America|USA|
MG849051.1|North America|USA|
MG850189.1|North America|USA|
MG848339.1|North America|USA|
MG848449.1|North America|USA|
MG850281.1|North America|USA|
MG849873.1|North America|USA|
MG850584.1|North America|USA|
MG848280.1|North America|USA|
MG848591.1|North America|USA|
MG849852.1|North America|USA|
MG847707.1|North America|USA|
MG848376.1|North America|USA|
MG848627.1|North America|USA|
MG849452.1|North America|USA|

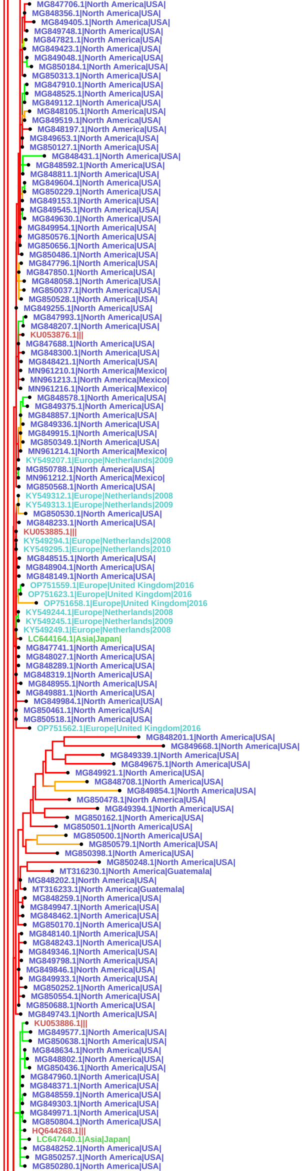
MG847706.1|North America|USA|
MG848356.1|North America|USA|
MG849405.1|North America|USA|
MG849748.1|North America|USA|
MG847821.1|North America|USA|
MG849423.1|North America|USA|
MG849048.1|North America|USA|
MG850184.1|North America|USA|
MG850313.1|North America|USA|
MG847910.1|North America|USA|
MG848525.1|North America|USA|
MG849112.1|North America|USA|
MG848105.1|North America|USA|
MG849519.1|North America|USA|
MG848197.1|North America|USA|
MG849653.1|North America|USA|
MG850127.1|North America|USA|
MG848431.1|North America|USA|
MG848592.1|North America|USA|
MG848811.1|North America|USA|
MG849604.1|North America|USA|
MG850229.1|North America|USA|
MG849153.1|North America|USA|
MG849545.1|North America|USA|
MG849630.1|North America|USA|
MG849954.1|North America|USA|
MG850576.1|North America|USA|
MG850656.1|North America|USA|
MG850486.1|North America|USA|
MG847796.1|North America|USA|
MG847850.1|North America|USA|
MG848058.1|North America|USA|
MG850037.1|North America|USA|
MG850528.1|North America|USA|
MG849255.1|North America|USA|
MG847993.1|North America|USA|
MG848207.1|North America|USA|
KU053876.1|||
MG847688.1|North America|USA|
MG848300.1|North America|USA|
MG848421.1|North America|USA|
MN961210.1|North America|Mexico|
MN961213.1|North America|Mexico|
MN961216.1|North America|Mexico|
MG848578.1|North America|USA|
MG849375.1|North America|USA|
MG848857.1|North America|USA|
MG849336.1|North America|USA|
MG849915.1|North America|USA|
MG850349.1|North America|USA|
MN961214.1|North America|Mexico|
KY549207.1|Europe|Netherlands|2009
MG850788.1|North America|USA|
MN961212.1|North America|Mexico|
MG850568.1|North America|USA|
KY549312.1|Europe|Netherlands|2008
KY549313.1|Europe|Netherlands|2009
MG850530.1|North America|USA|
MG848233.1|North America|USA|
KU053885.1|||
KY549294.1|Europe|Netherlands|2008
KY549295.1|Europe|Netherlands|2010
MG848515.1|North America|USA|
MG848904.1|North America|USA|
MG848149.1|North America|USA|
OP751559.1|Europe|United Kingdom|2016
OP751623.1|Europe|United Kingdom|2016
OP751658.1|Europe|United Kingdom|2016
KY549244.1|Europe|Netherlands|2008
KY549245.1|Europe|Netherlands|2009
KY549249.1|Europe|Netherlands|2008
LC644164.1|Asia|Japan|
MG847741.1|North America|USA|
MG848027.1|North America|USA|
MG848289.1|North America|USA|
MG848319.1|North America|USA|
MG848955.1|North America|USA|
MG849881.1|North America|USA|
MG849984.1|North America|USA|
MG850461.1|North America|USA|
MG850518.1|North America|USA|
OP751562.1|Europe|United Kingdom|2016
MG848201.1|North America|USA|
MG849668.1|North America|USA|
MG849339.1|North America|USA|
MG849675.1|North America|USA|
MG849921.1|North America|USA|
MG848708.1|North America|USA|
MG849854.1|North America|USA|
MG850478.1|North America|USA|
MG849394.1|North America|USA|
MG850162.1|North America|USA|
MG850501.1|North America|USA|
MG850500.1|North America|USA|
MG850579.1|North America|USA|
MG850398.1|North America|USA|
MG850248.1|North America|USA|
MT316230.1|North America|Guatemala|
MG848202.1|North America|USA|
MT316233.1|North America|Guatemala|
MG848259.1|North America|USA|
MG849947.1|North America|USA|
MG848462.1|North America|USA|
MG850170.1|North America|USA|
MG848140.1|North America|USA|
MG848243.1|North America|USA|
MG849346.1|North America|USA|
MG849798.1|North America|USA|
MG849846.1|North America|USA|
MG849933.1|North America|USA|
MG850252.1|North America|USA|
MG850554.1|North America|USA|
MG850688.1|North America|USA|
MG849743.1|North America|USA|
KU053886.1|||
MG849577.1|North America|USA|
MG850638.1|North America|USA|
MG848634.1|North America|USA|
MG848802.1|North America|USA|
MG850436.1|North America|USA|
MG847960.1|North America|USA|
MG848371.1|North America|USA|
MG848559.1|North America|USA|
MG849303.1|North America|USA|
MG849971.1|North America|USA|
MG850804.1|North America|USA|
HQ644268.1|||
LC647440.1|Asia|Japan|
MG848252.1|North America|USA|
MG850257.1|North America|USA|
MG850280.1|North America|USA|

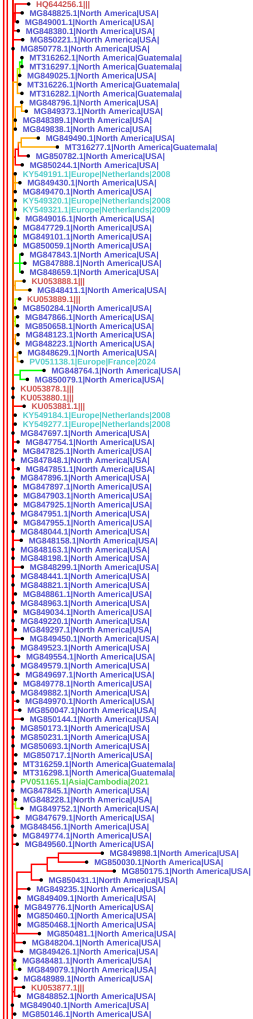
HQ644256.1|||
MG848825.1|North America|USA|
MG849001.1|North America|USA|
MG848380.1|North America|USA|
MG850221.1|North America|USA|
MG850778.1|North America|USA|
MT316262.1|North America|Guatemala|
MT316297.1|North America|Guatemala|
MG849025.1|North America|USA|
MT316226.1|North America|Guatemala|
MT316282.1|North America|Guatemala|
MG848796.1|North America|USA|
MG849373.1|North America|USA|
MG848389.1|North America|USA|
MG849838.1|North America|USA|
MG849490.1|North America|USA|
MT316277.1|North America|Guatemala|
MG850782.1|North America|USA|
MG850244.1|North America|USA|
KY549191.1|Europe|Netherlands|2008
MG849430.1|North America|USA|
MG849470.1|North America|USA|
KY549320.1|Europe|Netherlands|2008
KY549321.1|Europe|Netherlands|2009
MG849016.1|North America|USA|
MG847729.1|North America|USA|
MG849101.1|North America|USA|
MG850059.1|North America|USA|
MG847843.1|North America|USA|
MG847888.1|North America|USA|
MG848659.1|North America|USA|
KU053888.1|||
MG848411.1|North America|USA|
KU053889.1|||
MG850284.1|North America|USA|
MG847866.1|North America|USA|
MG850658.1|North America|USA|
MG848123.1|North America|USA|
MG848223.1|North America|USA|
MG848629.1|North America|USA|
PV051138.1|Europe|France|2024
MG848764.1|North America|USA|
MG850079.1|North America|USA|
KU053878.1|||
KU053880.1|||
KU053881.1|||
KY549184.1|Europe|Netherlands|2008
KY549277.1|Europe|Netherlands|2008
MG847697.1|North America|USA|
MG847754.1|North America|USA|
MG847825.1|North America|USA|
MG847848.1|North America|USA|
MG847851.1|North America|USA|
MG847896.1|North America|USA|
MG847897.1|North America|USA|
MG847903.1|North America|USA|
MG847925.1|North America|USA|
MG847951.1|North America|USA|
MG847955.1|North America|USA|
MG848044.1|North America|USA|
MG848158.1|North America|USA|
MG848163.1|North America|USA|
MG848198.1|North America|USA|
MG848299.1|North America|USA|
MG848441.1|North America|USA|
MG848821.1|North America|USA|
MG848861.1|North America|USA|
MG848963.1|North America|USA|
MG849034.1|North America|USA|
MG849220.1|North America|USA|
MG849297.1|North America|USA|
MG849450.1|North America|USA|
MG849523.1|North America|USA|
MG849554.1|North America|USA|
MG849579.1|North America|USA|
MG849697.1|North America|USA|
MG849778.1|North America|USA|
MG849882.1|North America|USA|
MG849970.1|North America|USA|
MG850047.1|North America|USA|
MG850144.1|North America|USA|
MG850173.1|North America|USA|
MG850231.1|North America|USA|
MG850693.1|North America|USA|
MG850717.1|North America|USA|
MT316259.1|North America|Guatemala|
MT316298.1|North America|Guatemala|
PV051165.1|Asia|Cambodia|2021
MG847845.1|North America|USA|
MG848228.1|North America|USA|
MG849752.1|North America|USA|
MG847679.1|North America|USA|
MG848456.1|North America|USA|
MG849774.1|North America|USA|
MG849560.1|North America|USA|
MG849898.1|North America|USA|
MG850030.1|North America|USA|
MG850175.1|North America|USA|
MG850431.1|North America|USA|
MG849235.1|North America|USA|
MG849409.1|North America|USA|
MG849776.1|North America|USA|
MG850460.1|North America|USA|
MG850468.1|North America|USA|
MG850481.1|North America|USA|
MG848204.1|North America|USA|
MG849426.1|North America|USA|
MG848481.1|North America|USA|
MG849079.1|North America|USA|
MG848989.1|North America|USA|
KU053877.1|||
MG848852.1|North America|USA|
MG849040.1|North America|USA|
MG850146.1|North America|USA|

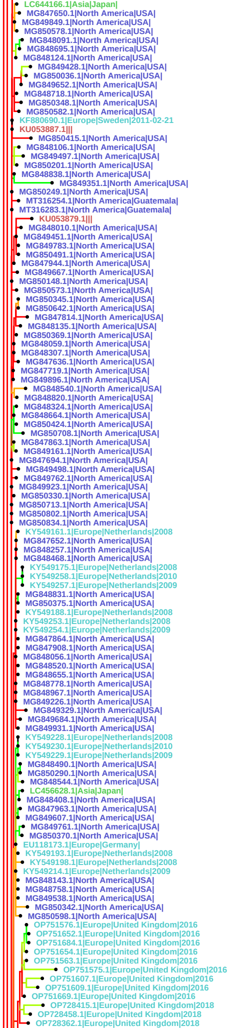
LC644166.1|Asia|Japan|
MG847650.1|North America|USA|
MG849849.1|North America|USA|
MG850578.1|North America|USA|
MG848091.1|North America|USA|
MG848695.1|North America|USA|
MG848124.1|North America|USA|
MG849428.1|North America|USA|
MG850036.1|North America|USA|
MG849652.1|North America|USA|
MG848718.1|North America|USA|
MG850348.1|North America|USA|
MG850582.1|North America|USA|
KF880690.1|Europe|Sweden|2011-02-21
KU053887.1|||
MG850415.1|North America|USA|
MG848106.1|North America|USA|
MG849497.1|North America|USA|
MG850201.1|North America|USA|
MG848838.1|North America|USA|
MG849351.1|North America|USA|
MG850249.1|North America|USA|
MT316254.1|North America|Guatemala|
MT316283.1|North America|Guatemala|
KU053879.1|||
MG848010.1|North America|USA|
MG849451.1|North America|USA|
MG849783.1|North America|USA|
MG850491.1|North America|USA|
MG847944.1|North America|USA|
MG849667.1|North America|USA|
MG850148.1|North America|USA|
MG850573.1|North America|USA|
MG850345.1|North America|USA|
MG850642.1|North America|USA|
MG847814.1|North America|USA|
MG848135.1|North America|USA|
MG850369.1|North America|USA|
MG848059.1|North America|USA|
MG848307.1|North America|USA|
MG847636.1|North America|USA|
MG847719.1|North America|USA|
MG849896.1|North America|USA|
MG848540.1|North America|USA|
MG848820.1|North America|USA|
MG848324.1|North America|USA|
MG848664.1|North America|USA|
MG850424.1|North America|USA|
MG850708.1|North America|USA|
MG847863.1|North America|USA|
MG849161.1|North America|USA|
MG847694.1|North America|USA|
MG849498.1|North America|USA|
MG849762.1|North America|USA|
MG849923.1|North America|USA|
MG850330.1|North America|USA|
MG850713.1|North America|USA|
MG850802.1|North America|USA|
MG850834.1|North America|USA|
KY549161.1|Europe|Netherlands|2008
MG847652.1|North America|USA|
MG848257.1|North America|USA|
MG848468.1|North America|USA|
KY549175.1|Europe|Netherlands|2008
KY549258.1|Europe|Netherlands|2010
KY549257.1|Europe|Netherlands|2009
MG848831.1|North America|USA|
MG850375.1|North America|USA|
KY549188.1|Europe|Netherlands|2008
KY549253.1|Europe|Netherlands|2008
KY549254.1|Europe|Netherlands|2009
MG847864.1|North America|USA|
MG847908.1|North America|USA|
MG848056.1|North America|USA|
MG848520.1|North America|USA|
MG848655.1|North America|USA|
MG848778.1|North America|USA|
MG848967.1|North America|USA|
MG849226.1|North America|USA|
MG849329.1|North America|USA|
MG849684.1|North America|USA|
MG849931.1|North America|USA|
KY549228.1|Europe|Netherlands|2008
KY549230.1|Europe|Netherlands|2010
KY549229.1|Europe|Netherlands|2009
MG848490.1|North America|USA|
MG850290.1|North America|USA|
MG848544.1|North America|USA|
LC456628.1|Asia|Japan|
MG848408.1|North America|USA|
MG847963.1|North America|USA|
MG849607.1|North America|USA|
MG849761.1|North America|USA|
MG850370.1|North America|USA|
EU118173.1|Europe|Germany|
KY549193.1|Europe|Netherlands|2008
KY549198.1|Europe|Netherlands|2008
KY549214.1|Europe|Netherlands|2009
MG848143.1|North America|USA|
MG848758.1|North America|USA|
MG849538.1|North America|USA|
MG850342.1|North America|USA|
MG850598.1|North America|USA|
OP751576.1|Europe|United Kingdom|2016
OP751652.1|Europe|United Kingdom|2016
OP751684.1|Europe|United Kingdom|2016
OP751654.1|Europe|United Kingdom|2016
OP751563.1|Europe|United Kingdom|2016
OP751575.1|Europe|United Kingdom|2016
OP751607.1|Europe|United Kingdom|2016
OP751609.1|Europe|United Kingdom|2016
OP751669.1|Europe|United Kingdom|2016
OP728415.1|Europe|United Kingdom|2018
OP728458.1|Europe|United Kingdom|2018
OP728362.1|Europe|United Kingdom|2018

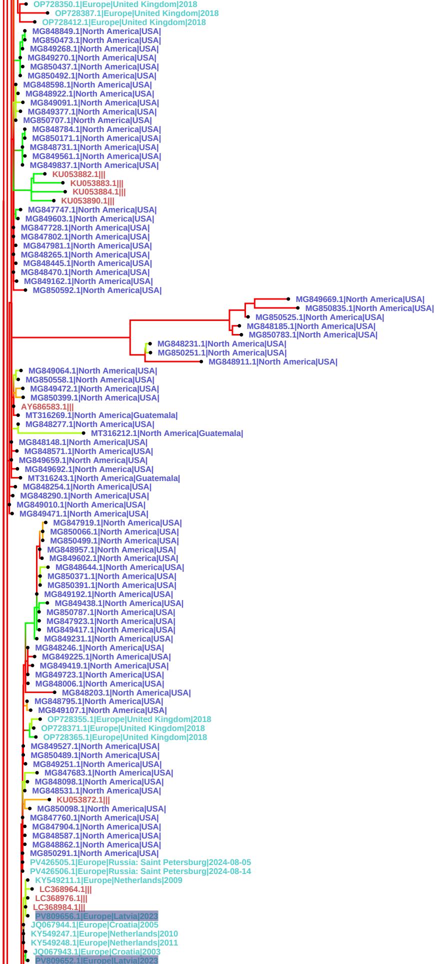

OP728350.1|Europe|United Kingdom|2018
OP728387.1|Europe|United Kingdom|2018
OP728412.1|Europe|United Kingdom|2018
MG848849.1|North America|USA|
MG850473.1|North America|USA|
MG849268.1|North America|USA|
MG849270.1|North America|USA|
MG850437.1|North America|USA|
MG850492.1|North America|USA|
MG848598.1|North America|USA|
MG848922.1|North America|USA|
MG849091.1|North America|USA|
MG849377.1|North America|USA|
MG850707.1|North America|USA|
MG848784.1|North America|USA|
MG850171.1|North America|USA|
MG848731.1|North America|USA|
MG849561.1|North America|USA|
MG849837.1|North America|USA|
KU053882.1|||
KU053883.1|||
KU053884.1|||
KU053890.1|||
MG847747.1|North America|USA|
MG849603.1|North America|USA|
MG847728.1|North America|USA|
MG847802.1|North America|USA|
MG847981.1|North America|USA|
MG848265.1|North America|USA|
MG848445.1|North America|USA|
MG848470.1|North America|USA|
MG849162.1|North America|USA|
MG850592.1|North America|USA|
MG849669.1|North America|USA|
MG850835.1|North America|USA|
MG850525.1|North America|USA|
MG848185.1|North America|USA|
MG850783.1|North America|USA|
MG848231.1|North America|USA|
MG850251.1|North America|USA|
MG848911.1|North America|USA|
MG849064.1|North America|USA|
MG850558.1|North America|USA|
MG849472.1|North America|USA|
MG850399.1|North America|USA|
AY686583.1|||
MT316269.1|North America|Guatemala|
MG848277.1|North America|USA|
MT316212.1|North America|Guatemala|
MG848148.1|North America|USA|
MG848571.1|North America|USA|
MG849659.1|North America|USA|
MG849692.1|North America|USA|
MT316243.1|North America|Guatemala|
MG848254.1|North America|USA|
MG848290.1|North America|USA|
MG849010.1|North America|USA|
MG849471.1|North America|USA|
MG847919.1|North America|USA|
MG850066.1|North America|USA|
MG850499.1|North America|USA|
MG848957.1|North America|USA|
MG849602.1|North America|USA|
MG848644.1|North America|USA|
MG850371.1|North America|USA|
MG850391.1|North America|USA|
MG849192.1|North America|USA|
MG849438.1|North America|USA|
MG850787.1|North America|USA|
MG847923.1|North America|USA|
MG849417.1|North America|USA|
MG849231.1|North America|USA|
MG848246.1|North America|USA|
MG849225.1|North America|USA|
MG849419.1|North America|USA|
MG849723.1|North America|USA|
MG848006.1|North America|USA|
MG848203.1|North America|USA|
MG848795.1|North America|USA|
MG849107.1|North America|USA|
OP728355.1|Europe|United Kingdom|2018
OP728371.1|Europe|United Kingdom|2018
OP728365.1|Europe|United Kingdom|2018
MG849527.1|North America|USA|
MG850489.1|North America|USA|
MG849251.1|North America|USA|
MG847683.1|North America|USA|
MG848098.1|North America|USA|
MG848531.1|North America|USA|
KU053872.1|||
MG850098.1|North America|USA|
MG847760.1|North America|USA|
MG847904.1|North America|USA|
MG848587.1|North America|USA|
MG848862.1|North America|USA|
MG850291.1|North America|USA|
PV426505.1|Europe|Russia: Saint Petersburg|2024-08-05
PV426506.1|Europe|Russia: Saint Petersburg|2024-08-14
KY549211.1|Europe|Netherlands|2009
LC368964.1|||
LC368976.1|||
LC368984.1|||
PV809656.1|Europe|Latvia|2023
JQ067944.1|Europe|Croatia|2005
KY549247.1|Europe|Netherlands|2010
KY549248.1|Europe|Netherlands|2011
JQ067943.1|Europe|Croatia|2003
PV809652.1|Europe|Latvia|2023

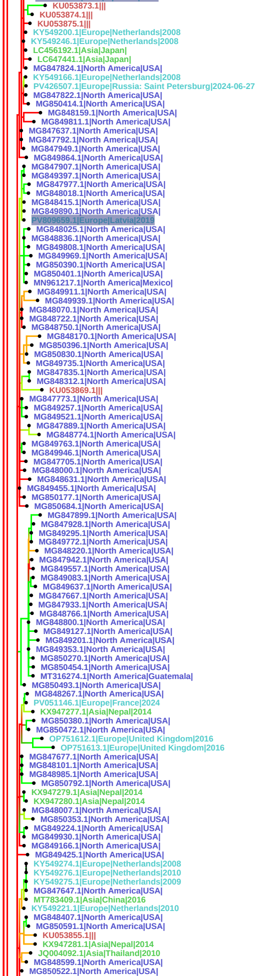
KU053873.1|||
KU053874.1|||
KU053875.1|||
KY549200.1|Europe|Netherlands|2008
KY549246.1|Europe|Netherlands|2008
LC456192.1|Asia|Japan|
LC647441.1|Asia|Japan|
MG847824.1|North America|USA|
KY549166.1|Europe|Netherlands|2008
PV426507.1|Europe|Russia: Saint Petersburg|2024-06-27
MG847822.1|North America|USA|
MG850414.1|North America|USA|
MG848159.1|North America|USA|
MG849811.1|North America|USA|
MG847637.1|North America|USA|
MG847792.1|North America|USA|
MG847949.1|North America|USA|
MG849864.1|North America|USA|
MG847907.1|North America|USA|
MG849397.1|North America|USA|
MG847977.1|North America|USA|
MG848018.1|North America|USA|
MG848415.1|North America|USA|
MG849890.1|North America|USA|
PV809659.1|Europe|Latvia|2019
MG848025.1|North America|USA|
MG848836.1|North America|USA|
MG849808.1|North America|USA|
MG849969.1|North America|USA|
MG850390.1|North America|USA|
MG850401.1|North America|USA|
MN961217.1|North America|Mexico|
MG849911.1|North America|USA|
MG849939.1|North America|USA|
MG848070.1|North America|USA|
MG848722.1|North America|USA|
MG848750.1|North America|USA|
MG848170.1|North America|USA|
MG850396.1|North America|USA|
MG850830.1|North America|USA|
MG849735.1|North America|USA|
MG847835.1|North America|USA|
MG848312.1|North America|USA|
KU053869.1|||
MG847773.1|North America|USA|
MG849257.1|North America|USA|
MG849521.1|North America|USA|
MG847889.1|North America|USA|
MG848774.1|North America|USA|
MG849763.1|North America|USA|
MG849946.1|North America|USA|
MG847705.1|North America|USA|
MG848000.1|North America|USA|
MG848631.1|North America|USA|
MG849455.1|North America|USA|
MG850177.1|North America|USA|
MG850684.1|North America|USA|
MG847899.1|North America|USA|
MG847928.1|North America|USA|
MG849295.1|North America|USA|
MG849772.1|North America|USA|
MG848220.1|North America|USA|
MG847942.1|North America|USA|
MG849557.1|North America|USA|
MG849083.1|North America|USA|
MG849637.1|North America|USA|
MG847667.1|North America|USA|
MG847933.1|North America|USA|
MG848766.1|North America|USA|
MG848800.1|North America|USA|
MG849127.1|North America|USA|
MG849201.1|North America|USA|
MG849353.1|North America|USA|
MG850270.1|North America|USA|
MG850454.1|North America|USA|
MT316274.1|North America|Guatemala|
MG850493.1|North America|USA|
MG848267.1|North America|USA|
PV051146.1|Europe|France|2024
KX947277.1|Asia|Nepal|2014
MG850380.1|North America|USA|
MG850472.1|North America|USA|
OP751612.1|Europe|United Kingdom|2016
OP751613.1|Europe|United Kingdom|2016
MG847677.1|North America|USA|
MG848101.1|North America|USA|
MG848985.1|North America|USA|
MG850792.1|North America|USA|
KX947279.1|Asia|Nepal|2014
KX947280.1|Asia|Nepal|2014
MG848007.1|North America|USA|
MG850353.1|North America|USA|
MG849224.1|North America|USA|
MG849930.1|North America|USA|
MG849166.1|North America|USA|
MG849425.1|North America|USA|
KY549274.1|Europe|Netherlands|2008
KY549276.1|Europe|Netherlands|2010
KY549275.1|Europe|Netherlands|2009
MG847647.1|North America|USA|
MT783409.1|Asia|China|2016
KY549221.1|Europe|Netherlands|2010
MG848407.1|North America|USA|
MG850591.1|North America|USA|
KU053855.1|||
KX947281.1|Asia|Nepal|2014
JQ004092.1|Asia|Thailand|2010
MG848599.1|North America|USA|
MG850522.1|North America|USA|

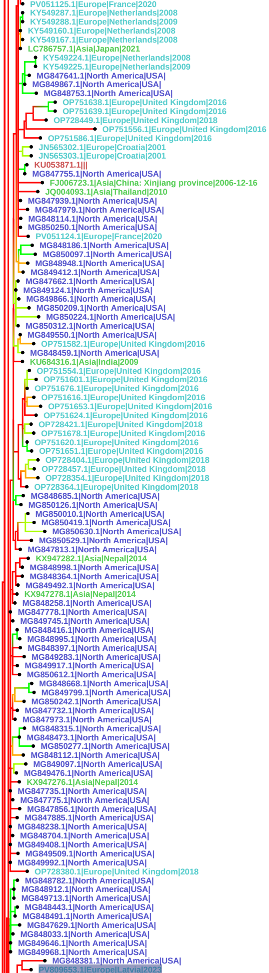

PV051125.1|Europe|France|2020
KY549287.1|Europe|Netherlands|2008
KY549288.1|Europe|Netherlands|2009
KY549160.1|Europe|Netherlands|2008
KY549167.1|Europe|Netherlands|2008
LC786757.1|Asia|Japan|2021
KY549224.1|Europe|Netherlands|2008
KY549225.1|Europe|Netherlands|2009
MG847641.1|North America|USA|
MG849867.1|North America|USA|
MG848753.1|North America|USA|
OP751638.1|Europe|United Kingdom|2016
OP751639.1|Europe|United Kingdom|2016
OP728449.1|Europe|United Kingdom|2018
OP751556.1|Europe|United Kingdom|2016
OP751586.1|Europe|United Kingdom|2016
JN565302.1|Europe|Croatia|2001
JN565303.1|Europe|Croatia|2001
KU053871.1|||
MG847755.1|North America|USA|
FJ006723.1|Asia|China: Xinjiang province|2006-12-16
JQ004093.1|Asia|Thailand|2010
MG847939.1|North America|USA|
MG847979.1|North America|USA|
MG848114.1|North America|USA|
MG850250.1|North America|USA|
PV051124.1|Europe|France|2020
MG848186.1|North America|USA|
MG850097.1|North America|USA|
MG848948.1|North America|USA|
MG849412.1|North America|USA|
MG847662.1|North America|USA|
MG849124.1|North America|USA|
MG849866.1|North America|USA|
MG850209.1|North America|USA|
MG850224.1|North America|USA|
MG850312.1|North America|USA|
MG849550.1|North America|USA|
OP751582.1|Europe|United Kingdom|2016
MG848459.1|North America|USA|
KU684316.1|Asia|India|2009
OP751554.1|Europe|United Kingdom|2016
OP751601.1|Europe|United Kingdom|2016
OP751676.1|Europe|United Kingdom|2016
OP751616.1|Europe|United Kingdom|2016
OP751653.1|Europe|United Kingdom|2016
OP751624.1|Europe|United Kingdom|2016
OP728421.1|Europe|United Kingdom|2018
OP751678.1|Europe|United Kingdom|2016
OP751620.1|Europe|United Kingdom|2016
OP751651.1|Europe|United Kingdom|2016
OP728404.1|Europe|United Kingdom|2018
OP728457.1|Europe|United Kingdom|2018
OP728354.1|Europe|United Kingdom|2018
OP728364.1|Europe|United Kingdom|2018
MG848685.1|North America|USA|
MG850126.1|North America|USA|
MG850010.1|North America|USA|
MG850419.1|North America|USA|
MG850630.1|North America|USA|
MG850529.1|North America|USA|
MG847813.1|North America|USA|
KX947282.1|Asia|Nepal|2014
MG848998.1|North America|USA|
MG848364.1|North America|USA|
MG849492.1|North America|USA|
KX947278.1|Asia|Nepal|2014
MG848258.1|North America|USA|
MG847778.1|North America|USA|
MG849745.1|North America|USA|
MG848416.1|North America|USA|
MG848995.1|North America|USA|
MG848397.1|North America|USA|
MG849283.1|North America|USA|
MG849917.1|North America|USA|
MG850612.1|North America|USA|
MG848668.1|North America|USA|
MG849799.1|North America|USA|
MG850242.1|North America|USA|
MG847732.1|North America|USA|
MG847973.1|North America|USA|
MG848315.1|North America|USA|
MG848473.1|North America|USA|
MG850277.1|North America|USA|
MG848112.1|North America|USA|
MG849097.1|North America|USA|
MG849476.1|North America|USA|
KX947276.1|Asia|Nepal|2014
MG847735.1|North America|USA|
MG847775.1|North America|USA|
MG847856.1|North America|USA|
MG847885.1|North America|USA|
MG848238.1|North America|USA|
MG848704.1|North America|USA|
MG849408.1|North America|USA|
MG849509.1|North America|USA|
MG849992.1|North America|USA|
OP728380.1|Europe|United Kingdom|2018
MG848782.1|North America|USA|
MG848912.1|North America|USA|
MG849713.1|North America|USA|
MG848443.1|North America|USA|
MG848491.1|North America|USA|
MG847629.1|North America|USA|
MG848033.1|North America|USA|
MG849646.1|North America|USA|
MG849968.1|North America|USA|
MG848381.1|North America|USA|
PV809653.1|Europe|Latvia|2023

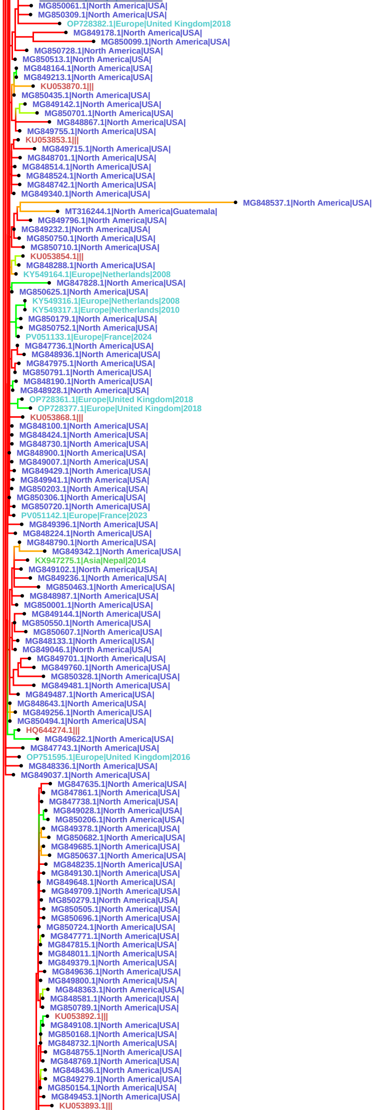
MG850061.1|North America|USA|
MG850309.1|North America|USA|
OP728382.1|Europe|United Kingdom|2018
MG849178.1|North America|USA|
MG850099.1|North America|USA|
MG850728.1|North America|USA|
MG850513.1|North America|USA|
MG848164.1|North America|USA|
MG849213.1|North America|USA|
KU053870.1|||
MG850435.1|North America|USA|
MG849142.1|North America|USA|
MG850701.1|North America|USA|
MG848867.1|North America|USA|
MG849755.1|North America|USA|
KU053853.1|||
MG849715.1|North America|USA|
MG848701.1|North America|USA|
MG848514.1|North America|USA|
MG848524.1|North America|USA|
MG848742.1|North America|USA|
MG849340.1|North America|USA|
MG848537.1|North America|USA|
MT316244.1|North America|Guatemala|
MG849796.1|North America|USA|
MG849232.1|North America|USA|
MG850750.1|North America|USA|
MG850710.1|North America|USA|
KU053854.1|||
MG848288.1|North America|USA|
KY549164.1|Europe|Netherlands|2008
MG847828.1|North America|USA|
MG850625.1|North America|USA|
KY549316.1|Europe|Netherlands|2008
KY549317.1|Europe|Netherlands|2010
MG850179.1|North America|USA|
MG850752.1|North America|USA|
PV051133.1|Europe|France|2024
MG847736.1|North America|USA|
MG848936.1|North America|USA|
MG847975.1|North America|USA|
MG850791.1|North America|USA|
MG848190.1|North America|USA|
MG848928.1|North America|USA|
OP728361.1|Europe|United Kingdom|2018
OP728377.1|Europe|United Kingdom|2018
KU053868.1|||
MG848100.1|North America|USA|
MG848424.1|North America|USA|
MG848730.1|North America|USA|
MG848900.1|North America|USA|
MG849007.1|North America|USA|
MG849429.1|North America|USA|
MG849941.1|North America|USA|
MG850203.1|North America|USA|
MG850306.1|North America|USA|
MG850720.1|North America|USA|
PV051142.1|Europe|France|2023
MG849396.1|North America|USA|
MG848224.1|North America|USA|
MG848790.1|North America|USA|
MG849342.1|North America|USA|
KX947275.1|Asia|Nepal|2014
MG849102.1|North America|USA|
MG849236.1|North America|USA|
MG850463.1|North America|USA|
MG848987.1|North America|USA|
MG850001.1|North America|USA|
MG849144.1|North America|USA|
MG850550.1|North America|USA|
MG850607.1|North America|USA|
MG848133.1|North America|USA|
MG849046.1|North America|USA|
MG849701.1|North America|USA|
MG849760.1|North America|USA|
MG850328.1|North America|USA|
MG849481.1|North America|USA|
MG849487.1|North America|USA|
MG848643.1|North America|USA|
MG849256.1|North America|USA|
MG850494.1|North America|USA|
HQ644274.1|||
MG849622.1|North America|USA|
MG847743.1|North America|USA|
OP751595.1|Europe|United Kingdom|2016
MG848336.1|North America|USA|
MG849037.1|North America|USA|
MG847635.1|North America|USA|
MG847861.1|North America|USA|
MG847738.1|North America|USA|
MG849028.1|North America|USA|
MG850206.1|North America|USA|
MG849378.1|North America|USA|
MG850682.1|North America|USA|
MG849685.1|North America|USA|
MG850637.1|North America|USA|
MG848235.1|North America|USA|
MG849130.1|North America|USA|
MG849648.1|North America|USA|
MG849709.1|North America|USA|
MG850279.1|North America|USA|
MG850505.1|North America|USA|
MG850696.1|North America|USA|
MG850724.1|North America|USA|
MG847771.1|North America|USA|
MG847815.1|North America|USA|
MG848011.1|North America|USA|
MG849379.1|North America|USA|
MG849636.1|North America|USA|
MG849800.1|North America|USA|
MG848363.1|North America|USA|
MG848581.1|North America|USA|
MG850789.1|North America|USA|
KU053892.1|||
MG849108.1|North America|USA|
MG850168.1|North America|USA|
MG848732.1|North America|USA|
MG848755.1|North America|USA|
MG848769.1|North America|USA|
MG848436.1|North America|USA|
MG849279.1|North America|USA|
MG850154.1|North America|USA|
MG849453.1|North America|USA|
KU053893.1|||

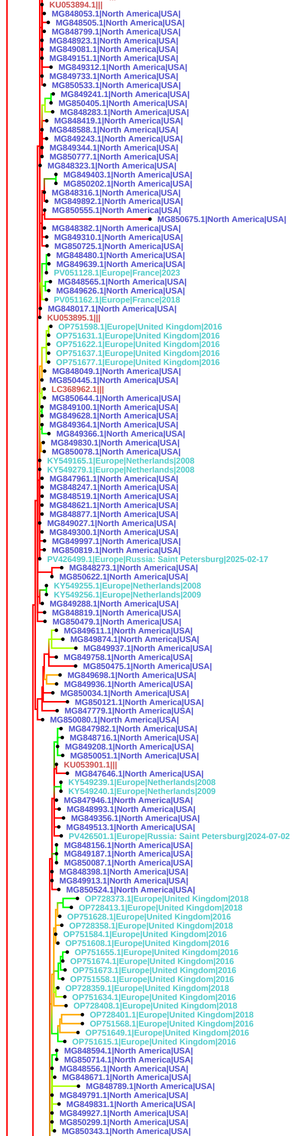
KU053894.1|||
MG848053.1|North America|USA|
MG848505.1|North America|USA|
MG848799.1|North America|USA|
MG848923.1|North America|USA|
MG849081.1|North America|USA|
MG849151.1|North America|USA|
MG849312.1|North America|USA|
MG849733.1|North America|USA|
MG850533.1|North America|USA|
MG849241.1|North America|USA|
MG850405.1|North America|USA|
MG848283.1|North America|USA|
MG848419.1|North America|USA|
MG848588.1|North America|USA|
MG849243.1|North America|USA|
MG849344.1|North America|USA|
MG850777.1|North America|USA|
MG848323.1|North America|USA|
MG849403.1|North America|USA|
MG850202.1|North America|USA|
MG848316.1|North America|USA|
MG849892.1|North America|USA|
MG850555.1|North America|USA|
MG850675.1|North America|USA|
MG848382.1|North America|USA|
MG849310.1|North America|USA|
MG850725.1|North America|USA|
MG848480.1|North America|USA|
MG849639.1|North America|USA|
PV051128.1|Europe|France|2023
MG848565.1|North America|USA|
MG849626.1|North America|USA|
PV051162.1|Europe|France|2018
MG848017.1|North America|USA|
KU053895.1|||
OP751598.1|Europe|United Kingdom|2016
OP751631.1|Europe|United Kingdom|2016
OP751622.1|Europe|United Kingdom|2016
OP751637.1|Europe|United Kingdom|2016
OP751677.1|Europe|United Kingdom|2016
MG848049.1|North America|USA|
MG850445.1|North America|USA|
LC368962.1|||
MG850644.1|North America|USA|
MG849100.1|North America|USA|
MG849628.1|North America|USA|
MG849364.1|North America|USA|
MG849366.1|North America|USA|
MG849830.1|North America|USA|
MG850078.1|North America|USA|
KY549165.1|Europe|Netherlands|2008
KY549279.1|Europe|Netherlands|2008
MG847961.1|North America|USA|
MG848247.1|North America|USA|
MG848519.1|North America|USA|
MG848621.1|North America|USA|
MG848877.1|North America|USA|
MG849027.1|North America|USA|
MG849300.1|North America|USA|
MG849997.1|North America|USA|
MG850819.1|North America|USA|
PV426499.1|Europe|Russia: Saint Petersburg|2025-02-17
MG848273.1|North America|USA|
MG850622.1|North America|USA|
KY549255.1|Europe|Netherlands|2008
KY549256.1|Europe|Netherlands|2009
MG849288.1|North America|USA|
MG848819.1|North America|USA|
MG850479.1|North America|USA|
MG849611.1|North America|USA|
MG849874.1|North America|USA|
MG849937.1|North America|USA|
MG849758.1|North America|USA|
MG850475.1|North America|USA|
MG849698.1|North America|USA|
MG849936.1|North America|USA|
MG850034.1|North America|USA|
MG850121.1|North America|USA|
MG847779.1|North America|USA|
MG850080.1|North America|USA|
MG847982.1|North America|USA|
MG848716.1|North America|USA|
MG849208.1|North America|USA|
MG850051.1|North America|USA|
KU053901.1|||
MG847646.1|North America|USA|
KY549239.1|Europe|Netherlands|2008
KY549240.1|Europe|Netherlands|2009
MG847946.1|North America|USA|
MG848993.1|North America|USA|
MG849356.1|North America|USA|
MG849513.1|North America|USA|
PV426501.1|Europe|Russia: Saint Petersburg|2024-07-02
MG848156.1|North America|USA|
MG849187.1|North America|USA|
MG850087.1|North America|USA|
MG848398.1|North America|USA|
MG849913.1|North America|USA|
MG850524.1|North America|USA|
OP728373.1|Europe|United Kingdom|2018
OP728413.1|Europe|United Kingdom|2018
OP751628.1|Europe|United Kingdom|2016
OP728358.1|Europe|United Kingdom|2018
OP751584.1|Europe|United Kingdom|2016
OP751608.1|Europe|United Kingdom|2016
OP751655.1|Europe|United Kingdom|2016
OP751674.1|Europe|United Kingdom|2016
OP751673.1|Europe|United Kingdom|2016
OP751558.1|Europe|United Kingdom|2016
OP728359.1|Europe|United Kingdom|2018
OP751634.1|Europe|United Kingdom|2016
OP728408.1|Europe|United Kingdom|2018
OP728401.1|Europe|United Kingdom|2018
OP751568.1|Europe|United Kingdom|2016
OP751649.1|Europe|United Kingdom|2016
OP751615.1|Europe|United Kingdom|2016
MG848594.1|North America|USA|
MG850714.1|North America|USA|
MG848556.1|North America|USA|
MG848671.1|North America|USA|
MG848789.1|North America|USA|
MG849791.1|North America|USA|
MG849831.1|North America|USA|
MG849927.1|North America|USA|
MG850299.1|North America|USA|
MG850343.1|North America|USA|

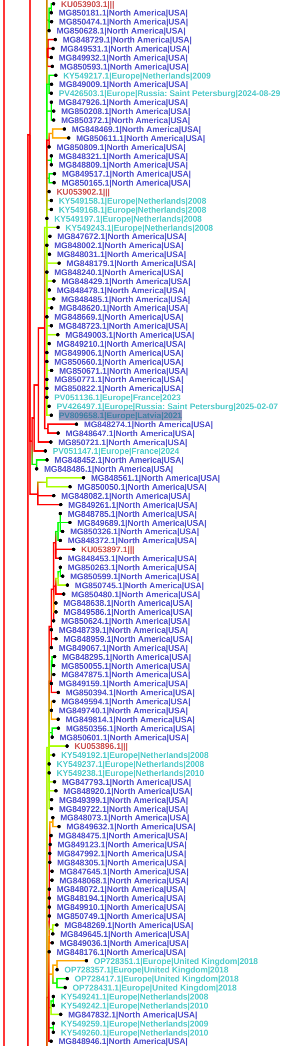
KU053903.1|||
MG850181.1|North America|USA|
MG850474.1|North America|USA|
MG850628.1|North America|USA|
MG848729.1|North America|USA|
MG849531.1|North America|USA|
MG849932.1|North America|USA|
MG850593.1|North America|USA|
KY549217.1|Europe|Netherlands|2009
MG849009.1|North America|USA|
PV426503.1|Europe|Russia: Saint Petersburg|2024-08-29
MG847926.1|North America|USA|
MG850208.1|North America|USA|
MG850372.1|North America|USA|
MG848469.1|North America|USA|
MG850611.1|North America|USA|
MG850809.1|North America|USA|
MG848321.1|North America|USA|
MG848809.1|North America|USA|
MG849517.1|North America|USA|
MG850165.1|North America|USA|
KU053902.1|||
KY549158.1|Europe|Netherlands|2008
KY549168.1|Europe|Netherlands|2008
KY549197.1|Europe|Netherlands|2008
KY549243.1|Europe|Netherlands|2008
MG847672.1|North America|USA|
MG848002.1|North America|USA|
MG848031.1|North America|USA|
MG848179.1|North America|USA|
MG848240.1|North America|USA|
MG848429.1|North America|USA|
MG848478.1|North America|USA|
MG848485.1|North America|USA|
MG848620.1|North America|USA|
MG848669.1|North America|USA|
MG848723.1|North America|USA|
MG849003.1|North America|USA|
MG849210.1|North America|USA|
MG849906.1|North America|USA|
MG850660.1|North America|USA|
MG850671.1|North America|USA|
MG850771.1|North America|USA|
MG850822.1|North America|USA|
PV051136.1|Europe|France|2023
PV426497.1|Europe|Russia: Saint Petersburg|2025-02-07
PV809658.1|Europe|Latvia|2021
MG848274.1|North America|USA|
MG848647.1|North America|USA|
MG850721.1|North America|USA|
PV051147.1|Europe|France|2024
MG848452.1|North America|USA|
MG848486.1|North America|USA|
MG848561.1|North America|USA|
MG850050.1|North America|USA|
MG848082.1|North America|USA|
MG849261.1|North America|USA|
MG848785.1|North America|USA|
MG849689.1|North America|USA|
MG850326.1|North America|USA|
MG848372.1|North America|USA|
KU053897.1|||
MG848453.1|North America|USA|
MG850263.1|North America|USA|
MG850599.1|North America|USA|
MG850745.1|North America|USA|
MG850480.1|North America|USA|
MG848638.1|North America|USA|
MG849586.1|North America|USA|
MG850624.1|North America|USA|
MG848739.1|North America|USA|
MG848959.1|North America|USA|
MG849067.1|North America|USA|
MG848295.1|North America|USA|
MG850055.1|North America|USA|
MG847875.1|North America|USA|
MG849159.1|North America|USA|
MG850394.1|North America|USA|
MG849594.1|North America|USA|
MG849740.1|North America|USA|
MG849814.1|North America|USA|
MG850356.1|North America|USA|
MG850601.1|North America|USA|
KU053896.1|||
KY549192.1|Europe|Netherlands|2008
KY549237.1|Europe|Netherlands|2008
KY549238.1|Europe|Netherlands|2010
MG847793.1|North America|USA|
MG848920.1|North America|USA|
MG849399.1|North America|USA|
MG849722.1|North America|USA|
MG848073.1|North America|USA|
MG849632.1|North America|USA|
MG848475.1|North America|USA|
MG849123.1|North America|USA|
MG847992.1|North America|USA|
MG848305.1|North America|USA|
MG847645.1|North America|USA|
MG848068.1|North America|USA|
MG848072.1|North America|USA|
MG848194.1|North America|USA|
MG849910.1|North America|USA|
MG850749.1|North America|USA|
MG848269.1|North America|USA|
MG849645.1|North America|USA|
MG849036.1|North America|USA|
MG848176.1|North America|USA|
OP728351.1|Europe|United Kingdom|2018
OP728357.1|Europe|United Kingdom|2018
OP728417.1|Europe|United Kingdom|2018
OP728431.1|Europe|United Kingdom|2018
KY549241.1|Europe|Netherlands|2008
KY549242.1|Europe|Netherlands|2010
MG847832.1|North America|USA|
KY549259.1|Europe|Netherlands|2009
KY549260.1|Europe|Netherlands|2010
MG848946.1|North America|USA|

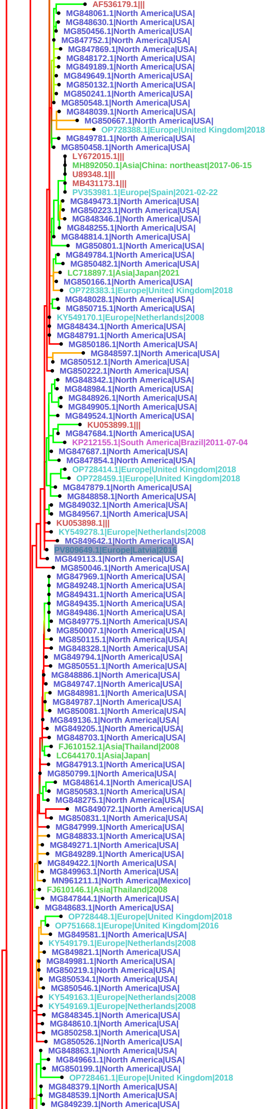
AF536179.1|||
MG848061.1|North America|USA|
MG848630.1|North America|USA|
MG850456.1|North America|USA|
MG847752.1|North America|USA|
MG847869.1|North America|USA|
MG848172.1|North America|USA|
MG849189.1|North America|USA|
MG849649.1|North America|USA|
MG850132.1|North America|USA|
MG850241.1|North America|USA|
MG850548.1|North America|USA|
MG848039.1|North America|USA|
MG850667.1|North America|USA|
OP728388.1|Europe|United Kingdom|2018
MG849781.1|North America|USA|
MG850458.1|North America|USA|
LY672015.1|||
MH892050.1|Asia|China: northeast|2017-06-15
U89348.1|||
MB431173.1|||
PV353981.1|Europe|Spain|2021-02-22
MG849473.1|North America|USA|
MG850223.1|North America|USA|
MG848346.1|North America|USA|
MG848255.1|North America|USA|
MG848814.1|North America|USA|
MG850801.1|North America|USA|
MG849784.1|North America|USA|
MG850482.1|North America|USA|
LC718897.1|Asia|Japan|2021
MG850166.1|North America|USA|
OP728383.1|Europe|United Kingdom|2018
MG848028.1|North America|USA|
MG850715.1|North America|USA|
KY549170.1|Europe|Netherlands|2008
MG848434.1|North America|USA|
MG848791.1|North America|USA|
MG850186.1|North America|USA|
MG848597.1|North America|USA|
MG850512.1|North America|USA|
MG850222.1|North America|USA|
MG848342.1|North America|USA|
MG848984.1|North America|USA|
MG848926.1|North America|USA|
MG849905.1|North America|USA|
MG849524.1|North America|USA|
KU053899.1|||
MG847684.1|North America|USA|
KP212155.1|South America|Brazil|2011-07-04
MG847687.1|North America|USA|
MG847854.1|North America|USA|
OP728414.1|Europe|United Kingdom|2018
OP728459.1|Europe|United Kingdom|2018
MG847879.1|North America|USA|
MG848858.1|North America|USA|
MG849032.1|North America|USA|
MG849567.1|North America|USA|
KU053898.1|||
KY549278.1|Europe|Netherlands|2008
MG849642.1|North America|USA|
PV809649.1|Europe|Latvia|2016
MG849113.1|North America|USA|
MG850046.1|North America|USA|
MG847969.1|North America|USA|
MG849248.1|North America|USA|
MG849431.1|North America|USA|
MG849435.1|North America|USA|
MG849486.1|North America|USA|
MG849775.1|North America|USA|
MG850007.1|North America|USA|
MG850115.1|North America|USA|
MG848328.1|North America|USA|
MG849794.1|North America|USA|
MG850551.1|North America|USA|
MG848886.1|North America|USA|
MG849747.1|North America|USA|
MG848981.1|North America|USA|
MG849787.1|North America|USA|
MG850081.1|North America|USA|
MG849136.1|North America|USA|
MG849205.1|North America|USA|
MG848703.1|North America|USA|
FJ610152.1|Asia|Thailand|2008
LC644170.1|Asia|Japan|
MG847913.1|North America|USA|
MG850799.1|North America|USA|
MG848614.1|North America|USA|
MG850583.1|North America|USA|
MG848275.1|North America|USA|
MG849072.1|North America|USA|
MG850831.1|North America|USA|
MG847999.1|North America|USA|
MG848833.1|North America|USA|
MG849271.1|North America|USA|
MG849289.1|North America|USA|
MG849422.1|North America|USA|
MG849963.1|North America|USA|
MN961211.1|North America|Mexico|
FJ610146.1|Asia|Thailand|2008
MG847844.1|North America|USA|
MG848683.1|North America|USA|
OP728448.1|Europe|United Kingdom|2018
OP751668.1|Europe|United Kingdom|2016
MG849581.1|North America|USA|
KY549179.1|Europe|Netherlands|2008
MG849821.1|North America|USA|
MG849981.1|North America|USA|
MG850219.1|North America|USA|
MG850534.1|North America|USA|
MG850546.1|North America|USA|
KY549163.1|Europe|Netherlands|2008
KY549169.1|Europe|Netherlands|2008
MG848345.1|North America|USA|
MG848610.1|North America|USA|
MG850258.1|North America|USA|
MG850526.1|North America|USA|
MG848863.1|North America|USA|
MG849661.1|North America|USA|
MG850199.1|North America|USA|
OP728461.1|Europe|United Kingdom|2018
MG848379.1|North America|USA|
MG848539.1|North America|USA|
MG849239.1|North America|USA|

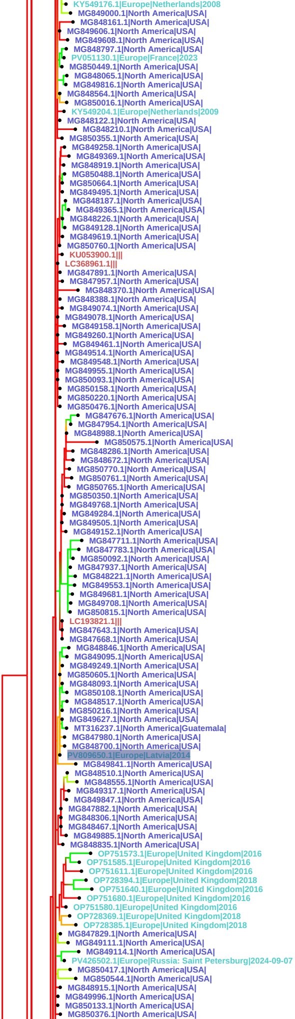
KY549176.1|Europe|Netherlands|2008
MG849000.1|North America|USA|
MG848161.1|North America|USA|
MG849606.1|North America|USA|
MG849608.1|North America|USA|
MG848797.1|North America|USA|
PV051130.1|Europe|France|2023
MG850449.1|North America|USA|
MG848065.1|North America|USA|
MG849816.1|North America|USA|
MG848564.1|North America|USA|
MG850016.1|North America|USA|
KY549204.1|Europe|Netherlands|2009
MG848122.1|North America|USA|
MG848210.1|North America|USA|
MG850355.1|North America|USA|
MG849258.1|North America|USA|
MG849369.1|North America|USA|
MG848919.1|North America|USA|
MG850488.1|North America|USA|
MG850664.1|North America|USA|
MG849495.1|North America|USA|
MG848187.1|North America|USA|
MG849365.1|North America|USA|
MG848226.1|North America|USA|
MG849128.1|North America|USA|
MG849619.1|North America|USA|
MG850760.1|North America|USA|
KU053900.1|||
LC368961.1|||
MG847891.1|North America|USA|
MG847957.1|North America|USA|
MG848370.1|North America|USA|
MG848388.1|North America|USA|
MG849074.1|North America|USA|
MG849078.1|North America|USA|
MG849158.1|North America|USA|
MG849260.1|North America|USA|
MG849461.1|North America|USA|
MG849514.1|North America|USA|
MG849548.1|North America|USA|
MG849955.1|North America|USA|
MG850093.1|North America|USA|
MG850158.1|North America|USA|
MG850220.1|North America|USA|
MG850476.1|North America|USA|
MG847676.1|North America|USA|
MG847954.1|North America|USA|
MG848988.1|North America|USA|
MG850575.1|North America|USA|
MG848286.1|North America|USA|
MG848672.1|North America|USA|
MG850770.1|North America|USA|
MG850761.1|North America|USA|
MG850765.1|North America|USA|
MG850350.1|North America|USA|
MG849768.1|North America|USA|
MG849284.1|North America|USA|
MG849505.1|North America|USA|
MG849152.1|North America|USA|
MG847711.1|North America|USA|
MG847783.1|North America|USA|
MG850092.1|North America|USA|
MG847937.1|North America|USA|
MG848221.1|North America|USA|
MG849553.1|North America|USA|
MG849681.1|North America|USA|
MG849708.1|North America|USA|
MG850815.1|North America|USA|
LC193821.1|||
MG847643.1|North America|USA|
MG847668.1|North America|USA|
MG848846.1|North America|USA|
MG849095.1|North America|USA|
MG849249.1|North America|USA|
MG850605.1|North America|USA|
MG848093.1|North America|USA|
MG850108.1|North America|USA|
MG848517.1|North America|USA|
MG850216.1|North America|USA|
MG849627.1|North America|USA|
MT316237.1|North America|Guatemala|
MG847980.1|North America|USA|
MG848700.1|North America|USA|
PV809650.1|Europe|Latvia|2014
MG849841.1|North America|USA|
MG848510.1|North America|USA|
MG848555.1|North America|USA|
MG849317.1|North America|USA|
MG849847.1|North America|USA|
MG847882.1|North America|USA|
MG848306.1|North America|USA|
MG848467.1|North America|USA|
MG849885.1|North America|USA|
MG848835.1|North America|USA|
OP751573.1|Europe|United Kingdom|2016
OP751585.1|Europe|United Kingdom|2016
OP751611.1|Europe|United Kingdom|2016
OP728394.1|Europe|United Kingdom|2018
OP751640.1|Europe|United Kingdom|2016
OP751680.1|Europe|United Kingdom|2016
OP751580.1|Europe|United Kingdom|2016
OP728369.1|Europe|United Kingdom|2018
OP728385.1|Europe|United Kingdom|2018
MG847829.1|North America|USA|
MG849111.1|North America|USA|
MG849114.1|North America|USA|
PV426502.1|Europe|Russia: Saint Petersburg|2024-09-07
MG850417.1|North America|USA|
MG850544.1|North America|USA|
MG848915.1|North America|USA|
MG849996.1|North America|USA|
MG850133.1|North America|USA|
MG850376.1|North America|USA|

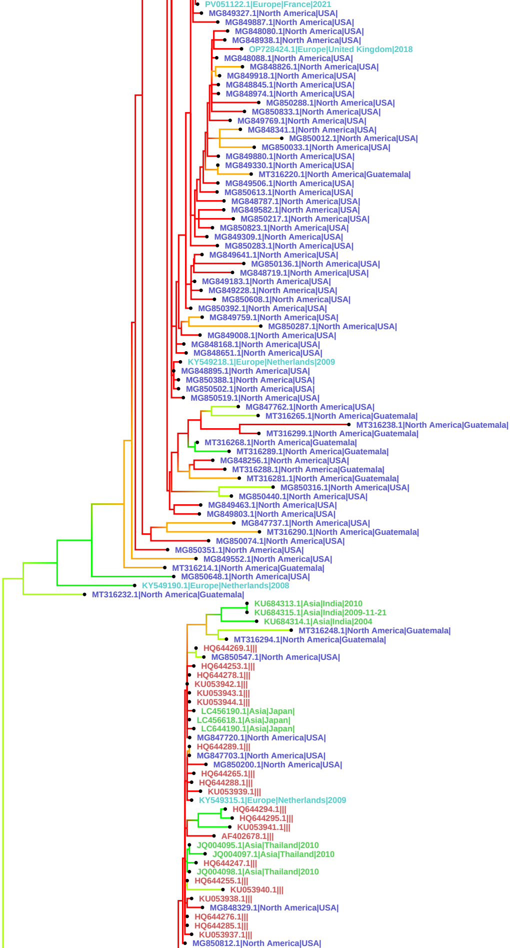
PV051122.1|Europe|France|2021
MG849327.1|North America|USA|
MG849887.1|North America|USA|
MG848080.1|North America|USA|
MG848938.1|North America|USA|
OP728424.1|Europe|United Kingdom|2018
MG848088.1|North America|USA|
MG848826.1|North America|USA|
MG849918.1|North America|USA|
MG848845.1|North America|USA|
MG848974.1|North America|USA|
MG850288.1|North America|USA|
MG850833.1|North America|USA|
MG849769.1|North America|USA|
MG848341.1|North America|USA|
MG850012.1|North America|USA|
MG850033.1|North America|USA|
MG849880.1|North America|USA|
MG849330.1|North America|USA|
MT316220.1|North America|Guatemala|
MG849506.1|North America|USA|
MG850613.1|North America|USA|
MG848787.1|North America|USA|
MG849582.1|North America|USA|
MG850217.1|North America|USA|
MG850823.1|North America|USA|
MG849309.1|North America|USA|
MG850283.1|North America|USA|
MG849641.1|North America|USA|
MG850136.1|North America|USA|
MG848719.1|North America|USA|
MG849183.1|North America|USA|
MG849228.1|North America|USA|
MG850608.1|North America|USA|
MG850392.1|North America|USA|
MG849759.1|North America|USA|
MG850287.1|North America|USA|
MG849008.1|North America|USA|
MG848168.1|North America|USA|
MG848651.1|North America|USA|
KY549218.1|Europe|Netherlands|2009
MG848895.1|North America|USA|
MG850388.1|North America|USA|
MG850502.1|North America|USA|
MG850519.1|North America|USA|
MG847762.1|North America|USA|
MT316265.1|North America|Guatemala|
MT316238.1|North America|Guatemala|
MT316299.1|North America|Guatemala|
MT316268.1|North America|Guatemala|
MT316289.1|North America|Guatemala|
MG848256.1|North America|USA|
MT316288.1|North America|Guatemala|
MT316281.1|North America|Guatemala|
MG850316.1|North America|USA|
MG850440.1|North America|USA|
MG849463.1|North America|USA|
MG849803.1|North America|USA|
MG847737.1|North America|USA|
MT316290.1|North America|Guatemala|
MG850074.1|North America|USA|
MG850351.1|North America|USA|
MG849552.1|North America|USA|
MT316214.1|North America|Guatemala|
MG850648.1|North America|USA|
KY549190.1|Europe|Netherlands|2008
MT316232.1|North America|Guatemala|
KU684313.1|Asia|India|2010
KU684315.1|Asia|India|2009-11-21
KU684314.1|Asia|India|2004
MT316248.1|North America|Guatemala|
MT316294.1|North America|Guatemala|
HQ644269.1|||
MG850547.1|North America|USA|
HQ644253.1|||
HQ644278.1|||
KU053942.1|||
KU053943.1|||
KU053944.1|||
LC456190.1|Asia|Japan|
LC456618.1|Asia|Japan|
LC644190.1|Asia|Japan|
MG847720.1|North America|USA|
HQ644289.1|||
MG847703.1|North America|USA|
MG850200.1|North America|USA|
HQ644265.1|||
HQ644288.1|||
KU053939.1|||
KY549315.1|Europe|Netherlands|2009
HQ644294.1|||
HQ644295.1|||
KU053941.1|||
AF402678.1|||
JQ004095.1|Asia|Thailand|2010
JQ004097.1|Asia|Thailand|2010
HQ644247.1|||
JQ004098.1|Asia|Thailand|2010
HQ644255.1|||
KU053940.1|||
KU053938.1|||
MG848329.1|North America|USA|
HQ644276.1|||
HQ644285.1|||
KU053937.1|||
MG850812.1|North America|USA|

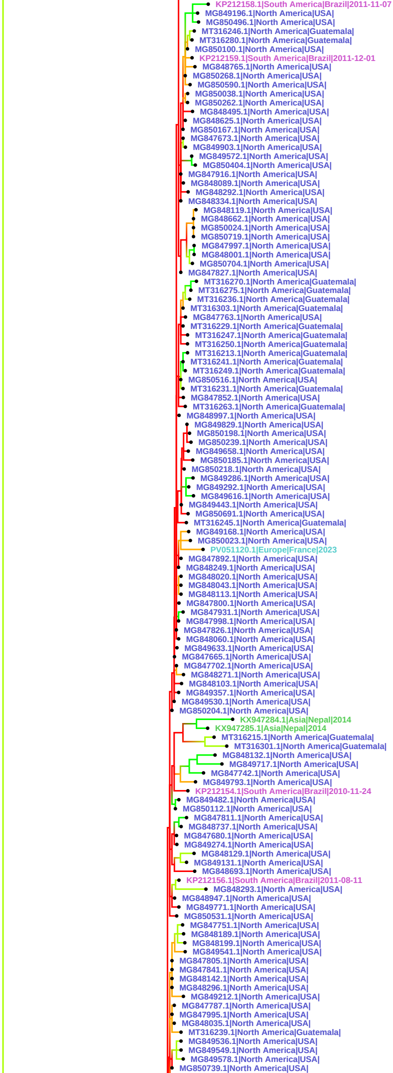
KP212158.1|South America|Brazil|2011-11-07
MG849196.1|North America|USA|
MG850496.1|North America|USA|
MT316246.1|North America|Guatemala|
MT316280.1|North America|Guatemala|
MG850100.1|North America|USA|
KP212159.1|South America|Brazil|2011-12-01
MG848765.1|North America|USA|
MG850268.1|North America|USA|
MG850590.1|North America|USA|
MG850038.1|North America|USA|
MG850262.1|North America|USA|
MG848495.1|North America|USA|
MG848625.1|North America|USA|
MG850167.1|North America|USA|
MG847673.1|North America|USA|
MG849903.1|North America|USA|
MG849572.1|North America|USA|
MG850404.1|North America|USA|
MG847916.1|North America|USA|
MG848089.1|North America|USA|
MG848292.1|North America|USA|
MG848334.1|North America|USA|
MG848119.1|North America|USA|
MG848662.1|North America|USA|
MG850024.1|North America|USA|
MG850719.1|North America|USA|
MG847997.1|North America|USA|
MG848001.1|North America|USA|
MG850704.1|North America|USA|
MG847827.1|North America|USA|
MT316270.1|North America|Guatemala|
MT316275.1|North America|Guatemala|
MT316236.1|North America|Guatemala|
MT316303.1|North America|Guatemala|
MG847763.1|North America|USA|
MT316229.1|North America|Guatemala|
MT316247.1|North America|Guatemala|
MT316250.1|North America|Guatemala|
MT316213.1|North America|Guatemala|
MT316241.1|North America|Guatemala|
MT316249.1|North America|Guatemala|
MG850516.1|North America|USA|
MT316231.1|North America|Guatemala|
MG847852.1|North America|USA|
MT316263.1|North America|Guatemala|
MG848997.1|North America|USA|
MG849829.1|North America|USA|
MG850198.1|North America|USA|
MG850239.1|North America|USA|
MG849658.1|North America|USA|
MG850185.1|North America|USA|
MG850218.1|North America|USA|
MG849286.1|North America|USA|
MG849292.1|North America|USA|
MG849616.1|North America|USA|
MG849443.1|North America|USA|
MG850691.1|North America|USA|
MT316245.1|North America|Guatemala|
MG849168.1|North America|USA|
MG850023.1|North America|USA|
PV051120.1|Europe|France|2023
MG847892.1|North America|USA|
MG848249.1|North America|USA|
MG848020.1|North America|USA|
MG848043.1|North America|USA|
MG848113.1|North America|USA|
MG847800.1|North America|USA|
MG847931.1|North America|USA|
MG847998.1|North America|USA|
MG847826.1|North America|USA|
MG848060.1|North America|USA|
MG849633.1|North America|USA|
MG847665.1|North America|USA|
MG847702.1|North America|USA|
MG848271.1|North America|USA|
MG848103.1|North America|USA|
MG849357.1|North America|USA|
MG849530.1|North America|USA|
MG850204.1|North America|USA|
KX947284.1|Asia|Nepal|2014
KX947285.1|Asia|Nepal|2014
MT316215.1|North America|Guatemala|
MT316301.1|North America|Guatemala|
MG848132.1|North America|USA|
MG849717.1|North America|USA|
MG847742.1|North America|USA|
MG849793.1|North America|USA|
KP212154.1|South America|Brazil|2010-11-24
MG849482.1|North America|USA|
MG850112.1|North America|USA|
MG847811.1|North America|USA|
MG848737.1|North America|USA|
MG847680.1|North America|USA|
MG849274.1|North America|USA|
MG848129.1|North America|USA|
MG849131.1|North America|USA|
MG848693.1|North America|USA|
KP212156.1|South America|Brazil|2011-08-11
MG848293.1|North America|USA|
MG848947.1|North America|USA|
MG849771.1|North America|USA|
MG850531.1|North America|USA|
MG847751.1|North America|USA|
MG848189.1|North America|USA|
MG848199.1|North America|USA|
MG849541.1|North America|USA|
MG847805.1|North America|USA|
MG847841.1|North America|USA|
MG848142.1|North America|USA|
MG848296.1|North America|USA|
MG849212.1|North America|USA|
MG847787.1|North America|USA|
MG847995.1|North America|USA|
MG848035.1|North America|USA|
MT316239.1|North America|Guatemala|
MG849536.1|North America|USA|
MG849549.1|North America|USA|
MG849578.1|North America|USA|
MG850739.1|North America|USA|

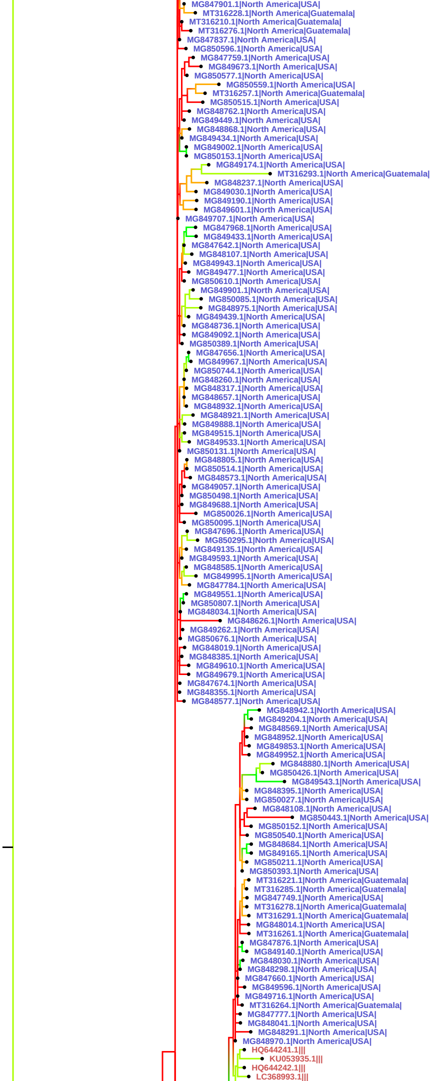

MG847901.1|North America|USA|
MT316228.1|North America|Guatemala|
MT316210.1|North America|Guatemala|
MT316276.1|North America|Guatemala|
MG847837.1|North America|USA|
MG850596.1|North America|USA|
MG847759.1|North America|USA|
MG849673.1|North America|USA|
MG850577.1|North America|USA|
MG850559.1|North America|USA|
MT316257.1|North America|Guatemala|
MG850515.1|North America|USA|
MG848762.1|North America|USA|
MG849449.1|North America|USA|
MG848868.1|North America|USA|
MG849434.1|North America|USA|
MG849002.1|North America|USA|
MG850153.1|North America|USA|
MG849174.1|North America|USA|
MT316293.1|North America|Guatemala|
MG848237.1|North America|USA|
MG849030.1|North America|USA|
MG849190.1|North America|USA|
MG849601.1|North America|USA|
MG849707.1|North America|USA|
MG847968.1|North America|USA|
MG849433.1|North America|USA|
MG847642.1|North America|USA|
MG848107.1|North America|USA|
MG849943.1|North America|USA|
MG849477.1|North America|USA|
MG850610.1|North America|USA|
MG849901.1|North America|USA|
MG850085.1|North America|USA|
MG848975.1|North America|USA|
MG849439.1|North America|USA|
MG848736.1|North America|USA|
MG849092.1|North America|USA|
MG850389.1|North America|USA|
MG847656.1|North America|USA|
MG849967.1|North America|USA|
MG850744.1|North America|USA|
MG848260.1|North America|USA|
MG848317.1|North America|USA|
MG848657.1|North America|USA|
MG848932.1|North America|USA|
MG848921.1|North America|USA|
MG849888.1|North America|USA|
MG849515.1|North America|USA|
MG849533.1|North America|USA|
MG850131.1|North America|USA|
MG848805.1|North America|USA|
MG850514.1|North America|USA|
MG848573.1|North America|USA|
MG849057.1|North America|USA|
MG850498.1|North America|USA|
MG849688.1|North America|USA|
MG850026.1|North America|USA|
MG850095.1|North America|USA|
MG847696.1|North America|USA|
MG850295.1|North America|USA|
MG849135.1|North America|USA|
MG849593.1|North America|USA|
MG848585.1|North America|USA|
MG849995.1|North America|USA|
MG847784.1|North America|USA|
MG849551.1|North America|USA|
MG850807.1|North America|USA|
MG848034.1|North America|USA|
MG848626.1|North America|USA|
MG849262.1|North America|USA|
MG850676.1|North America|USA|
MG848019.1|North America|USA|
MG848385.1|North America|USA|
MG849610.1|North America|USA|
MG849679.1|North America|USA|
MG847674.1|North America|USA|
MG848355.1|North America|USA|
MG848577.1|North America|USA|
MG848942.1|North America|USA|
MG849204.1|North America|USA|
MG848569.1|North America|USA|
MG848952.1|North America|USA|
MG849853.1|North America|USA|
MG849952.1|North America|USA|
MG848880.1|North America|USA|
MG850426.1|North America|USA|
MG849543.1|North America|USA|
MG848395.1|North America|USA|
MG850027.1|North America|USA|
MG848108.1|North America|USA|
MG850443.1|North America|USA|
MG850152.1|North America|USA|
MG850540.1|North America|USA|
MG848684.1|North America|USA|
MG849165.1|North America|USA|
MG850211.1|North America|USA|
MG850393.1|North America|USA|
MT316221.1|North America|Guatemala|
MT316285.1|North America|Guatemala|
MG847749.1|North America|USA|
MT316278.1|North America|Guatemala|
MT316291.1|North America|Guatemala|
MG848014.1|North America|USA|
MT316261.1|North America|Guatemala|
MG847876.1|North America|USA|
MG849140.1|North America|USA|
MG848030.1|North America|USA|
MG848298.1|North America|USA|
MG847660.1|North America|USA|
MG849596.1|North America|USA|
MG849716.1|North America|USA|
MT316264.1|North America|Guatemala|
MG847777.1|North America|USA|
MG848041.1|North America|USA|
MG848291.1|North America|USA|
MG848970.1|North America|USA|
HQ644241.1|||
KU053935.1|||
HQ644242.1|||
LC368993.1|||

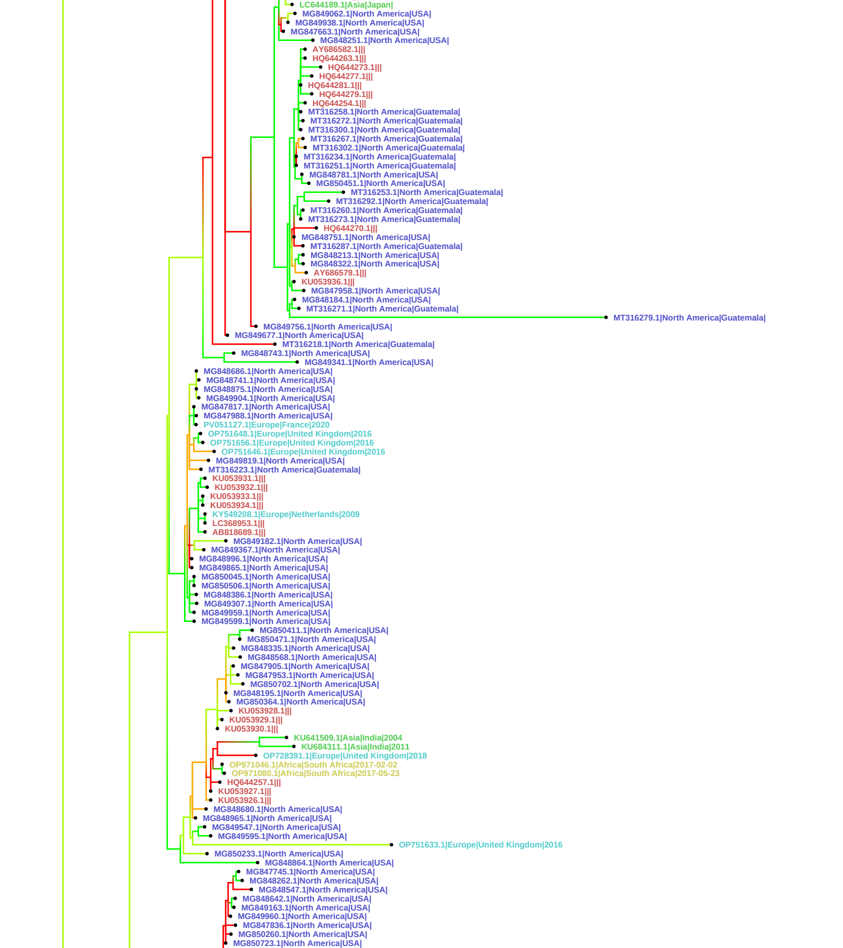
LC644189.1|Asia|Japan|
MG849062.1|North America|USA|
MG849938.1|North America|USA|
MG847663.1|North America|USA|
MG848251.1|North America|USA|
AY686582.1|||
HQ644263.1|||
HQ644273.1|||
HQ644277.1|||
HQ644281.1|||
HQ644279.1|||
HQ644254.1|||
MT316258.1|North America|Guatemala|
MT316272.1|North America|Guatemala|
MT316300.1|North America|Guatemala|
MT316267.1|North America|Guatemala|
MT316302.1|North America|Guatemala|
MT316234.1|North America|Guatemala|
MT316251.1|North America|Guatemala|
MG848781.1|North America|USA|
MG850451.1|North America|USA|
MT316253.1|North America|Guatemala|
MT316292.1|North America|Guatemala|
MT316260.1|North America|Guatemala|
MT316273.1|North America|Guatemala|
HQ644270.1|||
MG848751.1|North America|USA|
MT316287.1|North America|Guatemala|
MG848213.1|North America|USA|
MG848322.1|North America|USA|
AY686579.1|||
KU053936.1|||
MG847958.1|North America|USA|
MG848184.1|North America|USA|
MT316271.1|North America|Guatemala|
MT316279.1|North America|Guatemala|
MG849756.1|North America|USA|
MG849677.1|North America|USA|
MT316218.1|North America|Guatemala|
MG848743.1|North America|USA|
MG849341.1|North America|USA|
MG848686.1|North America|USA|
MG848741.1|North America|USA|
MG848875.1|North America|USA|
MG849904.1|North America|USA|
MG847817.1|North America|USA|
MG847988.1|North America|USA|
PV051127.1|Europe|France|2020
OP751648.1|Europe|United Kingdom|2016
OP751656.1|Europe|United Kingdom|2016
OP751646.1|Europe|United Kingdom|2016
MG849819.1|North America|USA|
MT316223.1|North America|Guatemala|
KU053931.1|||
KU053932.1|||
KU053933.1|||
KU053934.1|||
KY549208.1|Europe|Netherlands|2009
LC368953.1|||
AB818689.1|||
MG849182.1|North America|USA|
MG849367.1|North America|USA|
MG848996.1|North America|USA|
MG849865.1|North America|USA|
MG850045.1|North America|USA|
MG850506.1|North America|USA|
MG848386.1|North America|USA|
MG849307.1|North America|USA|
MG849959.1|North America|USA|
MG849599.1|North America|USA|
MG850411.1|North America|USA|
MG850471.1|North America|USA|
MG848335.1|North America|USA|
MG848568.1|North America|USA|
MG847905.1|North America|USA|
MG847953.1|North America|USA|
MG850702.1|North America|USA|
MG848195.1|North America|USA|
MG850364.1|North America|USA|
KU053928.1|||
KU053929.1|||
KU053930.1|||
KU641509.1|Asia|India|2004
KU684311.1|Asia|India|2011
OP728391.1|Europe|United Kingdom|2018
OP971046.1|Africa|South Africa|2017-02-02
OP971080.1|Africa|South Africa|2017-05-23
HQ644257.1|||
KU053927.1|||
KU053926.1|||
MG848680.1|North America|USA|
MG848965.1|North America|USA|
MG849547.1|North America|USA|
MG849595.1|North America|USA|
OP751633.1|Europe|United Kingdom|2016
MG850233.1|North America|USA|
MG848864.1|North America|USA|
MG847745.1|North America|USA|
MG848262.1|North America|USA|
MG848547.1|North America|USA|
MG848642.1|North America|USA|
MG849163.1|North America|USA|
MG849960.1|North America|USA|
MG847836.1|North America|USA|
MG850260.1|North America|USA|
MG850723.1|North America|USA|

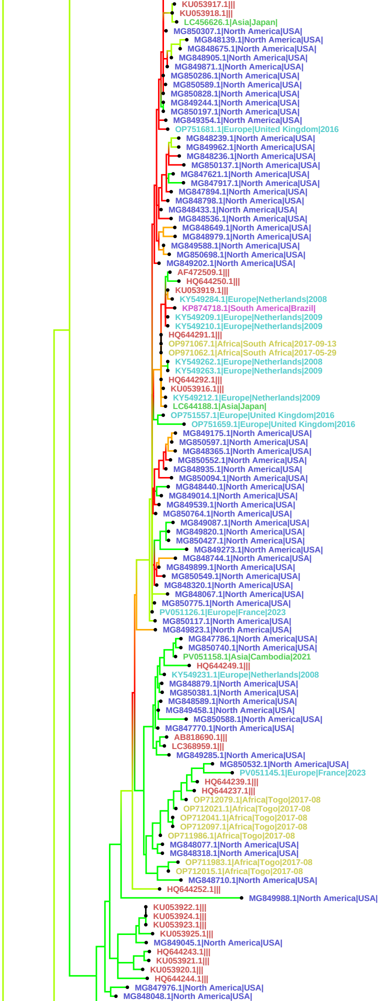
KU053917.1|||
KU053918.1|||
LC456626.1|Asia|Japan|
MG850307.1|North America|USA|
MG848139.1|North America|USA|
MG848675.1|North America|USA|
MG848905.1|North America|USA|
MG849871.1|North America|USA|
MG850286.1|North America|USA|
MG850589.1|North America|USA|
MG850828.1|North America|USA|
MG849244.1|North America|USA|
MG850197.1|North America|USA|
MG849354.1|North America|USA|
OP751681.1|Europe|United Kingdom|2016
MG848239.1|North America|USA|
MG849962.1|North America|USA|
MG848236.1|North America|USA|
MG850137.1|North America|USA|
MG847621.1|North America|USA|
MG847917.1|North America|USA|
MG847894.1|North America|USA|
MG848798.1|North America|USA|
MG848433.1|North America|USA|
MG848536.1|North America|USA|
MG848649.1|North America|USA|
MG848979.1|North America|USA|
MG849588.1|North America|USA|
MG850698.1|North America|USA|
MG849202.1|North America|USA|
AF472509.1|||
HQ644250.1|||
KU053919.1|||
KY549284.1|Europe|Netherlands|2008
KP874718.1|South America|Brazil|
KY549209.1|Europe|Netherlands|2009
KY549210.1|Europe|Netherlands|2009
HQ644291.1|||
OP971067.1|Africa|South Africa|2017-09-13
OP971062.1|Africa|South Africa|2017-05-29
KY549262.1|Europe|Netherlands|2008
KY549263.1|Europe|Netherlands|2009
HQ644292.1|||
KU053916.1|||
KY549212.1|Europe|Netherlands|2009
LC644188.1|Asia|Japan|
OP751557.1|Europe|United Kingdom|2016
OP751659.1|Europe|United Kingdom|2016
MG849175.1|North America|USA|
MG850597.1|North America|USA|
MG848365.1|North America|USA|
MG850552.1|North America|USA|
MG848935.1|North America|USA|
MG850094.1|North America|USA|
MG848440.1|North America|USA|
MG849014.1|North America|USA|
MG849539.1|North America|USA|
MG850764.1|North America|USA|
MG849087.1|North America|USA|
MG849820.1|North America|USA|
MG850427.1|North America|USA|
MG849273.1|North America|USA|
MG848744.1|North America|USA|
MG849899.1|North America|USA|
MG850549.1|North America|USA|
MG848320.1|North America|USA|
MG848067.1|North America|USA|
MG850775.1|North America|USA|
PV051126.1|Europe|France|2023
MG850117.1|North America|USA|
MG849823.1|North America|USA|
MG847786.1|North America|USA|
MG850740.1|North America|USA|
PV051158.1|Asia|Cambodia|2021
HQ644249.1|||
KY549231.1|Europe|Netherlands|2008
MG848879.1|North America|USA|
MG850381.1|North America|USA|
MG848589.1|North America|USA|
MG849458.1|North America|USA|
MG850588.1|North America|USA|
MG847770.1|North America|USA|
AB818690.1|||
LC368959.1|||
MG849285.1|North America|USA|
MG850532.1|North America|USA|
PV051145.1|Europe|France|2023
HQ644239.1|||
HQ644237.1|||
OP712079.1|Africa|Togo|2017-08
OP712021.1|Africa|Togo|2017-08
OP712041.1|Africa|Togo|2017-08
OP712097.1|Africa|Togo|2017-08
OP711986.1|Africa|Togo|2017-08
MG848077.1|North America|USA|
MG848318.1|North America|USA|
OP711983.1|Africa|Togo|2017-08
OP712015.1|Africa|Togo|2017-08
MG848710.1|North America|USA|
HQ644252.1|||
MG849988.1|North America|USA|
KU053922.1|||
KU053924.1|||
KU053923.1|||
KU053925.1|||
MG849045.1|North America|USA|
HQ644243.1|||
KU053921.1|||
KU053920.1|||
HQ644244.1|||
MG847976.1|North America|USA|
MG848048.1|North America|USA|

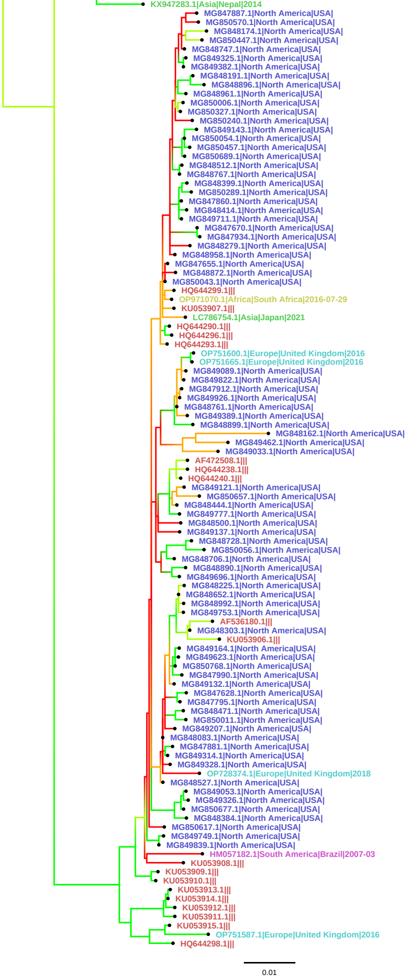
KX947283.1|Asia|Nepal|2014
MG847887.1|North America|USA|
MG850570.1|North America|USA|
MG848174.1|North America|USA|
MG850447.1|North America|USA|
MG848747.1|North America|USA|
MG849325.1|North America|USA|
MG849382.1|North America|USA|
MG848191.1|North America|USA|
MG848896.1|North America|USA|
MG848961.1|North America|USA|
MG850006.1|North America|USA|
MG850327.1|North America|USA|
MG850240.1|North America|USA|
MG849143.1|North America|USA|
MG850054.1|North America|USA|
MG850457.1|North America|USA|
MG850689.1|North America|USA|
MG848512.1|North America|USA|
MG848767.1|North America|USA|
MG848399.1|North America|USA|
MG850289.1|North America|USA|
MG847860.1|North America|USA|
MG848414.1|North America|USA|
MG849711.1|North America|USA|
MG847670.1|North America|USA|
MG847934.1|North America|USA|
MG848279.1|North America|USA|
MG848958.1|North America|USA|
MG847655.1|North America|USA|
MG848872.1|North America|USA|
MG850043.1|North America|USA|
HQ644299.1|||
OP971070.1|Africa|South Africa|2016-07-29
KU053907.1|||
LC786754.1|Asia|Japan|2021
HQ644290.1|||
HQ644296.1|||
HQ644293.1|||
OP751600.1|Europe|United Kingdom|2016
OP751665.1|Europe|United Kingdom|2016
MG849089.1|North America|USA|
MG849822.1|North America|USA|
MG847912.1|North America|USA|
MG849926.1|North America|USA|
MG848761.1|North America|USA|
MG849389.1|North America|USA|
MG848899.1|North America|USA|
MG848162.1|North America|USA|
MG849462.1|North America|USA|
MG849033.1|North America|USA|
AF472508.1|||
HQ644238.1|||
HQ644240.1|||
MG849121.1|North America|USA|
MG850657.1|North America|USA|
MG848444.1|North America|USA|
MG849777.1|North America|USA|
MG848500.1|North America|USA|
MG849137.1|North America|USA|
MG848728.1|North America|USA|
MG850056.1|North America|USA|
MG848706.1|North America|USA|
MG848890.1|North America|USA|
MG849696.1|North America|USA|
MG848225.1|North America|USA|
MG848652.1|North America|USA|
MG848992.1|North America|USA|
MG849753.1|North America|USA|
AF536180.1|||
MG848303.1|North America|USA|
KU053906.1|||
MG849164.1|North America|USA|
MG849623.1|North America|USA|
MG850768.1|North America|USA|
MG847990.1|North America|USA|
MG849132.1|North America|USA|
MG847628.1|North America|USA|
MG847795.1|North America|USA|
MG848471.1|North America|USA|
MG850011.1|North America|USA|
MG849207.1|North America|USA|
MG848083.1|North America|USA|
MG847881.1|North America|USA|
MG849314.1|North America|USA|
MG849328.1|North America|USA|
OP728374.1|Europe|United Kingdom|2018
MG848527.1|North America|USA|
MG849053.1|North America|USA|
MG849326.1|North America|USA|
MG850677.1|North America|USA|
MG848384.1|North America|USA|
MG850617.1|North America|USA|
MG849749.1|North America|USA|
MG849839.1|North America|USA|
HM057182.1|South America|Brazil|2007-03
KU053908.1|||
KU053909.1|||
KU053910.1|||
KU053913.1|||
KU053914.1|||
KU053912.1|||
KU053911.1|||
KU053915.1|||
OP751587.1|Europe|United Kingdom|2016
HQ644298.1|||
0.01
